# Unusual Structures and Cytotoxicities of Chitonoidosides A, A_1_, B, C, D, and E, Six Triterpene Glycosides from the Far Eastern Sea Cucumber *Psolus chitonoides*

**DOI:** 10.3390/md19080449

**Published:** 2021-08-05

**Authors:** Alexandra S. Silchenko, Anatoly I. Kalinovsky, Sergey A. Avilov, Pelageya V. Andrijaschenko, Roman S. Popov, Pavel S. Dmitrenok, Ekaterina A. Chingizova, Vladimir I. Kalinin

**Affiliations:** G.B. Elyakov Pacific Institute of Bioorganic Chemistry, Far Eastern Branch of the Russian Academy of Sciences, Pr. 100-letya Vladivostoka 159, 690022 Vladivostok, Russia; silchenko_alexandra_s@piboc.dvo.ru (A.S.S.); kaaniv@piboc.dvo.ru (A.I.K.); avilov_sa@piboc.dvo.ru (S.A.A.); andrijashchenko_pv@piboc.dvo.ru (P.V.A.); popov_rs@piboc.dvo.ru (R.S.P.); paveldmt@piboc.dvo.ru (P.S.D.); chingizova_ea@piboc.dvo.ru (E.A.C.)

**Keywords:** *Psolus chitonoides*, triterpene glycosides, chitonoidosides, sea cucumber, cytotoxic activity

## Abstract

Six new triterpene tetra-, penta- and hexaosides, chitonoidosides A (**1**), A_1_ (**2**), B (**3**), C (**4**), D (**5**), and E (**6**), containing one or two sulfate groups, have been isolated from the Far-Eastern sea cucumber *Psolus chitonoides*, collected near Bering Island (Commander Islands) from the depth of 100–150 m. Three of the isolated compounds (**1**, **3** and **6**) are characterized by the unusual aglycone of new type having 18(20)-ether bond and lacking a lactone in contrast with wide spread holostane derivatives. Another unexpected finding is 3-*O*-methylxylose residue as a terminal unit in the carbohydrate chains of chitonoidosides B (**3**), C (**4**), and E (**6**), which has never been found before in the glycosides from holothurians belonging to the *Psolidae* family. Moreover, this monosaccharide is sulfated in the compound **4** into unprecedented 3-*O*-methylxylose 4-*O*-sulfate residue. Chitonoidoside C (**4**) is characterized by tetrasaccharide moiety lacking a part of the bottom semi-chain, but having disaccharide fragment attached to C-4 of Xyl1. Such architecture is not common in sea cucumber glycosides. Cytotoxic activities of the compounds **1**–**5** against mouse and human erythrocytes and human cancer cell lines: adenocarcinoma HeLa, colorectal adenocarcinoma DLD-1, and leukemia promyeloblast HL-60 cells were studied. The cytotoxic effect of chitonoidoside d (**5**) was the most significant in this series due to the presence of pentasaccharide disulfated sugar chain in combination with holostane aglycone. Surprisingly, the glycosides **1** and **3**, comprising the new aglycone without γ-lactone, demonstrated similar activity to the known compounds with holostane aglycones. Chitonoidoside C (**4**) was less cytotoxic due to the different architecture of the carbohydrate chain compared to the other glycosides and probably due to the presence of a sulfate group at C-4 in 3-*O*-MeXyl4.

## 1. Introduction

Triterpene glycosides, biosynthesized by the sea cucumbers, are natural compounds characterized by the diverse bioactivity [1,2,3,4,5]. Some of them are candidates to be used as new immunomodulators and anti-tumor drugs [6,7,8], which are urgently needed because cancer is one of the most important global health problems. The isolation of glycosides from different sea cucumber species provides a unique opportunity to expand knowledge on their structural diversity and discover new unusual substances. The comparative structural analysis of a significant number of the isolated glycosides leads to the reconstruction of the sequences of biosynthetic transformations of their aglycones and carbohydrate chains during biosynthesis that can be reflected in the metabolic networks constructed for different species of the sea cucumbers [9,10]. The studies on biological activity of the glycosides reveal the peculiarities of «structure–activity relationships» demonstrating the significance of different structural parts of glycosides for the realization of their physiological action. These data indicate the relevance of searching for new glycosides and studying their biological activities.

The genus *Psolus* comprises 58 species, and only three of them were chemically investigated up to the present time: *P. fabricii* [10,11,12,13,14], *P. eximius* [15], and *P. patagonicus* [16,17]. As result of the studies on the fourth representative of the genus, *P. chitonoides*, six new triterpene glycosides, chitonoidosides A (**1**), A_1_ (**2**), B (**3**), C (**4**), D (**5**), and E (**6**) were isolated and their chemical structures elucidated by the analyses of the ^1^H, ^13^C NMR, 1D TOCSY and 2D NMR (^1^H,^1^H-COSY, HMBC, HSQC, ROESY) spectra as well as HR-ESI mass spectra. All of the original spectra are presented in Figures S1–S48 in Supplementary data. The hemolytic activities against mouse and human erythrocytes, cytotoxic activities against human adenocarcinoma HeLa, colorectal adenocarcinoma DLD-1, and leukemia promyeloblast HL-60 cells are reported.

## 2. Results and Discussion

### 2.1. Structural Elucidation of the Glycosides

**Figure 1:**
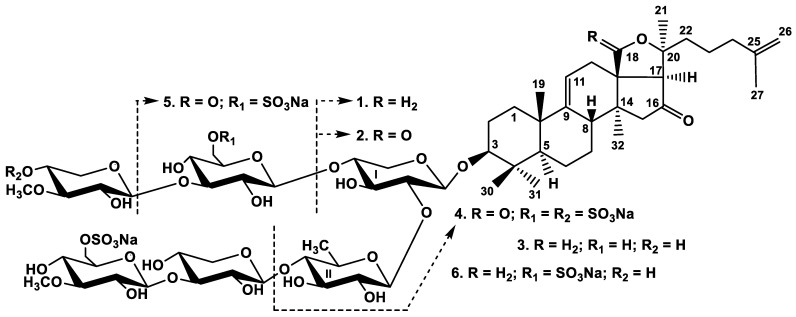
Chemical structures of glycosides isolated from *Psolus chitonoides*: **1**—chitonoidoside A; **2**—chitonoidoside A_1_; **3**—chitonoidoside B; **4**—chitonoidoside C; **5**—chitonoidoside D; **6**—chitonoidoside E.

The concentrated ethanolic extract of the sea cucumber *Psolus chitonoides* was chromatographed on a Polychrom-1 column (powdered Teflon, Biolar, Latvia). The glycosides were eluted after using water as a mobile phase with 50% EtOH and separated by repeated chromatography on Si gel columns with the eluents CHCl_3_/EtOH/H_2_O (100:75:10), (100:100:17), and (100:125:25) to give three fractions (I–III). The individual compounds **1**–**6** (Figure 1) were isolated by HPLC of the obtained fractions on silica-based column Supelcosil LC-Si (4.6 × 150 mm) and reversed-phase semipreparative columns Phenomenex Synergi Fusion RP (10 × 250 mm) or Supelco Ascentis RP-Amide (10 × 250 mm).

All the configurations of monosaccharides in all glycosides were assigned to D-series along with biogenetical analogies with all known sea cucumber triterpene glycosides.

The molecular formula of chitonoidoside A (**1**) was determined to be C_53_H_83_O_23_SNa from the [M_Na_−Na]^−^ ion peak at *m/z* 1119.5049 (calc. 1119.5051) in the (−)HR-ESI-MS and [M_Na_+Na]^+^ ion peak at *m/z* 1165.4816 (calc. 1165.4836) in the (+)HR-ESI-MS. The ^1^H and ^13^C NMR spectra of the carbohydrate chain of chitonoidoside A (**1**) (Table 1, Figures S1–S6) demonstrated four characteristic doublets at δ_H_ 4.82–5.30 (*J* = 7.2–8.6 Hz) and, corresponding to them, signals of anomeric carbons at δ_C_ 104.2–105.5 indicating the presence of four monosaccharide residues, bonded to each other by *β*-glycosidic linkages. Analysis of the ^1^H,^1^H-COSY, HSQC, and 1D TOCSY spectra showed that two xylose (Xyl1 and Xyl3), one quinovose (Qui2), and one 3-*O*-methylglucose (MeGlc4) residues are present in the carbohydrate chain of chitonoidoside A (**1**). The signal of C-6 MeGlc4 was observed at δ_C_ 66.6, due to α-shifting effect of a sulfate group at this position. The signal of C-4 Xyl1 (δ_C_ 70.7) showed the presence of a free hydroxy group at this carbon atom in **1**. The positions of interglycosidic linkages were established by the ROESY and HMBC spectra (Table 1). Thus, despite the typical sugar composition for the sea cucumber glycosides, the oligosaccharide chain of **1** turned out to be new.

Analysis of the NMR spectra of the aglycone part of **1** (Table 2, Figures S1–S6) indicated the presence of C-30 triterpene nucleus with the 9(11)-double bond (characteristic signals δ_C_ 151.0 (C-9) and 114.7 (C-11)) and the C-16 keto-group (δ_C_ 215.9 (C-16)). These positions were confirmed by the HMBC correlations H-8, H_2_-12, H_3_-19/C-9, H_2_-12/C-11, and H_2_-15, H-17/C-16. The signal of C-18 at δ_C_~178–180 characteristic of 18(20)-lactone in holostane aglycones was absent in the ^13^C NMR spectrum of compound **1**.

The singlet signal of H-17 at δ_H_ 2.38 indicated its bonding to three quaternary carbons C-13, C-16, and C-20. The signal of C-20 at δ_C_ 86.2 was deduced by the characteristic HMBC correlation between the signal of methyl group H_3_-21 and C-20. Whereas H_2_-12 (δ_C_ 2.37 (m) and 2.27 (dd, *J* = 5.4; 16.9 Hz)) and H-17 were correlated in the HMBC spectrum with the signal at δ_C_ 73.8 assigned to the methylene group, having the proton signals at δ_H_ 4.08 (d, H-18a) and 3.71 (d, H-18b), the position of this group was established as C-18. The cross-peaks H-8β, H-12a,b, and H-15β/H-18 in the ROESY spectrum confirmed this. Based on the deshielded signals of C-18 and C-20, it was supposed that they linked by ether bonds. The configurations of all the asymmetric centers were the same as in holostane aglycones, which was confirmed by the ROESY spectrum. Thus, a new type of non-holostane aglycone lacking a lactone was discovered in the glycoside **1** of *P. chitonoides*.

The (−)ESI-MS/MS of **1** demonstrated the fragmentation of [M_Na_−Na]^−^ ion at *m/z* 1119.5 resulting in the appearance of the fragment ion-peaks at *m/z* 665.1 [M_Na_−Na–C_30_H_46_O_3_ (Agl)]^−^, corresponding to the aglycone loss, 533.1 [M_Na_−Na–C_30_H_46_O_3_ (Agl)−C_5_H_8_O_4_ (Xyl)]^−^, 387.1 [M_Na_−Na–C_30_H_46_O_3_ (Agl)−C_5_H_8_O_4_ (Xyl)−C_6_H_10_O_4_ (Qui)]^−^, 255.0 [M_Na_−Na–C_30_H_46_O_3_ (Agl)−C_5_H_8_O_4_ (Xyl)−C_6_H_10_O_4_ (Qui)−C_5_H_8_O_4_ (Xyl)]^−^. The (+)ESI-MS/MS of **1** demonstrated the fragmentation of [M_Na_+Na]^+^ ion at *m/z* 1165.5. The peak of fragment ion had *m/z* 1045.5 [M_Na_+Na−NaSO_4_−H]^+^, corroborating the presence of a sulfate group. Other fragment ion peaks illustrated the same patterns that were observed in (−)ESI-MS/MS (see Materials and Methods) corroborating both of the aglycone and carbohydrate chain structures in chitonoidoside A (**1**).

All of these data indicate that chitonoidoside A (**1**) is 3*β*-*O*-[6-*O*-sodium sulfate-3-*O*-methyl-*β*-d-glucopyranosyl-(1→3)-*β*-d-xylopyranosyl-(1→4)-*β*-d-quinovopyranosyl-(1→2)-*β*-d-xylopyranosyl]-16-oxo-18(20)-epoxylanosta-9(11),25(26)-diene.

The molecular formula of chitonoidoside A_1_ (**2**) was determined to be C_53_H_81_O_24_SNa from the [M_Na_−Na]^−^ ion peak at *m/z* 1133.4856 (calc. 1133.4844) in the (−)HR-ESI-MS and [M_Na_+Na]^+^ ion peak at *m/z* 1179.4620 (calc. 1179.4628) in the (+)HR-ESI-MS. The NMR spectra corresponding to the carbohydrate part of **2** were close to those of **1**, indicating the identity of their sugar chains (Table S1, Figures S8–S13). Analysis of the ^1^H and ^13^C NMR spectra of the aglycone part of **2** indicated the presence of earlier known and broadly distributed in the sea cucumber glycosides holostane aglycone holotoxinogenin, first found in *Apostichopus japonicus* [18] (Table 3, Figures S8–S13), which differed from the aglycone of **1** by the presence of 18(20)-lactone. The same aglycone was identified in chitonoidosides C (**4**) and D (**5**) (Tables S3, S4, Figures S24–S29 and S32–S37).

The (−)ESI-MS/MS of **2** demonstrated the fragmentation of [M_Na_−Na]^−^ ion with *m/z* 1133.5 The peak of fragment ion, observed at *m/z* 665.1 [M_Na_−Na–C_30_H_44_O_4_ (Agl)]^−^, corresponded to the aglycone loss. The subsequent fragmentation led to the appearance of the same fragmentary ion-peaks at *m/z* 533.1, 387.1 and 255.0 that were observed in the MS/MS of **1**, corroborating the identity of the carbohydrate chains of chitonoidosides A_1_ (**2**) and A (**1**).

All these data indicate that chitonoidoside A_1_ (**2**) is 3*β*-*O*-[6-*O*-sodium sulfate-3-*O*-methyl-*β*-d-glucopyranosyl-(1→3)-*β*-d-xylopyranosyl-(1→4)-*β*-d-quinovopyranosyl-(1→2)-*β*-d-xylopyranosyl]-16-oxo-holosta-9(11),25(26)-diene.

The molecular formula of chitonoidoside B (**3**) was determined to be C_65_H_103_O_32_SNa from the [M_Na_−Na]^−^ ion peak at *m/z* 1427.6157 (calc. 1427.6159) and [M_Na_+Na]^+^ ion-peak at *m/z* 1473.5925 (calc. 1473.5943) in the (−) and (+)HR-ESI-MS, correspondingly. The ^1^H and ^13^C NMR spectra of the carbohydrate part of chitonoidoside B (**3**) showed six characteristic doublets at δ_H_ 4.67–5.12 (*J* = 7.6–8.7 Hz), correlated by the HSQC spectrum with corresponding anomeric carbons at δ_C_ 102.2–105.3. These signals were indicative of a hexasaccharide chain and *β*-configurations of glycosidic bonds (Table 4, Figures S15–S22). The signals of each monosaccharide unit were deduced as an isolated spin system based on the ^1^H,^1^H-COSY and 1D TOCSY spectra. Further analysis of the HSQC and ROESY spectra of **3** resulted in the assigning of the monosaccharide residues as two xylose (Xyl1 and Xyl3), one quinovose (Qui2), one glucose (Glc5), one 3-*O*-methylglucose (MeGlc4), and one 3-*O*-methylxylose (MeXyl6) residues. The positions of interglycosidic linkages, established by the ROESY and HMBC spectra of **3**, were characteristic of the majority of hexaosides from the sea cucumbers (Table 4).

The signal of C-6 MeGlc4 in the ^13^C NMR spectrum of **3** was deshielded to δ_C_ 67.1 due to α-shifting effect of the sulfate group at this position. 3-*O*-methylxylose residue in the upper semi-chain of **3** is not often found in the sea cucumber glycosides and was discovered earlier in some representatives of such taxa as *Synallactes nozawai* (order Synallactida) [19] and four species of the order Dendrochirotida: *Eupentacta fraudatrix* [5] and *E. pseudoquinquesemita* [20] (family Sclerodactylidae), *Pentamera calcigera* [21,22], and *Thyone aurea* [23] (family Phyllophoridae). None of the previously investigated species of genus *Psolus* contained this residue in the glycosides, so its finding was a surprise.

The NMR data of the aglycone part of **3** indicated the identity of the aglycone to that of chitonoidoside A (**1**) (Table S2, Figures S15–S20).

The (−)ESI-MS/MS of **3** demonstrated the fragmentation of [M_Na_−Na]^−^ ion with *m/z* 1427.6. The peaks of fragment ions were observed at *m/z* 1281.5 [M_Na_−Na–C_6_H_11_O_4_ (MeXyl)+H]^−^, 1119.5 [M_Na_−Na–C_6_H_11_O_4_ (MeXyl)−C_6_H_10_O_5_ (Glc)+H]^−^. The (+)ESI-MS/MS of **3** demonstrated the fragmentation of [M_Na_+Na]^+^ ion with *m/z* 1473.5 resulting in the appearance of the peaks of fragment ions at *m/z* 1354.5 [M_Na_+Na−NaHSO_4_]^+^, 1327.5 [M_Na_+Na−C_6_H_11_O_4_ (MeXyl)+H]^+^, 1194.5 [M_Na_+Na−C_7_H_11_O_8_SNa (MeGlcSO_3_Na)]^+^, 1165.5 [M_Na_+Na−C_6_H_11_O_4_ (MeXyl)−C_6_H_10_O_5_ (Glc)+H]^+^, 1062.5 [M_Na_+Na−C_7_H_11_O_8_SNa (MeGlcSO_3_Na)−C_5_H_8_O_4_ (Xyl)]^+^, 917.5 [M_Na_+Na−C_7_H_11_O_8_SNa (MeGlcSO_3_Na)−C_5_H_8_O_4_ (Xyl)−C_6_H_10_O_4_ (Qui)]^+^, confirming the sequence of monosaccharides in the carbohydrate chain of **3**.

All these data indicate that chitonoidoside B (**3**) is 3*β*-*O*-{6-*O*-sodium sulfate-3-*O*-methyl-*β*-d-glucopyranosyl-(1→3)-*β*-d-xylopyranosyl-(1→4)-*β*-d-quinovopyranosyl-(1→2)-[3-*O*-methyl-*β*-d-xylopyranosyl-(1→3)-*β*-d-glucopyranosyl-(1→4)]-*β*-d-xylopyranosyl}-16-oxo-18(20)-epoxylanosta-9(11),25(26)-diene.

The molecular formula of chitonoidoside C (**4**) was determined to be C_53_H_80_O_27_S_2_Na_2_ from the [M_2Na_−Na]^−^ ion peak at *m/z* 1235.4256 (calc. 1235.4232) and [M_2Na_−2Na]^2^^−^ ion peak at *m/z* 606.2183 (calc. 606.2170) in the (−)HR-ESI-MS. The ^1^H and ^13^C NMR spectra corresponding to the carbohydrate chain of chitonoidoside C (**4**) (Table 5, Figures S24–S30) demonstrated four signals of anomeric protons at δ_H_ 4.68–5.08 (d, *J* = 6.6–8.2 Hz) and corresponding to them signals of anomeric carbons at δ_C_ 102.2–104.9 deduced by the HSQC spectrum. These signals indicated the presence of tetrasaccharide moiety with *β*-glycosidic bonds. The signals of each sugar residue were assigned by the analysis of the ^1^H,^1^H-COSY, 1D TOCSY, ROESY, and HSQC spectra, enabling identification of the monosaccharide units in the chain of **4** as xylose (Xyl1), quinovose (Qui2), glucose (Glc3), and 3-*O*-methylxylose (MeXyl4). Analysis of the glycosidic bond positions by the ROESY and HMBC spectra showed that quinovose residue was terminal in the bottom semi-chain because only the correlation between the anomeric proton H-1 Qui2 (δ_H_ 5.06, d, *J* = 7.5 Hz) and H-2 (C-2) Xyl1 took place. Actually, the glycosylation effects were not observed for C-3–C-5 Qui2 in the ^13^C NMR spectrum of **4**. In fact, the signal C-4 Qui2 was shielded to δ_C_ 76.2 when compared with the same signal in the spectrum of **3** (δ_C_ 85.6), and the signals of C-3 and C-5 Qui2 were observed at δ_C_ 76.8 and 72.5 instead of δ_C_ 74.9 and 71.4 in the spectrum of **3**, correspondingly. Therefore, the remaining monosaccharide residues formed the upper semi-chain. The cross-peaks were observed between H-1 Glc3 and H-4 (C-4) Xyl1 and between H-1 MeXyl4 and H-3 (C-3) Glc3 in the ROESY and HMBC spectra, correspondingly. Therefore, the architecture of carbohydrate chain of **4** was the same as in kurilosides of the group C from *Thyonidium kurilensis* [24]. The comparison of the ^13^C NMR spectra of sugar chains of chitonoidoside C (**4**) and kuriloside C_1_ showed the coincidence of the signals of three monosachharide units with the exception of the signals of (fourth) terminal sugar. The comparison of the signals of 3-*O*-methylxylose, attached to C-3 of Glc in the upper semi-chain, in the ^13^C NMR spectra of **3** and **4** revealed their differences due the presence of the sulfate group attached to C-4 of 3-*O*-methylxylose in **4**. Actually, the corresponding signal was deshielded (δ_C_ 75.5) and the signals of C-3 and C-5 MeXyl4 were shielded (δ_C_ 84.1 and 64.5) due to α- and β-effects of the sulfate group. The sulfated 3-*O*-methylxylose residue was for the first time found in the glycosides from Holothuroidea. Additionally, C-4 in terminal sugars is not a common position of the sulfation in the glycosides in contrast to C-6 of terminal 3-*O*-methylglucose or glucose residues. The glycosides characterized by the sulfate group at C-4 of a glucose residue were so far recently found only in *Psolus fabricii* [14].

The second sulfate group in the sugar chain of **4** was attached to C-6 Glc3 (δ_C_ 67.2). Thus, the new carbohydrate chain of chitonoidoside C (**4**) combined the features of the glycosides from *T. kurilensis* and *P. fabricii* as a result of parallel chemical evolution.

The aglycone part of chitonoidoside C (**4**) was identical to that of chitonoidoside A_1_ (**2**). This was supposed based on the coincidence of their NMR spectra (Table S3, Figures S24–S29).

The (−)ESI-MS/MS of chitonoidoside C (**4**) demonstrated the fragmentation of [M_2Na_−Na]^−^ ion at *m/z* 1235.4. The peaks of fragment ions were observed at *m/z* 1115.5 [M_2Na_−Na–NaHSO_4_]^−^, 1089.4 [M_2Na_−Na−C_6_H_10_O_4_ (Qui)]^−^, 969.4 [M_2Na_−Na−NaHSO_4_–C_6_H_10_O_4_ (Qui)]^−^, 841.4 [M_2Na_−Na−C_6_H_10_O_7_SNa (MeXylOSO_3_)−C_6_H_10_O_4_ (Qui)+H]^−^, 517.1 [M_2Na_−Na–C_30_H_44_O_4_ (Agl)−C_6_H_10_O_7_SNa (MeXylOSO_3_)−H]^−^, corroborating the structure established based on NMR analyses.

All these data indicate that chitonoidoside C (**4**) is 3*β*-*O*-{*β*-d-quinovopyranosyl-(1→2)-[4-*O*-sodium sulfate-3-*O*-methyl-*β*-d-xylopyranosyl-(1→3)-6-*O*-sodium sulfate*-β*-d-glucopyranosyl-(1→4)]-*β*-d-xylopyranosyl}-16-oxo-holosta-9(11),25(26)-diene.

The molecular formula of chitonoidoside D (**5**) was determined to be C_59_H_90_O_32_S_2_Na_2_ from the [M_2Na_−Na]^−^ ion peak at *m/z* 1397.4706 (calc. 1397.4760), [M_2Na_−2Na]^2^^−^ ion peak at *m/z* 687.2412 (calc. 687.2434) in the (−)HR-ESI-MS. The presence of two-charged ions in the MS spectra indicated that chitonoidoside D (**5**) contains two sulfate groups. The ^1^H and ^13^C NMR spectra of the carbohydrate part of chitonoidoside D (**5**) (Table 6, Figures S32–S38) demonstrated five characteristic doublets at δ_H_ 4.67–5.11 (d, *J* = 7.0–8.1 Hz) and, corresponding to them, signals of anomeric carbons at δ_C_ 102.7–104.8 deduced by the HSQC spectrum, that indicated the presence of five monosaccharide residues in **5**. The monosaccharide composition of **5** was determined to include two xylose (Xyl1 and Xyl3), quinovose (Qui2), glucose (Glc5), and 3-*O*-methylglucose (MeGlc4) residues. The positions of glycosidic bonds established by the ROESY and HMBC spectra were typical and showed bottom semi-chains composed of three monosaccharide units and the upper semi-chain—from two monosaccharide units, forming the chain with the common architecture. The comparison of the ^13^C NMR spectrum of the carbohydrate chain of cladolosides of the group I, having the same sugar composition and architecture [25], with the spectrum of chitonoidoside D (**5**) showed they differed in the signals C-5 and C-6 Glc5 only, shifted in the spectrum of **5** due to the presence of the second sulfate group (δ_C_ 75.8 (C-5 Glc5) and 67.2 (C-6 Glc5)) (Table 6). Thus, chitonoidoside D (**5**) contained a new branched disulfated pentasaccharide carbohydrate chain.

The aglycone part of chitonoidoside D (**5**) (Table S4, Figures S32–S37) was identical to that of chitonoidosides A_1_ (**2**) and C (**4**), which was confirmed by the closeness of their NMR spectra.

The (−)ESI-MS/MS of chitonoidoside D (**5**) demonstrated the fragmentation of [M_2Na_−Na]^−^ ion at *m/z* 1397.5. The peaks of fragment ions were observed at *m/z* 1277.5 [M_2Na_−Na–NaHSO_4_]^−^, 1119.5 [M_2Na_−Na–C_7_H_11_O_8_SNa (MeGlcOSO_3_)]^−^, 987.4 [M_2Na_−Na–C_7_H_11_O_8_SNa (MeGlcOSO_3_)−C_5_H_8_O_4_ (Xyl)]^−^, 841.3 [M_2Na_−Na–C_7_H_11_O_8_SNa (MeGlcOSO_3_)−C_5_H_8_O_4_ (Xyl)–C_6_H_10_O_4_ (Qui)]^−^, corroborating the structure of the oligosaccharide chain of **5**.

All these data indicate that chitonoidoside D (**5**) is 3*β*-*O*-{6-*O*-sodium sulfate-3-*O*-methyl-*β*-d-glucopyranosyl-(1→3)-*β*-D-xylopyranosyl-(1→4)-*β*-d-quinovopyranosyl-(1→2)-[6-*O*-sodium sulfate*-β*-d-glucopyranosyl-(1→4)]-*β*-d-xylopyranosyl}-16-oxo-holosta-9(11),25(26)-diene.

The molecular formula of chitonoidoside E (**6**) was determined to be C_65_H_102_O_35_S_2_Na_2_ from the [M_2Na_−Na]^−^ ion peak at *m/z* 1529.5567 (calc. 1529.5546) and [M_2Na_−2Na]^2^^−^ ion peak at *m/z* 753.2836 (calc. 753.2827) in the (−)HR-ESI-MS. The ^1^H and ^13^C NMR spectra, corresponding to the carbohydrate chain of **6** (Table 7, Figures S40–S47), demonstrated six characteristic doublets at δ_H_ 4.67–5.12 (*J* = 6.9–7.8 Hz) correlated with corresponding anomeric carbons at δ_C_ 102.2–105.2 by the HSQC spectrum, indicating the presence of a hexasaccharide moiety and *β*-configurations of glycosidic bonds. The analysis of the ^1^H,^1^H-COSY, 1D TOCSY, HSQC, and ROESY spectra of **6** revealed the same monosaccharide composition and positions of glycosidic bonds as in the carbohydrate chain of chitonoidoside B (**3**). Actually, their ^13^C NMR spectra were almost coincident differing by the signals C-5 and C-6 of Glc5. The second sulfate group, attached to C-6 of Glc5 in **6**, caused the shifting of the signals C-5 Glc5 to δ_C_ 75.5 and C-6 Glc5 to δ_C_ 67.1. The difference in the MS spectra of **3** and **6** in 102 *amu* corroborated this.

The NMR data of the aglycone part of **6** indicated the identity of the aglycone to that of chitonoidoside A (**1**) and B (**3**) (Table S5, Figures S40–S45).

The (−)ESI-MS/MS of **6** demonstrated the fragmentation of [M_2Na_−Na]^−^ ion at *m/z* 1529.5. The peaks of fragment ions were observed at *m/z*: 1409.5 [M_2Na_−Na–NaHSO_4_]^−^, 1383.5 [M_2Na_−Na–C_6_H_11_O_4_ (MeXyl)+H]^−^, 1263.5 [M_2Na_−Na–C_6_H_11_O_4_ (MeXyl) –NaHSO_4_]^−^, 1251.5 [M_2Na_−Na–C_7_H_11_O_8_SNa (MeGlcOSO_3_)]^−^, 1119.5 [M_2Na_−Na–C_7_H_11_O_8_SNa (MeGlcOSO_3_)−C_5_H_8_O_4_ (Xyl)]^−^, 973.4 [M_2Na_−Na–C_7_H_11_O_8_SNa (MeGlcOSO_3_)−C_5_H_8_O_4_ (Xyl)–C_6_H_10_O_4_ (Qui)]^−^, 827.4 [M_2Na_−Na–C_7_H_11_O_8_SNa (MeGlcOSO_3_)−C_5_H_8_O_4_ (Xyl)–C_6_H_10_O_4_ (Qui) –C_6_H_11_O_4_ (MeXyl)+H]^−^, confirming the sequence of the monosaccharide residues in the chain of **6**.

All these data indicate that chitonoidoside E (**6**) is 3*β*-*O*-{6-*O*-sodium sulfate-3-*O*-methyl-*β*-d-glucopyranosyl-(1→3)-*β*-d-xylopyranosyl-(1→4)-*β*-d-quinovopyranosyl-(1→2)-[3-*O*-methyl-*β*-d-xylopyranosyl-(1→3)-6-*O*-sodium sulfate-*β*-d-glucopyranosyl-(1→4)]-*β*-d-xylopyranosyl}-16-oxo-18(20)-epoxylanosta-9(11),25(26)-diene.

### 2.2. Bioactivity of the Glycosides

Cytotoxic activities of the compounds **1**–**5** against mouse and human erythrocytes and human cancer cell lines: adenocarcinoma HeLa, colorectal adenocarcinoma DLD-1, and leukemia promyeloblast HL-60 cells were studied (Table 8). The known earlier strong hemolytic psolusoside L was used as the positive control [14] as well as cisplatin. Mouse erythrocytes are traditionally used as the model for studying structure–activity relationships of glycosides. This time, we compared the hemolytic action of the glycosides against mouse and human red blood cells. A very close effect for both cell lines was revealed, obviously due to their similar membrane composition. In addition, erythrocytes were the most sensitive to the membranolytic action of tested compounds compared to the cancer cells. Thus, the glycosides **1**–**5** demonstrated high hemolytic and, in most cases, moderate cytotoxic activity, illustrating the structure–activity relationships (SARs) of the glycosides. HL-60 cells were more strongly affected by the glycosides than the other cancer cells, making this cell line a possible target for the studies of the glycosides as anticancer drugs.

Noticeably, the glycosides **1** and **3**, comprising the new aglycone without a lactone, demonstrated the similar activity as the compounds with holostane aglycones. Chitonoidoside C (**4**) were the less cytotoxic compound due to the different architecture of the carbohydrate chain compared to the other glycosides and probably due to the presence of a sulfate group at C-4 of 3-*O*-MeXyl4. The cytotoxic effect of chitonoidoside D (**5**) was the most significant in this series and comparable to that of positive control (psolusoside L and cisplatin). Actually, these glycosides are structurally differing by the third monosaccharide unit and in the absence/presence of the third sulfate group in their pentasaccharide chains.

### 2.3. Biosynthesis of the New Type of Non-Holostane Aglycone of the Glycosides 1, 3, and 6

Holostane type aglycones (having 18(20)-lactone), such as holotoxinogenin which is part of chitonoidosides A_1_ (**2**), C (**4**), and D (**5**), are predominant in the sea cucumber glycosides. Much less frequent non-holostane aglycones belong to some types: (i) having 18(16)-lactone and (ii) without a lactone. Both of these types can have normal or shortened side chains [26]. Noticeably, it was discovered in chitonoidosides A (**1**), B (**3**), and E (**6**) that non-holostane aglycone structurally differs from the non-holostane aglycones lacking a lactone found so far, which is characterized by the tetracyclic triterpene nuclei. This new aglycone has the additional perhydrofurane cycle.

**Figure 2:**
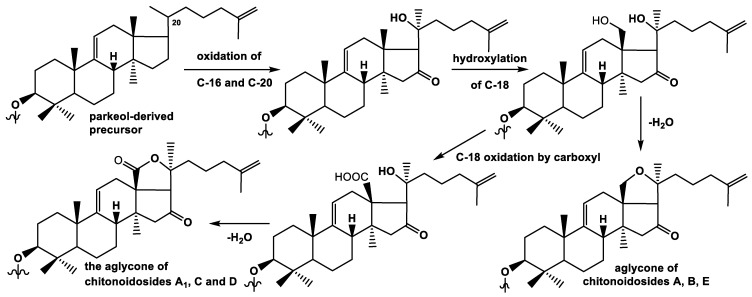
Biosynthetic steps of holostane and new non-holostane type aglycones formation.

The diverse types of the triterpene aglycones are formed in the process of biosynthesis through different sequences of oxidative stages, which can be shifted in time in relation to each other. The biosynthesis of the major part of the aglycones occurs through the oxidation of the C-20 atom in the lanostane precursors, while position 18 remains unoxidized (Figure 2). The oxidation of a C-18 methyl group into hydroxymethyl is the next step. The corresponding hot metabolite was isolated by us recently from *E. fraudatrix* [27]. The subsequent oxidation of C-18 hydroxymethyl by carboxyl followed by lactonization leads to 18(20)-lactone formation. This is the general pathway of biosynthesis of holostane aglycones, including holotoxinogenin with additionally oxidized C-16 into a 16-oxo-group found in glycosides **2**, **4**, and **5** and many other glycosidic metabolites of sea cucumbers.

In the process of biosynthesis of the aglycone of chitonoidosides **1**, **3**, **6**, containing an 18(20)-ether bond, the stage of C-18 carboxylation is dropped out, and the intramolecular dehydration of 18,20-hydroxylated precursor leads to the formation of epoxy-cycle (Figure 2).

## 3. Materials and Methods

### 3.1. General Experimental Procedures

Specific rotation, PerkinElmer 343 Polarimeter (PerkinElmer, Waltham, MA, USA); NMR, Bruker Avance III 700 Bruker FT-NMR (Bruker BioSpin GmbH, Rheinstetten, Germany) (700.00/176.03 MHz) (^1^H/^13^C) spectrometer; ESI MS (positive and negative ion modes), Agilent 6510 Q-TOF apparatus (Agilent Technology, Santa Clara, CA, USA), sample concentration 0.01 mg/mL; HPLC, Agilent 1260 Infinity II with a differential refractometer (Agilent Technology, Santa Clara, CA, USA); columns Supelcosil LC-Si (4.6 × 150 mm, 5 μm) and Ascentis RP-Amide (10 × 250 mm, 5 μm) (Supelco, Bellefonte, PA, USA), Phenomenex Synergi Fusion RP (10 × 250 mm, 5 μm) (Phenomenex, Torrance, CA, USA).

### 3.2. Animals and Cells

Specimens of the sea cucumber *Psolus chitonoides* (family Psolidae; order Dendrochirotida) were collected in Bering Sea during the 14th expedition on research vessel “Akademik Oparin” on 24 August 1991, near the northern coast of Bering Island (Commander Islands). Sampling was performed by the Sigsby trawl at the depth 100–150 m. The animals were identified by Alexey V. Smirnov, Zoological Institute Russian Academy of Sciences. Voucher specimens are preserved at Zoological Institute RAS, St. Petersburg, Russia.

CD-1 mice, weighing 18–20 g, were purchased from RAMS ‘Stolbovaya’ vivarium (Stolbovaya, Moscow District, Russia) and kept at the animal facility in standard conditions. All experiments were performed following the protocol for animal study approved by the Ethics Committee of the Pacific Institute of Bioorganic Chemistry No. 0085.19.10.2020. All experiments were conducted in compliance with all of the rules and international recommendations of the European Convention for the Protection of Vertebrate Animals Used for Experimental Studies.

Human erythrocytes were obtained from Vladivostok blood transfusion station. Human adenocarcinoma cell line HeLa was provided by the N.N. Blokhin Russian Oncology Center (Russian Academy of Medical Sciences, Moscow, Russia). Human colorectal adenocarcinoma cell line DLD-1 CCL-221™ and human promyeloblast cell line HL-60 CCL-240 were obtained from ATCC (Manassas, VA, USA). Cell line HeLa was cultured in DMEM medium (Gibco Dulbecco’s Modified Eagle Medium) containing 10% fetal bovine serum (FBS) (Biolot, St. Petersburg, Russia) and 1% penicillin G sodium salt/streptomycin sulfate (Biolot, St. Petersburg, Russia). Cell lines DLD-1 and HL-60 were cultured in RPMI medium containing 10% fetal bovine serum (FBS) (Biolot, St. Petersburg, Russia) and 1% penicillin/streptomycin (Biolot, St. Petersburg, Russia). All cell lines were incubated at 37 °C in a humidified atmosphere with 5% (*v/v*) CO_2_.

### 3.3. Extraction and Isolation

The sea cucumbers were minced and kept in EtOH at +10 °C for several years. Later, they were extracted twice with refluxing 60% EtOH. The combined extracts were concentrated to dryness in vacuum, dissolved in H_2_O, and chromatographed on a Polychrom-1 column (powdered Teflon, Biolar, Latvia). Eluting first the inorganic salts and impurities with H_2_O and then the glycosides with 50% EtOH gave 3200 mg of crude glycoside fraction, which was submitted to the stepwise chromatography on Si gel columns using CHCl_3_/EtOH/H_2_O (100:75:10), (100:100:17) and (100:125:25) as mobile phases to give fractions I–III. HPLC of fraction I on a Phenomenex Synergi Fusion RP (10 × 250 mm) column with CH_3_CN/H_2_O/NH_4_OAc (1 M water solution) (45/54/1) as the mobile phase resulted in the obtaining of 3.2 mg of chitonoidoside A (**1**) as well as another subfraction. Its rechromatography on Supelcosil LC-Si (4.6 × 150 mm) column with CHCl_3_/MeOH/H_2_O (65/15/1) as mobile phase led isolation of pure chitonoidoside A_1_ (**2**) (8.1 mg). The separation of fraction II on a Phenomenex Synergi Fusion RP (10 × 250 mm) column with CH_3_CN/H_2_O/NH_4_OAc (1 M water solution) (43/56/1) as mobile phase resulted in the isolation of some subfractions. The individual chitonoidoside B (**3**) (4.5 mg) was obtained as a result of HPLC of one of these subfractions on the same column with MeOH/H_2_O/NH_4_OAc (1 M water solution) (74/24/2) as a mobile phase. Fraction III was submitted to HPLC on Si-gel column Supelcosil LC-Si (4.6 × 150 mm) using CHCl_3_/MeOH/H_2_O (60/25/4) as a mobile phase and separated into the subfractions 1–6. Further rechromatography of the subfraction 6 on Synergi Fusion RP (10 × 250 mm) column with MeOH/H_2_O/NH_4_OAc (1 M water solution) (70/29/1) gave fractions 6.1–6.8, the repeated HPLC of the fraction 6.8 on the same column with CH_3_CN/H_2_O/NH_4_OAc (1 M water solution) (40/58/2) as a mobile phase gave two only half separated peaks. Peak 6.8.2 was rechromatographed on a Supelcosil LC-Si column with CHCl_3_/MeOH/H_2_O (65/25/3) as a mobile phase, which allowed for isolating 2.9 mg of chitonoidoside C (**4**). The HPLC of the fraction 6.4. on another column Supelco Ascentis RP-Amide (10 × 250 mm) with CH_3_CN/H_2_O/NH_4_OAc (1 M water solution) (30/68/2) as a mobile phase gave chitonoidoside D (**5**) (7 mg). The chromatography of the fraction 4, obtained from the silica-based HPLC column on the reversed phase Ascentis RP-Amide (10 × 250 mm) column with CH_3_CN/H_2_O/NH_4_OAc (1 M water solution) (40/59/1) as mobile phase followed by another mobile phase MeOH/H_2_O/NH_4_OAc (1 M water solution) (68/30/2) gave 6.7 mg of chitonoidoside E (**6**).

#### 3.3.1. Chitonoidoside A (**1**)

Colorless powder; [α]${}_{D}^{20}$ −28° (*c* 0.1, 50% MeOH). NMR: See Table 1 and Table 2, Figures S1–S7. (−)HR-ESI-MS *m/z*: 1119.5049 (calc. 1119.5051) [M_Na_−Na]^−^; (+)HR-ESI-MS *m/z*: 1165.4816 (calc. 1165.4836) [M_Na_+Na]^+^; (−)ESI-MS/MS *m/z*: 665. [M_Na_−Na–C_30_H_46_O_3_ (Agl)]^−^, 533.1 [M_Na_−Na–C_30_H_46_O_3_ (Agl)−C_5_H_8_O_4_ (Xyl)]^−^, 387.1 [M_Na_−Na–C_30_H_46_O_3_ (Agl)−C_5_H_8_O_4_ (Xyl)−C_6_H_10_O_4_ (Qui)]^−^, 255.0 [M_Na_−Na–C_30_H_46_O_3_ (Agl)−C_5_H_8_O_4_ (Xyl)−C_6_H_10_O_4_ (Qui)−C_5_H_8_O_4_ (Xyl)]^−^; (+)ESI-MS/MS *m/z*: 1045.5 [M_Na_+Na−NaSO_4_+H]^+^, 711.1 [M_Na_+Na–C_30_H_46_O_3_ (Agl)]^+^, 609.2 [M_Na_+Na–C_7_H_11_O_8_SNa (MeGlcSO_3_Na)−C_5_H_8_O_4_ (Xyl)−C_6_H_10_O_4_ (Qui) + H]^+^, 579.1 [M_Na_+Na–C_30_H_46_O_3_ (Agl)−C_5_H_8_O_4_ (Xyl)]^+^, 433.0 [M_Na_+Na–C_30_H_46_O_3_ (Agl)−C_5_H_8_O_4_ (Xyl)−C_6_H_10_O_4_ (Qui)]^+^, 301.0 [M_Na_+Na–C_30_H_46_O_3_ (Agl)−C_5_H_8_O_4_ (Xyl)−C_6_H_10_O_4_ (Qui)−C_5_H_8_O_4_ (Xyl)]^+^.

#### 3.3.2. Chitonoidoside A_1_ (**2**)

Colorless powder; [α]${}_{D}^{20}$ −43° (*c* 0.1, 50% MeOH). NMR: See Table S1 and Table 3, Figures S8–S14. (−)HR-ESI-MS *m/z*: 1133.4856 (calc. 1133.4844) [M_Na_−Na]^−^; (+)HR-ESI-MS *m/z*: 1179.4620 (calc. 1179.4628) [M_Na_+Na]^+^; (−)ESI-MS/MS *m/z*: 665.1 [M_Na_−Na–C_30_H_44_O_4_ (Agl)]^−^, 533.1 [M_Na_−Na–C_30_H_46_O_3_ (Agl)−C_5_H_8_O_4_ (Xyl)]^−^, 387.1 [M_Na_−Na–C_30_H_46_O_3_ (Agl)−C_5_H_8_O_4_ (Xyl)−C_6_H_10_O_4_ (Qui)]^−^, 255.0 [M_Na_−Na–C_30_H_46_O_3_ (Agl)−C_5_H_8_O_4_ (Xyl)−C_6_H_10_O_4_ (Qui)−C_5_H_8_O_4_ (Xyl)]^−^.

#### 3.3.3. Chitonoidoside B (**3**)

Colorless powder; [α]${}_{D}^{20}$ −35° (*c* 0.1, 50% MeOH). NMR: See Table S2 and Table 4, Figures S15–S23. (−)HR-ESI-MS *m/z*: 1427.6157 (calc. 1427.6159) [M_Na_−Na]^−^; (+)HR-ESI-MS *m/z*: 1473.5925 (calc. 1473.5943) [M_Na_+Na]^+^; (−)ESI-MS/MS *m/z*: 1281.5 [M_Na_−Na–C_6_H_11_O_4_ (MeXyl)+H]^−^, 1119.5 [M_Na_−Na–C_6_H_11_O_4_ (MeXyl)−C_6_H_10_O_5_ (Glc)+H]^−^; (+)ESI-MS/MS *m/z*: 1354.5 [M_Na_+Na−NaHSO_4_]^+^, 1327.5 [M_Na_+Na−C_6_H_11_O_4_ (MeXyl)+H]^+^, 1194.5 [M_Na_+Na−C_7_H_11_O_8_SNa (MeGlcSO_3_Na)]^+^, 1165.5 [M_Na_+Na−C_6_H_11_O_4_ (MeXyl)−C_6_H_10_O_5_ (Glc)+H]^+^, 1062.5 [M_Na_+Na−C_7_H_11_O_8_SNa (MeGlcSO_3_Na)−C_5_H_8_O_4_ (Xyl)]^+^, 917.5 [M_Na_+Na−C_7_H_11_O_8_SNa (MeGlcSO_3_Na)−C_5_H_8_O_4_ (Xyl)−C_6_H_10_O_4_ (Qui)]^+^.

#### 3.3.4. Chitonoidoside C (**4**)

Colorless powder; [α]${}_{D}^{20}$ −55° (*c* 0.1, 50% MeOH). NMR: See Table S3 and Table 5, Figures S24–S31. (−)HR-ESI-MS *m/z*: 1235.4256 (calc. 1235.4232) [M_2Na_−Na]^−^, 606.2183 (calc. 606.2170) [M_2Na_−2Na]^2^^−^; (−)ESI-MS/MS *m/z*: 1115.5 [M_2Na_−Na–NaHSO_4_]^−^, 1089.4 [M_2Na_−Na−C_6_H_10_O_4_ (Qui)]^−^, 969.4 [M_2Na_−Na−NaHSO_4_–C_6_H_10_O_4_ (Qui)]^−^, 841.4 [M_2Na_−Na−C_6_H_10_O_7_SNa (MeXylOSO_3_)−C_6_H_10_O_4_ (Qui)+H]^−^, 517.1 [M_2Na_−Na–C_30_H_44_O_4_ (Agl)−C_6_H_10_O_7_SNa (MeXylOSO_3_)−H]^−^.

#### 3.3.5. Chitonoidoside D (**5**)

Colorless powder; [α]${}_{D}^{20}$ –59° (*c* 0.1, 50% MeOH). NMR: See Table S4 and Table 6, Figures S32–S39. (−)HR-ESI-MS *m/z*: 1397.4706 (calc. 1397.4760) [M_2Na_−Na]^−^, 687.2412 (calc. 687.2434) [M_2Na_−2Na]^2^^−^; (−)ESI-MS/MS *m/z*: 1277.5 [M_2Na_−Na–NaHSO_4_]^−^, 1119.5 [M_2Na_−Na–C_7_H_11_O_8_SNa (MeGlcOSO_3_)]^−^, 987.4 [M_2Na_−Na–C_7_H_11_O_8_SNa (MeGlcOSO_3_)−C_5_H_8_O_4_ (Xyl)]^−^, 841.3 [M_2Na_−Na–C_7_H_11_O_8_SNa (MeGlcOSO_3_)−C_5_H_8_O_4_ (Xyl)–C_6_H_10_O_4_ (Qui)]^−^.

#### 3.3.6. Chitonoidoside E (**6**)

Colorless powder; [α]${}_{D}^{20}$ –27° (*c* 0.1, 50% MeOH). NMR: See Table S5 and Table 7, Figures S40–S48. (−)HR-ESI-MS *m/z*: 1529.5567 (calc. 1529.5546) [M_2Na_−Na]^−^, 753.2836 (calc. 753.2827) [M_2Na_−2Na]^2^^−^; (−)ESI-MS/MS *m/z*: 1409.5 [M_2Na_−Na–NaHSO_4_]^−^, 1383.5 [M_2Na_−Na–C_6_H_11_O_4_ (MeXyl)+H]^−^, 1263.5 [M_2Na_−Na–C_6_H_11_O_4_ (MeXyl) –NaHSO_4_]^−^, 1251.5 [M_2Na_−Na–C_7_H_11_O_8_SNa (MeGlcOSO_3_)]^−^, 1119.5 [M_2Na_−Na–C_7_H_11_O_8_SNa (MeGlcOSO_3_)−C_5_H_8_O_4_ (Xyl)]^−^, 973.4 [M_2Na_−Na–C_7_H_11_O_8_SNa (MeGlcOSO_3_)−C_5_H_8_O_4_ (Xyl)–C_6_H_10_O_4_ (Qui)]^−^, 827.4 [M_2Na_−Na–C_7_H_11_O_8_SNa (MeGlcOSO_3_)−C_5_H_8_O_4_ (Xyl)–C_6_H_10_O_4_ (Qui) –C_6_H_11_O_4_ (MeXyl)+H]^−^.

### 3.4. Cytotoxic Activity (MTT Assay)

All the compounds (including psolusoside L and cisplatin used as positive control) were tested in concentrations from 0.1 μM to 100 μM using two-fold dilution in distilled H_2_O. The solutions (20 µL) of tested substances in different concentrations and cell suspension (180 µL) were added in wells of 96-well plates (1 × 10^4^ cells/well) and incubated at 37 °C for 24 h in 5% CO_2_. After incubation, the medium with tested substances was replaced by 100 μL of fresh medium. Then, 10 μL of MTT (3-(4,5-dimethylthiazol-2-yl)-2,5-diphenyltetrazolium bromide) (Sigma-Aldrich, St. Louis, MO, USA) stock solution (5 mg/mL) was added to each well, and the microplate was incubated for 4 h. After that, 100 μL of SDS-HCl solution (1 g SDS/10 mL distilled H_2_O/17 μL 6 M HCl) was added to each well followed by incubation for 18 h. The absorbance of the converted dye formazan was measured using a Multiskan FC microplate photometer (Thermo Fisher Scientific, Waltham, MA, USA) at a wavelength of 570 nm. Cytotoxic activity of the substances was calculated as the concentration that caused 50% cell activity inhibition (IC_50_). All the experiments were made in triplicate, *p* < 0.05.

### 3.5. Cytotoxic Activity (MTS Assay)

HL-60 cells (6 × 10^3^/200 μL) were seeded in 96-well plates at 37 °C for 24 h in a 5% CO_2_ incubator. The cells were treated with compounds **1**–**5** at concentrations ranging from 0 to 100 μM for an additional 24 h. Subsequently, cells were incubated with 10 μL MTS ([3-(4,5-dimethylthiazol-2-yl)-5-(3-carboxymethoxyphenyl)-2-(4-sulfophenyl)-2H-tetrazolium) reagent for 4 h, and the absorbance in each well was measured at 490/630 nm using plate reader PHERAstar FS (BMG Labtech, Ortenberg, Germany). All the experiments were repeated three times, and the mean absorbance values were calculated. The results are expressed as the percentage of inhibition that produced a reduction in absorbance by the compound’s treatment compared to the non-treated cells (control). All the experiments were made in triplicate, *p* < 0.01.

### 3.6. Hemolytic Activity

Erythrocytes were isolated from the blood of albino CD-1 mice (18–20 g) and from human blood by centrifugation with phosphate-buffered saline (PBS) (pH 7.4) at 4 °C for 5 min by 450 g on centrifuge LABOFUGE 400R (Heraeus, Hanau, Germany) three times. Then, the residue of erythrocytes was resuspended in ice cold phosphate saline buffer (pH 7.4) to a final optical density of 1.5 at 700 nm, and kept on ice. For the hemolytic assay, 180 µL of erythrocyte suspension was mixed with 20 µL of test compound solution (including psolusoside L used as positive control) in V-bottom 96-well plates. After 1 h of incubation at 37 °C, plates were exposed to centrifugation 10 min at 900 g on laboratory centrifuge LMC-3000 (Biosan, Riga, Latvia). Then, 100 µL of supernatant was carefully selected and transferred in new flat-plates, respectively. Lysis of erythrocytes was determined by measuring of the concentration of hemoglobin in the supernatant with microplate photometer Multiskan FC (Thermo Fisher Scientific, Waltham, MA, USA), λ = 570 nm. The effective dose causing 50% hemolysis of erythrocytes (ED_50_) was calculated using the computer program SigmaPlot 10.0. All the experiments were made in triplicate, *p* < 0.01.

## 4. Conclusions

To summarize, six unknown earlier triterpene glycosides were isolated from the sea cucumber *Psolus chitonoides*. Some interesting structural features were discovered. Three of them contain a new non-holostane aglycone lacking a lactone and having an 18(20)-epoxy cycle. The second finding is 3-*O*-methylxylose residue in the glycosides of the sea cucumber belonging to genus *Psolus*. Finally, the sulfation of 3-*O*-methylxylose by C-4, found in isolated compounds, has never been revealed before in the holothuroid compounds. Five new types of the carbohydrate chains (chitonoidosides of the groups A–E) were found and structurally elucidated. The architecture of tetrasaccharide branched by C-4 Xyl1 carbohydrate chain of chitonoidoside C (**4**) was also uncommon when compared with the other glycosides from the animals of *Psolus* genus. Thus, a number of newly found sulfated hexaosides continue to grow since monosulfated hexaosides were isolated first from *Cladolabes schmeltzii* [25]. After that finding, di- and trisulfated compounds with hexasaccharide chains were found in *T. kurilensis* [9] and in *P. chitonoides* (chitonoidoside E (**6**) having two sulfate groups). We were surprised that the replacement of 18(20)-lactone for 18(20)-epoxide in the aglycones does not significantly affect the cytotoxic activities of isolated glycosides.

## Figures and Tables

**Table 1:** ^13^C and ^1^H NMR chemical shifts, HMBC, and ROESY correlations of carbohydrate moiety of chitonoidoside A (**1**) *.

Atom	δ_C_ mult. *^a,b,c^*	δ_H_ mult. (*J* in Hz) *^d^*	HMBC	ROESY
Xyl1 (1→C-3)				
1	105.4 CH	4.82 d (7.5)	C: 3	H-3; H-3, 5 Xyl1
2	**83.9** CH	4.08 t (7.5)	C: 1 Qui2	H-1 Qui2
3	78.0 CH	4.20 t (8.6)	C: 2, 4 Xyl1	H-1 Xyl1
4	70.7 CH	4.18 m		
5	66.5 CH_2_	4.32 dd (4.3; 11.7)	C: 1, 3, 4, Xyl1	
		3.67 t (10.5)		
Qui2 (1→2Xyl1)				
1	105.5 CH	5.19 d (7.2)	C: 2 Xyl1	H-2 Xyl1; H-3, 5 Qui2
2	76.5 CH	4.07 t (8.0)	C: 1, 3 Qui2	H-4 Qui2
3	74.9 CH	4.15 t (8.0)	C: 2, 4 Qui2	H-1, 5 Qui2
4	**85.4** CH	3.69 t (8.0)	C: 3, 5 Qui2, 1 Xyl3	H-1 Xyl3
5	71.6 CH	3.84 m		H-1, 3 Qui2
6	17.9 CH_3_	1.76 d (4.8)	C: 4, 5 Qui2	H-4 Qui2
Xyl3 (1→4Qui2)				
1	104.2 CH	4.89 d (8.6)	C: 4 Qui2	H-4 Qui2; H-3 Xyl3
2	73.5 CH	3.98 t (8.6)	C: 1, 3 Xyl3	
3	**86.5** CH	4.17 t (8.6)	C: 2, 4 Xyl3; 1 MeGlc4	H-1 MeGlc4; H-1 Xyl3
4	68.6 CH	4.08 m		
5	66.3 CH_2_	4.23 dd (6.4; 12.8)	C: 1, 3, 4 Xyl3	
		3.65 t (10.7)		H-1 Xyl3
MeGlc4 (1→3Xyl3)				
1	104.6 CH	5.30 d (8.6)	C: 3 Xyl3	H-3 Xyl3; H-3, 5 MeGlc4
2	74.0 CH	3.83 t (8.6)	C: 1, 3 MeGlc4	H-4 MeGlc4
3	87.2 CH	3.62 t (8.6)	C: 2, 4 MeGlc4, OMe	H-1, 5 MeGlc4; OMe
4	69.9 CH	4.11 t (8.6)	C: 3, 5 MeGlc4	H-2, 6 MeGlc4
5	76.2 CH	4.01 m		H-1, 3 MeGlc4
6	*66.6* CH_2_	5.03 d (11.8)	C: 4 MeGlc4	
		4.93 dd (6.4; 11.8)		H-4 MeGlc4
OMe	60.4 CH_3_	3.78 s	C: 3 MeGlc4	

*^a^* Recorded at 176.03 MHz in C_5_D_5_N/D_2_O (4/1). *^b^* Bold = interglycosidic positions. *^c^* Italic = sulphate position. *^d^* Recorded at 700.00 MHz in C_5_D_5_N/D_2_O (4/1). Multiplicity by 1D TOCSY. * The chemical shifts and 2D NMR data for carbohydrate part of chitonoidosides A (**1**) and A_1_ (**2**) are very similar but have minor differences. The original spectra of **2** and the assigned signals are provided in Table S1, Figures S8–S10.

**Table 2:** ^13^C and ^1^H NMR chemical shifts, HMBC, and ROESY correlations of the aglycone moiety of chitonoidoside A (**1**) *.

Position	δ_C_ mult. *^a^*	δ_H_ mult. (*J* in Hz) *^b^*	HMBC	ROESY
1	36.0 CH_2_	1.74 m β		H-11, H-19
		1.48 m α		H-3, H-11
2	27.0 CH_2_	2.33 brdd (13.5) α		
		2.00 brd (13.5) β		H-19, H-30
3	88.6 CH	3.30 dd (3.7; 11.6)	C: 4, 30, 31, C:1 Xyl1	H-5, H-31, H1-Xyl1
4	39.8 C			
5	52.7 CH	0.94 brd (12.6)	C: 6, 10, 30	H-1, H-3, H-7, H-31
6	21.0 CH_2_	1.71 m α		H-31
		1.47 m β		H-8, H-19, H-30
7	28.8 CH_2_	1.59 m β		H-15
		1.20 m α		H-5, H-32
8	40.9 CH	2.39 m	C: 9	H-6, H-18, H-19
9	151.0 C			
10	39.5 C			
11	114.7 CH	5.38 d (6.1)	C: 8, 10, 12, 13	H-1
12	33.8 CH_2_	2.37 m α	C: 9, 11, 13, 18	H-32
		2.27 dd (5.4; 16.9) β	C: 9, 11, 13, 14, 18	H-18, H-21
13	56.3 C			
14	40.3 C			
15	50.4 CH_2_	2.50 d (15.6)	C: 14, 16, 32	H-18
		2.17 d (15.6)	C: 13, 16, 17, 32	H-7, H-32
16	215.9 C			
17	63.9 CH	2.38 s	C: 12, 16, 18, 20, 21	H-21, H-22, H-32
18	73.8 CH_2_	4.08 d (9.8)	C: 12, 14, 20	H-8, H-15, H-22 (weak)
		3.71 d (9.8)	C: 12, 14, 17, 20	H-12, H-19
19	22.8 CH_3_	1.09 s	C: 1, 5, 9, 10	H-1, H-2, H-6, H-8, H-18
20	86.2 C			
21	26.1 CH_3_	1.32 s	C: 17, 20, 22	H-12, H-17, H-18, H-22
22	37.9 CH_2_	1.83 m		H-21
		1.67 m		H-21
23	23.2 CH_2_	1.82 m		
		1.68 m		
24	38.3 CH_2_	2.07 m	C: 22, 23, 25	H-26
25	145.9 C			
26	110.0 CH_2_	4.83 brs	C: 24, 27	H-21, H-24
		4.81 brs	C: 24, 27	H-27
27	22.2 CH_3_	1.74 s	C: 24, 25, 26	H-26
30	16.5 CH_3_	1.15 s	C: 3, 4, 5, 31	H-2, H-6, H-31
31	27.9 CH_3_	1.34 s	C: 3, 4, 5, 30	H-3, H-5, H-6, H-30, H-1 Xyl1
32	21.4 CH_3_	0.85 s	C: 8, 13, 14, 15	H-7, H-15, H-17

*^a^* Recorded at 176.03 MHz in C_5_D_5_N/D_2_O (4/1). *^b^* Recorded at 700.00 MHz in C_5_D_5_N/D_2_O (4/1). * The NMR data for aglycone parts of chitonoidoside A (**1**), B (**3**) and E (**6**) are similar but with minor differences. The original spectra are presented in Figures S1–S6, S15–S20, and S40–S45, correspondingly, and the assigned signals for the aglycone parts of **3** and **6** are provided as Tables S2 and S5.

**Table 3:** ^13^C and ^1^H NMR chemical shifts, HMBC, and ROESY correlations of the aglycone moiety of chitonoidoside A_1_ (**2**) *.

Position	δ_C_ mult. *^a^*	δ_H_ mult. (*J* in Hz) *^b^*	HMBC	ROESY
1	36.8 CH_2_	1.74 m		H-11, H-19
		1.34 m		H-3, H-5, H-11
2	27.5 CH_2_	2.12 m		
		1.88 m		H-19, H-30
3	89.6 CH	3.17 dd (4.1; 11.7)	C: 4, 30, 31, C:1 Xyl1	H-1, H-5, H-31, H1-Xyl1
4	40.2 C			
5	53.5 CH	0.82 brd (12.2)	C: 6, 10, 19, 30	H-1, H-3, H-31
6	21.6 CH_2_	1.61 m		H-31
		1.42 m		H-19, H-30
7	29.0 CH_2_	1.59 m		
		1.16 m		H-32
8	39.4 CH	3.14 m		H-6, H-19
9	151.7 C			
10	40.4 C			
11	111.8 CH	5.27 d (4.6)	C: 8, 10, 12, 13	H-1
12	32.7 CH_2_	2.62 d (16.8)	C: 9, 11, 13, 18	H-17, H-32
		2.46 dd (5.1; 16.8)	C: 9, 11, 13, 14, 18	
13	56.5 C			
14	42.7 C			
15	52.7 CH_2_	2.40 d (15.8)	C: 13, 16, 17, 32	H-7, H-32
		2.12 d (15.8)	C: 8, 14, 16, 32	
16	215.2 C			
17	61.9 CH	2.85 s	C: 12, 13, 16, 18, 20, 21	H-21, H-32
18	177.4 C			
19	22.6 CH_3_	1.28 s	C: 1, 5, 9, 10	H-1, H-2, H-8, H-30
20	84.3 C			
21	27.4 CH_3_	1.43 s	C: 17, 20, 22	H-12, H-17
22	38.9 CH_2_	1.72 m	C: 20, 21, 23	
		1.54 m	C: 17, 20, 21, 23	
23	22.8 CH_2_	1.70 m		
		1.44 m		
24	38.5 CH_2_	1.89 m	C: 22, 25, 26, 27	H-21
25	146.4 C			
26	111.2 CH_2_	4.70 brs	C: 24, 27	
		4.68 brs	C: 24, 27	
27	22.8 CH_3_	1.62 s	C: 24, 25, 26	
30	17.2 CH_3_	1.00 s	C: 3, 4, 5, 31	H-31
31	28.7 CH_3_	1.18 s	C: 3, 4, 5, 30	H-3, H-5, H-30
32	21.3 CH_3_	0.88 s	C: 8, 13, 14, 15	H-7, H-12, H-17

*^a^* Recorded at 176.03 MHz in C_5_D_5_N/D_2_O (4/1). *^b^* Recorded at 700.00 MHz in C_5_D_5_N/D_2_O (4/1). * The NMR data for aglycone parts of chitonoidoside A_1_ (**2**), C (**4**), and D (**5**) are similar but with minor differences. The original spectra are presented in Figures S8–S13, S24–S29, and S32–S37, correspondingly, and the assigned signals for the aglycone parts of **4** and **5** are provided as Tables S3 and S4.

**Table 4:** ^13^C and ^1^H NMR chemical shifts, HMBC and ROESY correlations of carbohydrate moiety of chitonoidoside B (**3**).

Atom	δ_C_ mult. *^a b,c^*	δ_H_ mult. (*J* in Hz) *^d^*	HMBC	ROESY
Xyl1 (1→C-3)				
1	104.8 CH	4.67 d (7.6)	C: 3	H-3; H-3, 5 Xyl1
2	**82.5** CH	3.96 t (8.2)	C: 1 Qui2; 1 Xyl1	H-1 Qui2
3	75.2 CH	4.17 t (8.2)	C: 2, 4 Xyl1	
4	**77.5** CH	4.24 m		H-1 Glc5
5	63.5 CH_2_	4.40 dd (6.0; 12.5)	C: 3 Xyl1	
		3.65 dd (9.2; 12.5)		H-1 Xyl1
Qui2 (1→2Xyl1)				
1	104.7 CH	5.02 d (8.7)	C: 2 Xyl1	H-2 Xyl1; H-3, 5 Qui2
2	75.7 CH	3.87 t (9.0)	C: 3 Qui2	H-4 Qui2
3	74.9 CH	3.94 t (9.0)		
4	**85.6** CH	3.50 t (9.0)	C: 3 Qui2, 1 Xyl3	H-1 Xyl3; H-2 Qui2
5	71.4 CH	3.66 dd (6.2; 9.0)		H-1 Qui2
6	17.8 CH_3_	1.63 d (6.2)	C: 4, 5 Qui2	H-4 Qui2
Xyl3 (1→4Qui2)				
1	104.4 CH	4.77 d (7.7)	C: 4 Qui2	H-4 Qui2; H-3, 5 Xyl3
2	73.2 CH	3.85 t (7.7)	C: 1, 3 Xyl3	
3	**87.0** CH	4.06 t (8.6)	C: 2 Xyl3; 1 MeGlc4	H-1 MeGlc4; H-1 Xyl3
4	68.7 CH	3.91 t (8.6)		
5	65.7 CH_2_	4.12 dd (5.7; 11.5)	C: 3, 4 Xyl3	
6		3.60 t (11.5)		H-1 Xyl3
MeGlc4 (1→3Xyl3)	104.5 CH	5.12 d (7.7)	C: 3 Xyl3	H-3 Xyl3; H-3, 5 MeGlc4
1	74.3 CH	3.80 t (7.7)	C: 1 MeGlc4	
2	86.4 CH	3.64 t (9.7)	C: 2, 4 MeGlc4, OMe	H-1, 5 MeGlc4
3	69.9 CH	3.96 t (9.7)	C: 3 MeGlc4	H-2, 6 MeGlc4
4	75.5 CH	4.04 m		H-1, 3 MeGlc4
5	*67.1* CH_2_	4.98 d (11.6)		
6		4.72 dd (5.8; 11.6)	C: 5 MeGlc4	
	60.5 CH_3_	3.76 s	C: 3 MeGlc4	
Glc5 (1→4Xyl1)				
1	102.2 CH	4.93 d (7.9)	C: 4 Xyl1	H-4 Xyl1; H-3, 5 Glc5
2	73.3 CH	3.92 t (7.9)	C: 1, 3 Glc5	
3	**86.8** CH	4.09 t (7.9)	C: 4 Glc5; 1 MeXyl6	H-1 MeXyl6; H-1,5 Glc5
4	69.2 CH	3.86 t (7.9)	C: 3, 5 Glc5	H-6 Glc5
5	77.3 CH	3.86 t (7.9)		H-1, 3 Glc5
6	61.7 CH_2_	4.32 brd (11.4)		
		4.05 dd (4.8; 11.4)		
MeXyl6 (1→3Glc5)				
1	105.3 CH	5.09 d (7.3)	C: 3 Glc5	H-3 Glc5; H-3, 5 MeXyl6
2	74.2 CH	3.80 t (8.4)	C: 1 MeXyl6	
3	86.7 CH	3.58 t (8.4)	C: 2, 4 MeXyl6; OMe	H-1 MeXyl6; OMe
4	69.6 CH	3.97 m		
5	66.4 CH_2_	4.14 dd (5.5; 11.7)	C: 1, 3, 4 MeXyl6	
		3.60 t (11.0)		H-1 MeXyl6
OMe	60.5 CH_3_	3.79 s	C: 3 MeXyl6	H-3 MeXyl6

*^a^* Recorded at 176.04 MHz in C_5_D_5_N/D_2_O (4/1). *^b^* Bold = interglycosidic positions. *^c^* Italic = sulfate position. *^d^* Recorded at 700.13 MHz in C_5_D_5_N/D_2_O (4/1). Multiplicity by 1D TOCSY.

**Table 5:** ^13^C and ^1^H NMR chemical shifts, HMBC, and ROESY correlations of carbohydrate moiety of chitonoidoside C (**4**).

Atom	δ_C_ mult. *^a,b,c^*	δ_H_ mult. (*J* in Hz) *^d^*	HMBC	ROESY
Xyl1 (1→C-3)				
1	104.8 CH	4.68 d (6.6)	C: 3	H-3; H-3, 5 Xyl1
2	**82.1** CH	3.98 t (6.6)	C: 1 Qui2; 1, 3 Xyl1	H-1 Qui2
3	75.1 CH	4.17 t (7.4)	C: 4 Xyl1	H-1, 5 Xyl1
4	**77.9** CH	4.18 t (7.4)		H-1 Glc3
5	63.5 CH_2_	4.39 dd (4.5; 11.6)	C: 3 Xyl1	
		3.64 m		H-1 Xyl1
Qui2 (1→2Xyl1)				
1	104.9 CH	5.06 d (7.5)	C: 2 Xyl1	H-2 Xyl1; H-3, 5 Qui2
2	76.3 CH	3.89 t (8.6)	C: 1, 3 Qui2	H-4 Qui2
3	76.8 CH	4.07 t (8.6)	C: 4 Qui2	H-1, 5 Qui2
4	76.2 CH	3.59 t (8.6)	C: 3 Qui2	H-2 Qui2
5	72.8 CH	3.70 dd (6.3; 8.7)		H-1 Qui2
6	18.2 CH_3_	1.53 d (6.3)	C: 4, 5 Qui2	H-4 Qui2
Glc3 (1→4Xyl1)				
1	102.2 CH	4.90 d (8.2)	C: 4 Xyl1	H-4 Xyl1; H-3 Glc3
2	73.3 CH	3.82 t (8.2)	C: 1, 3 Glc3	
3	**85.8** CH	4.06 t (8.2)	C: 2, 4 Glc3; 1 MeXyl4	H-1 MeXyl4
4	68.8 CH	3.86 t (8.9)	C: 3 Glc3	
5	75.3 CH	4.02 m		H-1 Glc3
6	*67.2* CH_2_	4.96 d (11.6)		
		4.70 dd (5.6; 11.6)		
MeXyl4 (1→3Glc3)				
1	104.8 CH	5.08 d (7.5)	C: 3 Glc3	H-3 Glc3; H-3, 5 MeXyl4
2	73.7 CH	3.82 t (7.5)	C: 1 MeXyl4	
3	84.1 CH	3.65 t (8.8)	C: 2, 4 MeXyl4, OMe	
4	*75.5* CH	4.93 m	C: 3 MeXyl4	
5	64.5 CH_2_	4.88 dd (5.2; 11.7)	C: 3, 4 MeXyl4	
		3.74 t (10.2)		H-4 MeXyl4
OMe	60.1 CH_3_	3.81 s	C: 3 MeXyl4	

*^a^* Recorded at 176.04 MHz in C_5_D_5_N/D_2_O (4/1). *^b^* Bold = interglycosidic positions. *^c^* Italic = sulfate position. *^d^* Recorded at 700.13 MHz in C_5_D_5_N/D_2_O (4/1). Multiplicity by 1D TOCSY.

**Table 6:** ^13^C and ^1^H NMR chemical shifts, HMBC, and ROESY correlations of carbohydrate moiety of chitonoidoside D (**5**).

Atom	δ_C_ mult. *^a,b,c^*	δ_H_ mult. (*J* in Hz) *^d^*	HMBC	ROESY
Xyl1 (1→C-3)				
1	104.8 CH	4.67 d (7.1)	C: 3	H-3; H-3, 5 Xyl1
2	**82.1** CH	3.97 t (7.6)	C: 1 Qui2; 1 Xyl1	H-1 Qui2
3	75.2 CH	4.16 t (8.6)	C: 2, 4 Xyl1	H-1, 5 Xyl1
4	**77.6** CH	4.15 m		H-1 Glc5
5	63.6 CH_2_	4.38 dd (3.5; 11.2)	C: 3 Xyl1	
		3.65 m		H-1 Xyl1
Qui2 (1→2Xyl1)				
1	104.5 CH	5.04 d (7.0)	C: 2 Xyl1	H-2 Xyl1; H-3, 5 Qui2
2	75.7 CH	3.86 t (9.0)	C: 3 Qui2	H-4 Qui2
3	74.9 CH	3.96 t (9.0)		
4	**85.7**	3.48 t (9.0)	C: 3 Qui2, 1 Xyl3	H-1 Xyl3; H-2 Qui2
5	71.5 CH	3.68 dd (5.6; 9.0)		H-1 Qui2
6	17.8 CH_3_	1.61 d (6.2)	C: 4, 5 Qui2	
Xyl3 (1→4Qui2)				
1	104.4 CH	4.74 d (8.1)	C: 4 Qui2	H-4 Qui2; H-3, 5 Xyl3
2	73.2 CH	3.84 t (8.9)	C: 1, 3 Xyl3	
3	**87.0** CH	4.04 t (8.9)	C: 2 Xyl3; 1 MeGlc4	H-1 MeGlc4
4	68.8 CH	3.88 m		
5	65.7 CH_2_	4.12 dd (5.7; 12.2)	C: 3, 4 Xyl3	
		3.60 d (11.4)		H-1 Xyl3
MeGlc4 (1→3Xyl3)				
1	104.6 CH	5.11 d (7.3)	C: 3 Xyl3	H-3 Xyl3; H-3, 5 MeGlc4
2	74.3 CH	3.79 t (8.9)	C: 1 MeGlc4	H-4 MeGlc4
3	86.4 CH	3.64 t (8.9)	C: 2, 4 MeGlc4, OMe	
4	69.9 CH	3.94 t (8.9)	C: 3 MeGlc4	
5	75.5 CH	4.03 m		
6	*67.2* CH_2_	4.95 brd (9.8)		
		4.69 dd (5.7; 11.4)	C: 5 MeGlc4	
OMe	60.6 CH_3_	3.76 s	C: 3 MeGlc4	
Glc5 (1→4Xyl1)				
1	102.7 CH	4.85 d (8.1)	C: 4 Xyl1	H-4 Xyl1; H-3, 5 Glc5
2	73.8 CH	3.81 t (8.9)	C: 1, 3 Glc5	H-4 Glc5
3	76.8 CH	4.09 t (8.9)	C: 4 Glc5; 1 MeXyl6	H-1 Glc5
4	70.6 CH	4.00 m	C: 3, 5 Glc5	
5	75.8 CH	4.00 m		H-1 Glc5
6	*67.4* CH_2_	4.95 d (11.4)		
		4.73 dd (4.9; 11.4)		

*^a^* Recorded at 176.04 MHz in C_5_D_5_N/D_2_O (4/1). *^b^* Bold = interglycosidic positions. *^c^* Italic = sulfate position. *^d^* Recorded at 700.13 MHz in C_5_D_5_N/D_2_O (4/1). Multiplicity by 1D TOCSY.

**Table 7:** ^13^C and ^1^H NMR chemical shifts, HMBC and ROESY correlations of carbohydrate moiety of chitonoidoside E (**6**).

Atom	δ_C_ mult. *^a,b,c^*	δ_H_ mult. (*J* in Hz) *^d^*	HMBC	ROESY
Xyl1 (1→C-3)				
1	104.8 CH	4.67 d (6.9)	C: 3	H-3; H-3, 5 Xyl1
2	**82.1** CH	3.97 t (6.9)	C: 1 Qui2; 3 Xyl1	H-1 Qui2
3	75.2 CH	4.17 m	C: 4 Xyl1	H-1, 5 Xyl1
4	**77.8** CH	4.17 m		H-1 Glc5; H-2 Xyl1
5	63.5 CH_2_	4.38 dd (5.5; 11.5)	C: 3 Xyl1	
		3.64 m		H-1 Xyl1
Qui2 (1→2Xyl1)				
1	104.5 CH	5.05 d (7.8)	C: 2 Xyl1	H-2 Xyl1; H-3, 5 Qui2
2	75.7 CH	3.86 t (9.1)	C: 1, 3 Qui2	H-4 Qui2
3	74.8 CH	3.97 t (9.1)	C: 2 Qui2	H-1 Qui2
4	**85.6** CH	3.49 t (9.1)	C: 1 Xyl3; 3, 5 Qui2	H-1 Xyl3; H-2 Qui2
5	71.4 CH	3.68 dd (5.8; 9.1)		H-1 Qui2
6	17.8 CH_3_	1.62 d (5.8)	C: 4, 5 Qui2	H-4 Qui2
Xyl3 (1→4Qui2)				
1	104.4 CH	4.75 d (7.6)	C: 4 Qui2	H-4 Qui2; H-3, 5 Xyl3
2	73.2 CH	3.84 t (8.4)	C: 1 Xyl3	
3	**87.0** CH	4.04 t (8.4)	C: 1 MeGlc4; 2, 4 Xyl3	H-1 MeGlc4
4	68.8 CH	3.91 m		
5	65.7 CH_2_	4.12 dd (5.9; 11.0)	C: 4 Xyl3	
6		3.59 t (11.0)	C: 1, 3 Xyl3	H-1 Xyl3
				
MeGlc4 (1→3Xyl3)	104.6 CH	5.12 d (7.6)	C: 3 Xyl3	H-3 Xyl3; H-3, 5 MeGlc4
1	74.2 CH	3.80 t (8.4)	C: 1 MeGlc4	
2	86.4 CH	3.64 t (8.4)	C: 2, 4 MeGlc4, OMe	H-1, 5 MeGlc4
3	69.9 CH	3.95 t (8.4)	C: 6 MeGlc4	
4	75.3 CH	4.03 m		H-1, 3 MeGlc4
5	*67.1* CH_2_	4.96 d (11.0)	C: 5 MeGlc4	
6		4.71 dd (5.9; 11.8)	C: 5 MeGlc4	
	60.5 CH_3_	3.76 s	C: 3 MeGlc4	
Glc5 (1→4Xyl1)				
1	102.2 CH	4.90 d (7.6)	C: 4 Xyl1	H-4 Xyl1; H-3, 5 Glc5
2	73.2 CH	3.84 t (9.3)	C: 1, 3 Glc5	
3	**86.0** CH	4.06 t (9.3)	C: 1 MeXyl6; 2 Glc5	H-1 MeXyl6; H-1 Glc5
4	68.9 CH	3.88 t (9.3)		H-6 Glc5
5	75.5 CH	4.02 m		H-1 Glc5
6	67.1 CH_2_	4.95 d (10.1)	C: 4, 5 Glc5	
		4.70 dd (5.9; 11.8)		
MeXyl6 (1→3Glc5)				
1	105.2 CH	5.07 d (7.6)	C: 3 Glc5	H-3 Glc5; H-3, 5 MeXyl6
2	74.3 CH	3.79 t (8.4)	C: 1 MeXyl6	
3	86.8 CH	3.57 t (8.4)	C: 2, 4 MeXyl6; OMe	H-1 MeXyl6; OMe
4	69.7 CH	3.97 m	C: 3, 5 MeXyl6	
5	66.4 CH_2_	4.14 dd (5.1; 11.8)	C: 1, 3, 4 MeXyl6	
		3.60 t (11.0)	C: 1 MeXyl6	H-1 MeXyl6
OMe	60.5 CH_3_	3.79 s	C: 3 MeXyl6	H-3 MeXyl6

*^a^* Recorded at 176.04 MHz in C_5_D_5_N/D_2_O (4/1). *^b^* Bold = interglycosidic positions. *^c^* Italic = sulfate position. *^d^* Recorded at 700.13 MHz in C_5_D_5_N/D_2_O (4/1). Multiplicity by 1D TOCSY.

**Table 8:** The cytotoxic activities of glycosides **1**–**5** and psolusoside L and cysplatin (positive control) against mouse and human erythrocytes, HeLa, DLD-1, and HL-60 human cell lines.

Glycoside	ED_50_, µM, Erythrocytes		Cytotoxicity EC_50_, µM		
	Mouse	Human	HeLa	DLD-1	HL-60
chitonoidoside A (**1**)	1.51 ± 0.17	1.41 ± 0.01	36.20 ± 0.20	25.32 ± 0.17	8.09 ± 0.35
chitonoidoside A_1_ (**2**)	1.87 ± 0.17	2.22 ± 0.05	36.26 ± 0.74	34.51 ± 0.62	7.03 ± 0.27
chitonoidoside B (**3**)	2.08 ± 0.09	2.32 ± 0.05	11.24 ± 0.05	39.91 ± 0.32	10.43 ± 0.13
chitonoidoside C (**4**)	1.65 ± 0.13	2.28 ± 0.08	72.09 ± 0.75	65.96 ± 0.14	76.95 ± 0.39
chitonoidoside D (**5**)	1.12 ± 0.09	1.69 ± 0.25	15.94 ± 0.31	6.80 ± 0.31	1.86 ± 0.23
psolusoside L	0.63 ± 0.12	0.23 ± 0.01	17.54 ± 1.08	9.94 ± 0.07	3.69 ± 0.49
cisplatin			4.41 ± 0.31	5.13 ± 0.12	11.20 ± 0.12

## Supplementary Materials

The original spectral data (Figures S1–S48 and Tables S1–S5) are available online at https://www.mdpi.com/article/10.3390/md19080449/s1.
