# Supplementary material for: Unusual Structures and Cytotoxicities of Chitonoidosides A, A1, B, C, D, and E, Six Triterpene Glycosides from the Far Eastern Sea Cucumber Psolus chitonoides
Source: Mar Drugs. 2021 Aug 5;19(8):449. doi: 10.3390/md19080449 (PMC8398228; doi:10.3390/md19080449)
Supplement: Supplementary file 1 [file marinedrugs-19-00449-s001.zip › marinedrugs-1319069-SI.pdf]

Supplementary data content page

Title: **Unusual Structures and Cytotoxicities of Chitonoidosides A, A$_1$, B, C, D and E, Six Triterpene Glycosides from the Far Eastern Sea Cucumber *Psolus chitonoides***

Authors: Alexandra S. Silchenko*[1], Anatoly I. Kalinovsky[1], Sergey A. Avilov[1], Pelageya V. Andrijaschenko[1], Roman S. Popov[1], Ekaterina A. Chingizova[1], Vladimir I. Kalinin[1] and Stonik V.A. [1]

Address: [1]*G.B. Elyakov Pacific Institute of Bioorganic Chemistry, Far Eastern Branch of Russian Academy of Sciences, Pr. 100-let Vladivostoku 159, 690022 Vladivostok, Russia*

Correspondence: sialexandra@mail.ru; Tel.: +7-423-231-1168

Contents:

Figure S1. The ^{13}C NMR (176.03 MHz) spectrum of chitonoidoside A (**1**) in C_5D_5N/D_2O (4/1)

Figure S2. The ^{1}H NMR (700.00 MHz) spectrum of chitonoidoside A (**1**) in C_5D_5N/D_2O (4/1)

Figure S3. The COSY (700.00 MHz) spectrum of chitonoidoside A (**1**) in C_5D_5N/D_2O (4/1)

Figure S4. The HSQC (700.00 MHz) spectrum of chitonoidoside A (**1**) in C_5D_5N/D_2O (4/1)

Figure S5. The ROESY (700.00 MHz) spectrum of chitonoidoside A (**1**) in C_5D_5N/D_2O (4/1)

Figure S6. The HMBC (700.00 MHz) spectrum of chitonoidoside A (**1**) in C_5D_5N/D_2O (4/1)

Figure S7. HR-ESI-MS and ESI-MS/MS spectra of chitonoidoside A (**1**)

Table S1. ^{13}C and ^{1}H NMR chemical shifts, HMBC and ROESY correlations of carbohydrate moiety of chitonoidoside A$_1$ (**2**)

Figure S8. The ^{13}C NMR (176.03 MHz) spectrum of chitonoidoside A$_1$ (**2**) in C_5D_5N/D_2O (4/1)

Figure S9. The ^{1}H NMR (700.00 MHz) spectrum of chitonoidoside A$_1$ (**2**) in C_5D_5N/D_2O (4/1)

Figure S10. The COSY (700.00 MHz) spectrum of chitonoidoside A$_1$ (**2**) in C_5D_5N/D_2O (4/1)

Figure S11. The HSQC (700.00 MHz) spectrum of chitonoidoside A$_1$ (**2**) in C_5D_5N/D_2O (4/1)

Figure S12. The HMBC (700.00 MHz) spectrum of chitonoidoside A$_1$ (**2**) in C_5D_5N/D_2O (4/1)

Figure S13. The ROESY (700.00 MHz) spectrum of chitonoidoside A$_1$ (**2**) in C_5D_5N/D_2O (4/1)

Figure S14. HR-ESI-MS and ESI-MS/MS spectra of chitonoidoside A_1 (**2**)

Table S2. ^{13}C and ^{1}H NMR chemical shifts, HMBC and ROESY correlations of the aglycone part of chitonoidoside B (**3**)

Figure S15. The ^{13}C NMR (176.03 MHz) spectrum of chitonoidoside B (**3**) in C_5D_5N/D_2O (4/1)

Figure S16. The ^{1}H NMR (700.00 MHz) spectrum of chitonoidoside B (**3**) in C_5D_5N/D_2O (4/1)

Figure S17. The COSY (700.00 MHz) spectrum of chitonoidoside B (**3**) in C_5D_5N/D_2O (4/1)

Figure S18. The HSQC (700.00 MHz) spectrum of chitonoidoside B (**3**) in C_5D_5N/D_2O (4/1)

Figure S19. The HMBC (700.00 MHz) spectrum of chitonoidoside B (**3**) in C_5D_5N/D_2O (4/1)

Figure S20. The ROESY (700.00 MHz) spectrum of chitonoidoside B (**3**) in C_5D_5N/D_2O (4/1)

Figure S21. 1 D TOCSY (700.00 MHz) spectra of Xyl1, Qui2 and Xyl3 of chitonoidoside B (**3**) in C_5D_5N/D_2O (4/1)

Figure S22. 1 D TOCSY (700.00 MHz) spectra of MeGlc4, Glc5 and MeXyl6 of chitonoidoside B (**3**) in C_5D_5N/D_2O (4/1)

Figure S23. HR-ESI-MS and ESI-MS/MS spectra of chitonoidoside B (**3**)

Table S3. ^{13}C and ^{1}H NMR chemical shifts, HMBC and ROESY correlations of the aglycone part of chitonoidoside C (**4**)

Figure S24. The ^{13}C NMR (176.03 MHz) spectrum of chitonoidoside C (**4**) in C_5D_5N/D_2O (4/1)

Figure S25. The ^{1}H NMR (700.00 MHz) spectrum of chitonoidoside C (**4**) in C_5D_5N/D_2O (4/1)

Figure S26. The COSY (700.00 MHz) spectrum of chitonoidoside C (**4**) in C_5D_5N/D_2O (4/1)

Figure S27. The HSQC (700.00 MHz) spectrum of chitonoidoside C (**4**) in C_5D_5N/D_2O (4/1)

Figure S28. The ROESY (700.00 MHz) spectrum of chitonoidoside C (**4**) in C_5D_5N/D_2O (4/1)

Figure S29. The HMBC (700.00 MHz) spectrum of chitonoidoside C (**4**) in C_5D_5N/D_2O (4/1)

Figure S30. 1 D TOCSY (700.00 MHz) spectra of chitonoidoside C (**4**) in C_5D_5N/D_2O (4/1)

Figure S31. HR-ESI-MS and ESI-MS/MS spectra of chitonoidoside C (**4**)

Table S4. ^{13}C and ^{1}H NMR chemical shifts, HMBC and ROESY correlations of the aglycone part of chitonoidoside D (**5**)

Figure S32. The ^{13}C NMR (176.03 MHz) spectrum of chitonoidoside D (**5**) in C_5D_5N/D_2O (4/1)

Figure S33. The ^{1}H NMR (700.00 MHz) spectrum of chitonoidoside D (**5**) in C_5D_5N/D_2O (4/1)

Figure S34. The COSY (700.00 MHz) spectrum of chitonoidoside D (**5**) in C_5D_5N/D_2O (4/1)

Figure S35. The HSQC (700.00 MHz) spectrum of chitonoidoside D (**5**) in C_5D_5N/D_2O (4/1)

Figure S36. The HMBC (700.00 MHz) spectrum of chitonoidoside D (**5**) in C_5D_5N/D_2O (4/1)

Figure S37. The ROESY (700.00 MHz) spectrum of chitonoidoside D (**5**) in C_5D_5N/D_2O (4/1)

Figure S38. 1D TOCSY (700.00 MHz) spectra of chitonoidoside D (**5**) in C_5D_5N/D_2O (4/1)

Figure S39. HR-ESI-MS and ESI-MS/MS spectra of chitonoidoside D (**5**)

Table S5. ^{13}C and ^{1}H NMR chemical shifts, HMBC and ROESY correlations of the aglycone part of chitonoidoside E (**6**)

Figure S40. The ^{13}C NMR (176.03 MHz) spectrum of chitonoidoside E (**6**) in C_5D_5N/D_2O (4/1)

Figure S41. The ^{1}H NMR (700.00 MHz) spectrum of chitonoidoside E (**6**) in C_5D_5N/D_2O (4/1)

Figure S42. The COSY (700.00 MHz) spectrum of chitonoidoside E (**6**) in C_5D_5N/D_2O (4/1)

Figure S43. The HSQC (700.00 MHz) spectrum of chitonoidoside E (**6**) in C_5D_5N/D_2O (4/1)

Figure S44. The ROESY (700.00 MHz) spectrum of chitonoidoside E (**6**) in C_5D_5N/D_2O (4/1)

Figure S45. The HMBC (700.00 MHz) spectrum of chitonoidoside E (**6**) in C_5D_5N/D_2O (4/1)

Figure S46. 1D TOCSY (700.00 MHz) spectra of Xyl1, Qui2 and Xyl3 of chitonoidoside E (**6**) in C_5D_5N/D_2O (4/1)

Figure S47. 1D TOCSY (700.00 MHz) spectra of MeGlc4, Glc5 and MeXyl6 of chitonoidoside E (**6**) in C_5D_5N/D_2O (4/1)

Figure S48. HR-ESI-MS and ESI-MS/MS spectra of chitonoidoside E (**6**)

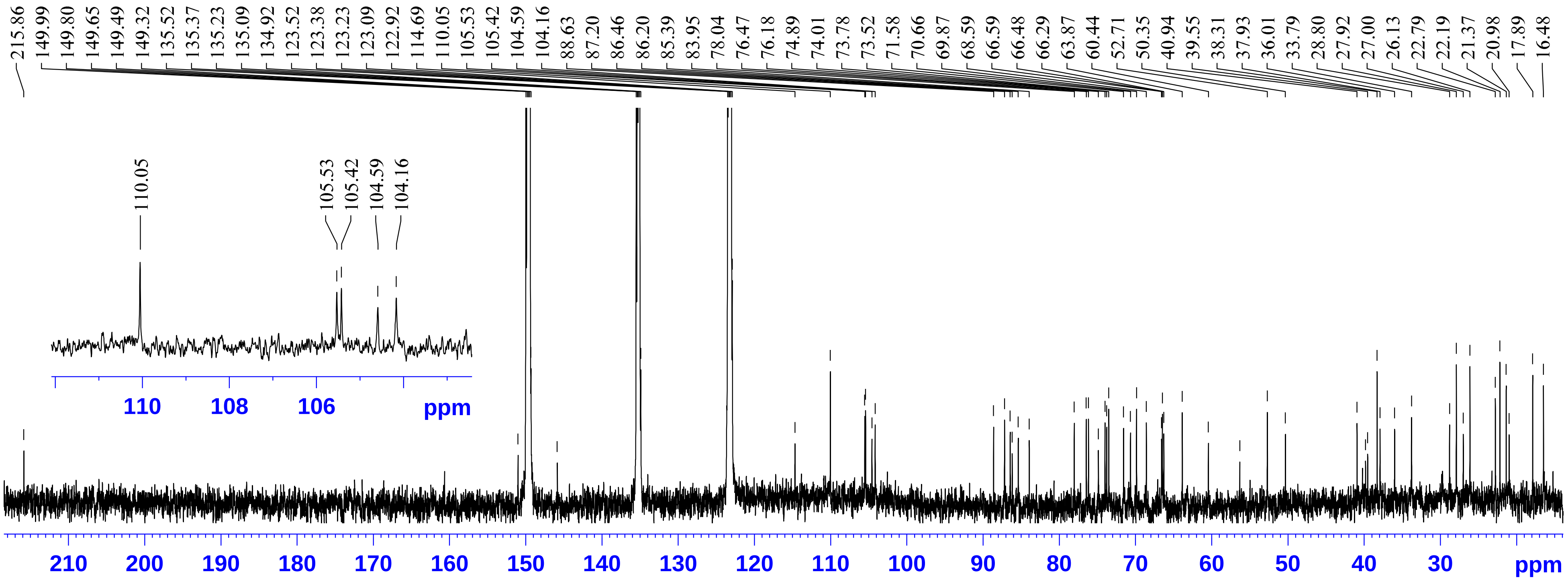

Figure S1. The ^{13}C NMR (176.03 MHz) spectrum of chitonoidoside A (**1**) in C_5D_5N/D_2O (4/1)

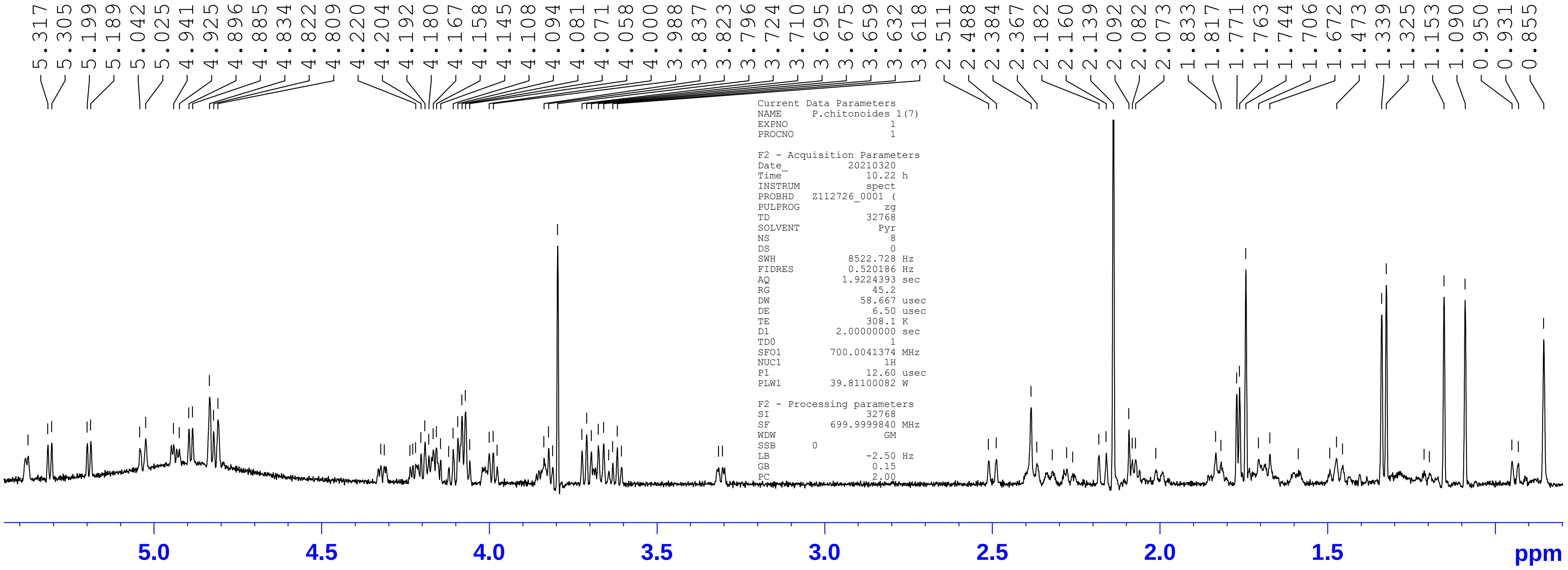

Figure S2. The ^{1}H NMR (700.00 MHz) spectrum of chitonoidoside A (**1**) in C_5D_5N/D_2O (4/1)

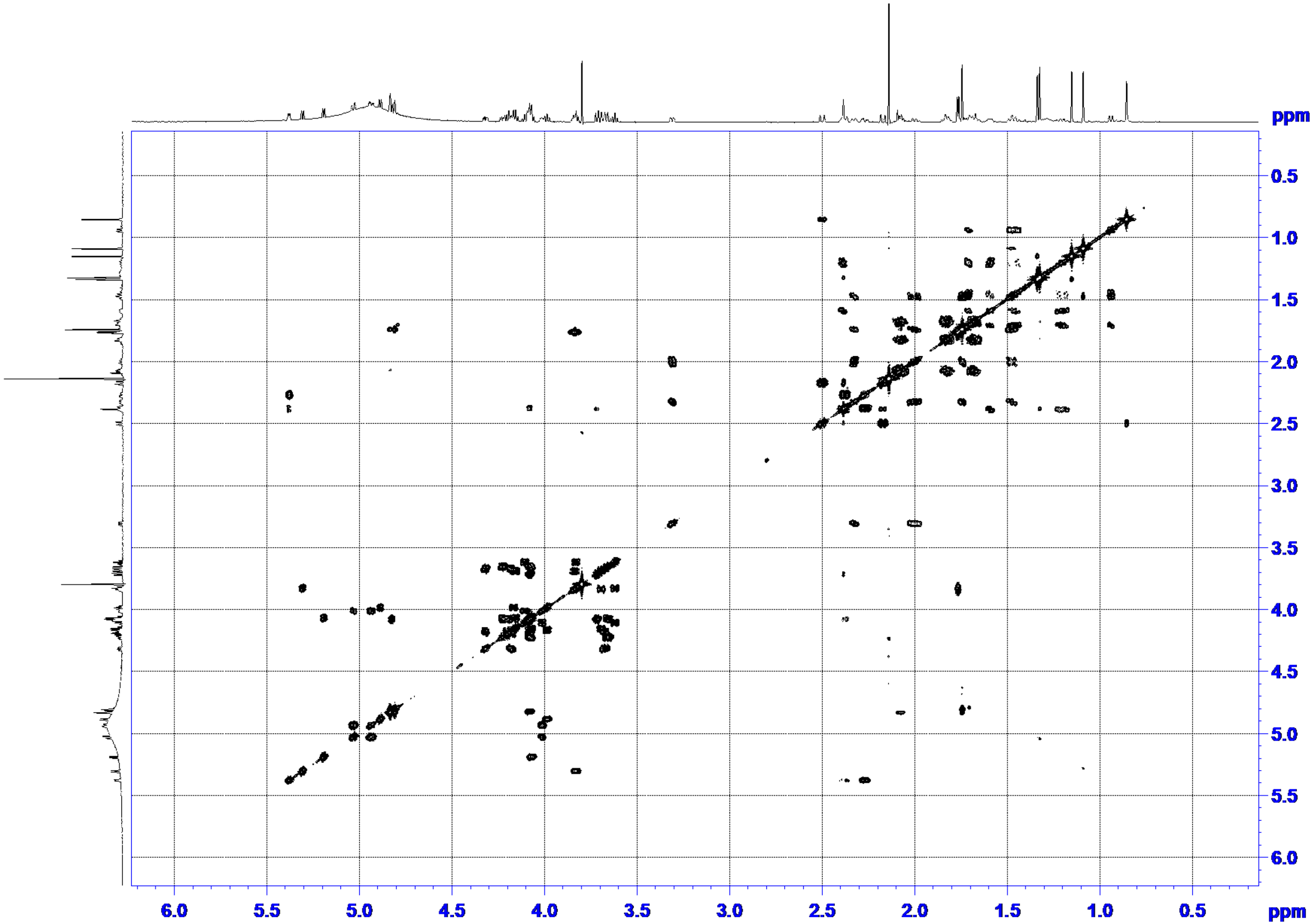

Figure S3. The COSY (700.00 MHz) spectrum of chitonoidoside A (**1**) in C_5D_5N/D_2O (4/1)

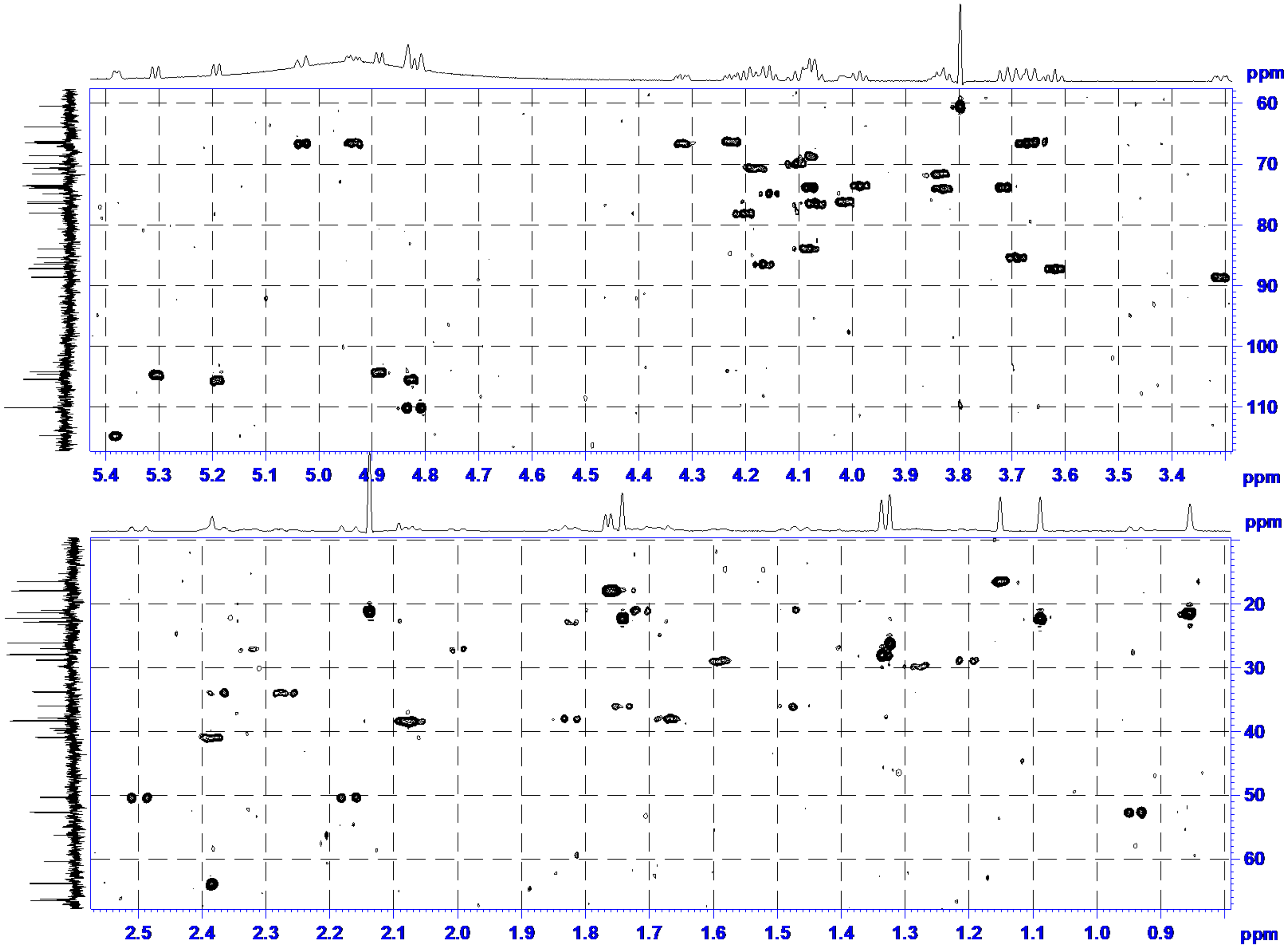

Figure S4. The HSQC (700.00 MHz) spectrum of chitonoidoside A (**1**) in C_5D_5N/D_2O (4/1)

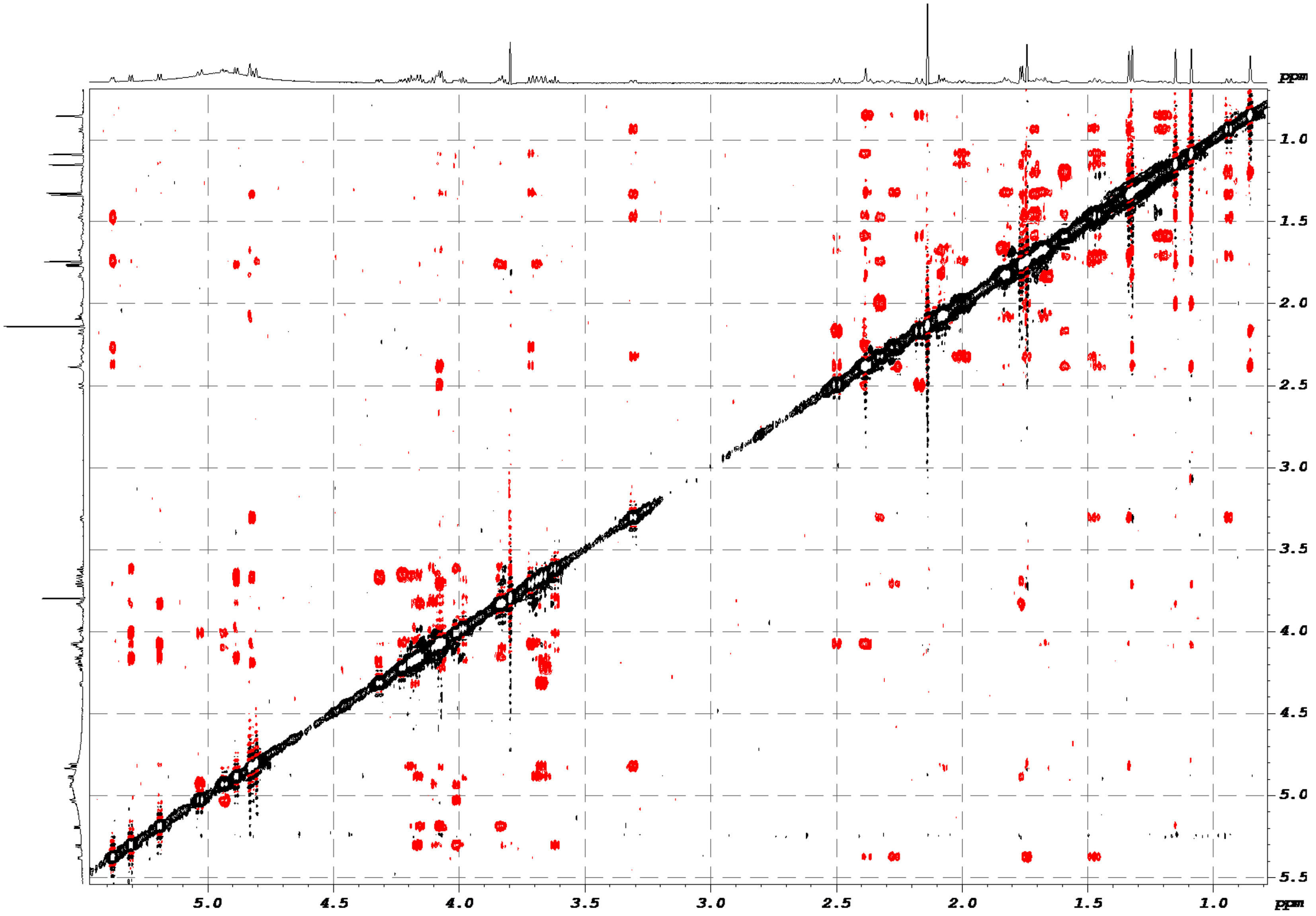

Figure S5. The ROESY (700.00 MHz) spectrum of chitonoidoside A (**1**) in C_5D_5N/D_2O (4/1)

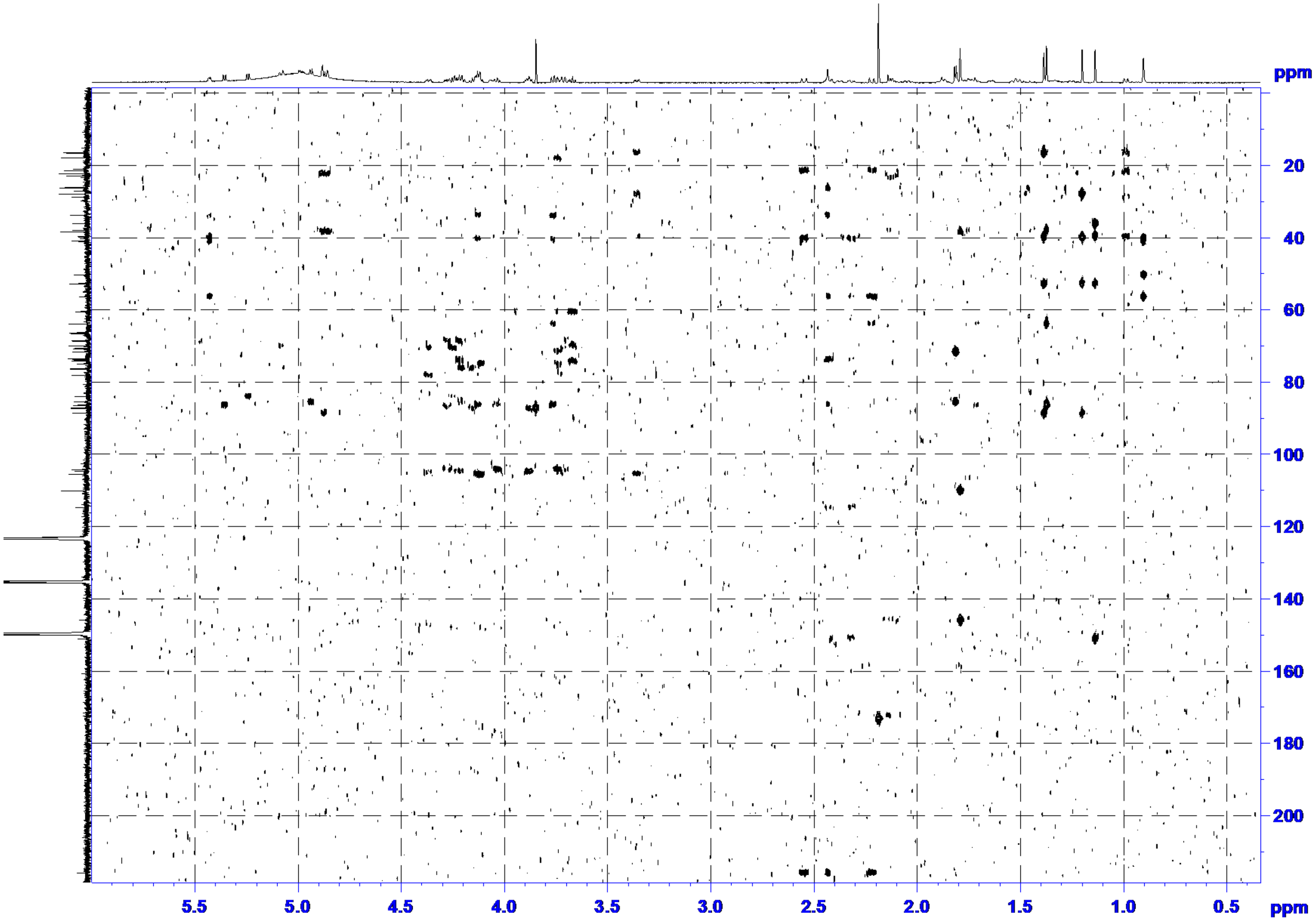

Figure S6. The HMBC (700.00 MHz) spectrum of chitonoidoside A (**1**) in C_5D_5N/D_2O (4/1)

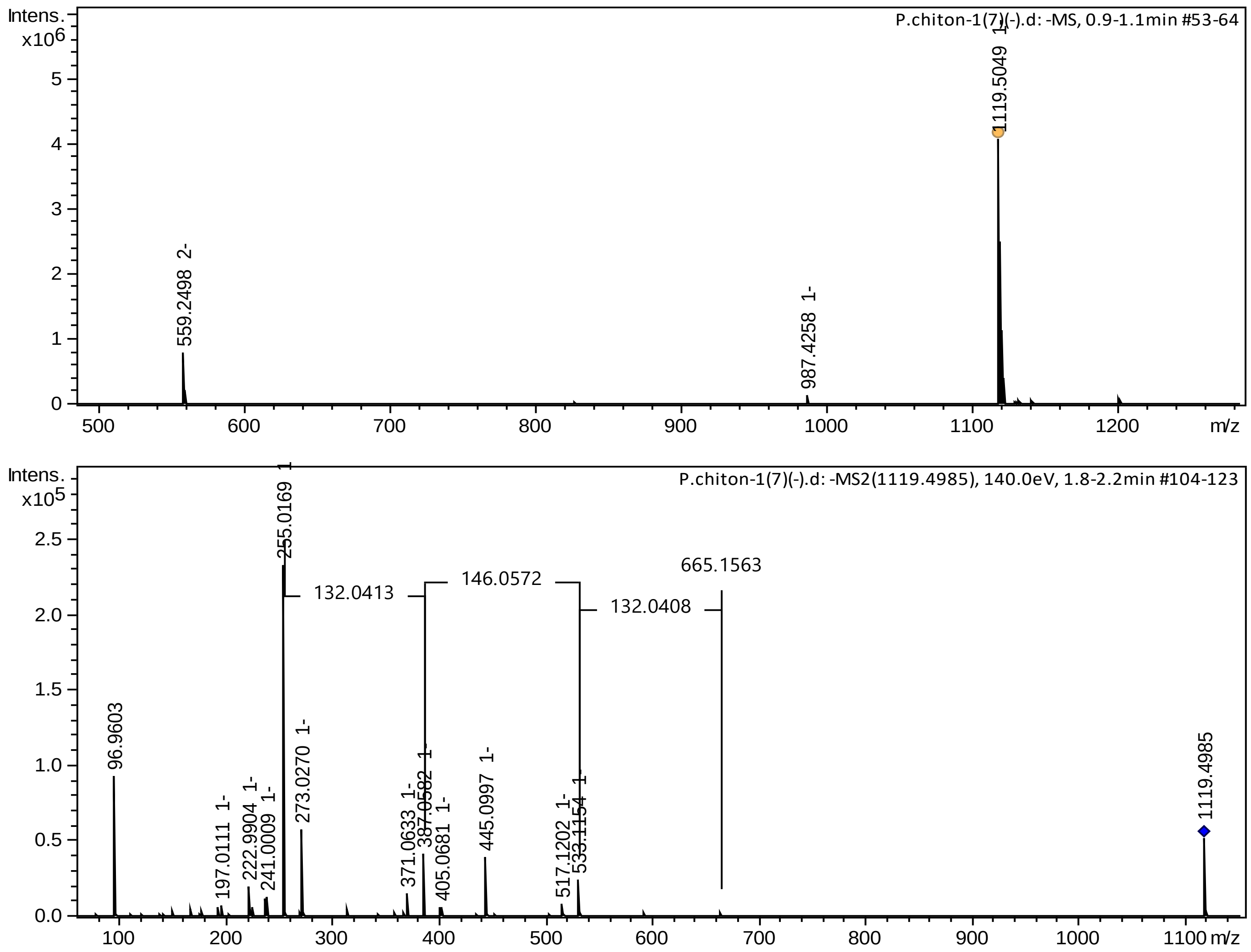

Figure S7. HR-ESI-MS (–) and ESI-MS/MS (–) spectra of chitonoidoside A (**1**)

Table S1. ^{13}C and ^{1}H NMR chemical shifts, HMBC and ROESY correlations of carbohydrate moiety of chitonoidoside A$_1$ (**2**).

Atom	δ_C mult.[a, b, c]	δ_H mult. (J in Hz)[d]	HMBC	ROESY
Xyl1 (1→C-3)				
1	105.8 CH	4.71 d (7.6)	C: 3	H-3; H-3, 5 Xyl1
2	**83.7** CH	3.95 t (7.6)	C: 1 Qui2, 1, 3 Xyl1	H-1 Qui2
3	77.9 CH	4.15 t (8.9)	C: 2, 4 Xyl1	H-1 Xyl1
4	70.9 CH	4.10 m	C: 3, 5 Xyl1	
5	66.7 CH_2	4.25 dd (5.0; 11.0)	C: 1, 3, 4, Xyl1	
		3.63 t (9.9; 11.0)	C: 1, 3 Xyl1	H-1 Xyl1
Qui2 (1→2Xyl1)				
1	105.5 CH	5.03 d (7.3)	C: 2 Xyl1	H-2 Xyl1; H-3, 5 Qui2
2	76.6 CH	3.91 t (8.8)	C: 1, 3 Qui2	
3	75.7 CH	3.96 t (8.8)	C: 2, 4 Qui2	H-5 Qui2
4	**86.4** CH	3.51 t (8.8)	C: 3, 5, 6 Qui2, 1 Xyl3	H-1 Xyl3, H-6 Qui2
5	72.2 CH	3.67 dd (5.9; 8.8)	C: 4 Qui2	H-1, 3 Qui2
6	18.5 CH_3	1.63 d (5.9)	C: 4, 5 Qui2	
Xyl3 (1→4Qui2)				
1	105.1 CH	4.78 d (7.3)	C: 4 Qui2	H-4 Qui2; H-3, 5 Xyl3
2	73.9 CH	3.85 t (9.1)	C: 1, 3 Xyl3	
3	**87.8** CH	4.06 t (9.1)	C: 2, 4 Xyl3; 1 MeGlc4	H-1 MeGlc4; H-1 Xyl3
4	69.5 CH	3.91 m	C: 1, 3, 5 Xyl3	
5	66.5 CH_2	4.12 dd (6.4; 11.9)	C: 3, 4 Xyl3	
		3.60 t (11.0)	C: 1, 3, 4 Xyl3	H-1 Xyl3
MeGlc4 (1→3Xyl3)				
1	105.3 CH	5.12 d (7.6)	C: 3 Xyl3	H-3 Xyl3; H-3, 5 MeGlc4
2	75.0 CH	3.80 t (7.6)	C: 1, 3 MeGlc4	
3	87.2 CH	3.64 t (7.6)	C: 2, 4 MeGlc4, OMe	H-1, 5 MeGlc4; OMe
4	70.7 CH	3.96 t (8.7)	C: 3, 5, 6 MeGlc4	
5	76.3 CH	4.04 t (7.6)	C: 4 MeGlc4	H-1, 3 MeGlc4
6	*67.8* CH_2	4.97 d (10.9)	C: 4, 5 MeGlc4	
		4.72 dd (5.6; 11.5)	C: 5 MeGlc4	
OMe	61.2 CH_3	3.76 s	C: 3 MeGlc4	

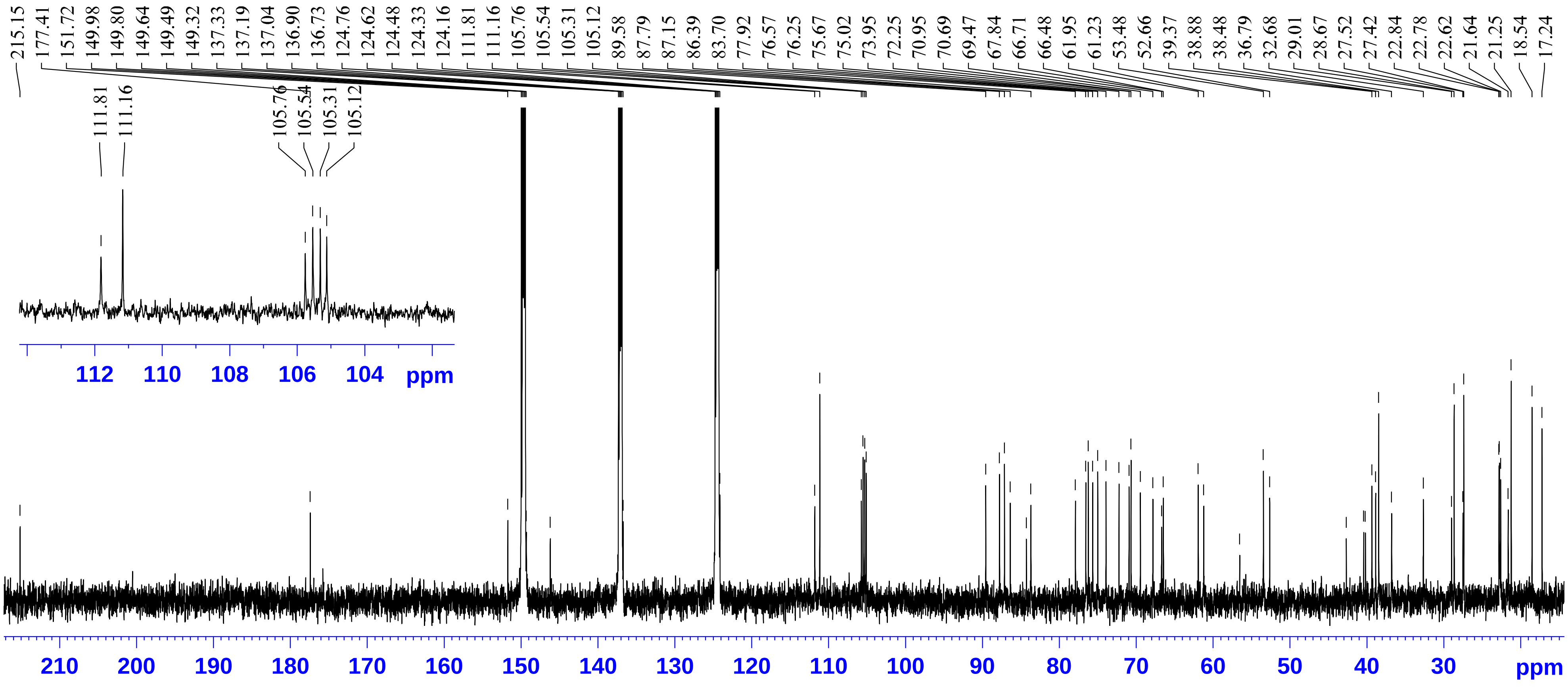

Figure S8. The ^{13}C NMR (176.03 MHz) spectrum of chitonoidoside A$_1$ (**2**) in C_5D_5N/D_2O (4/1)

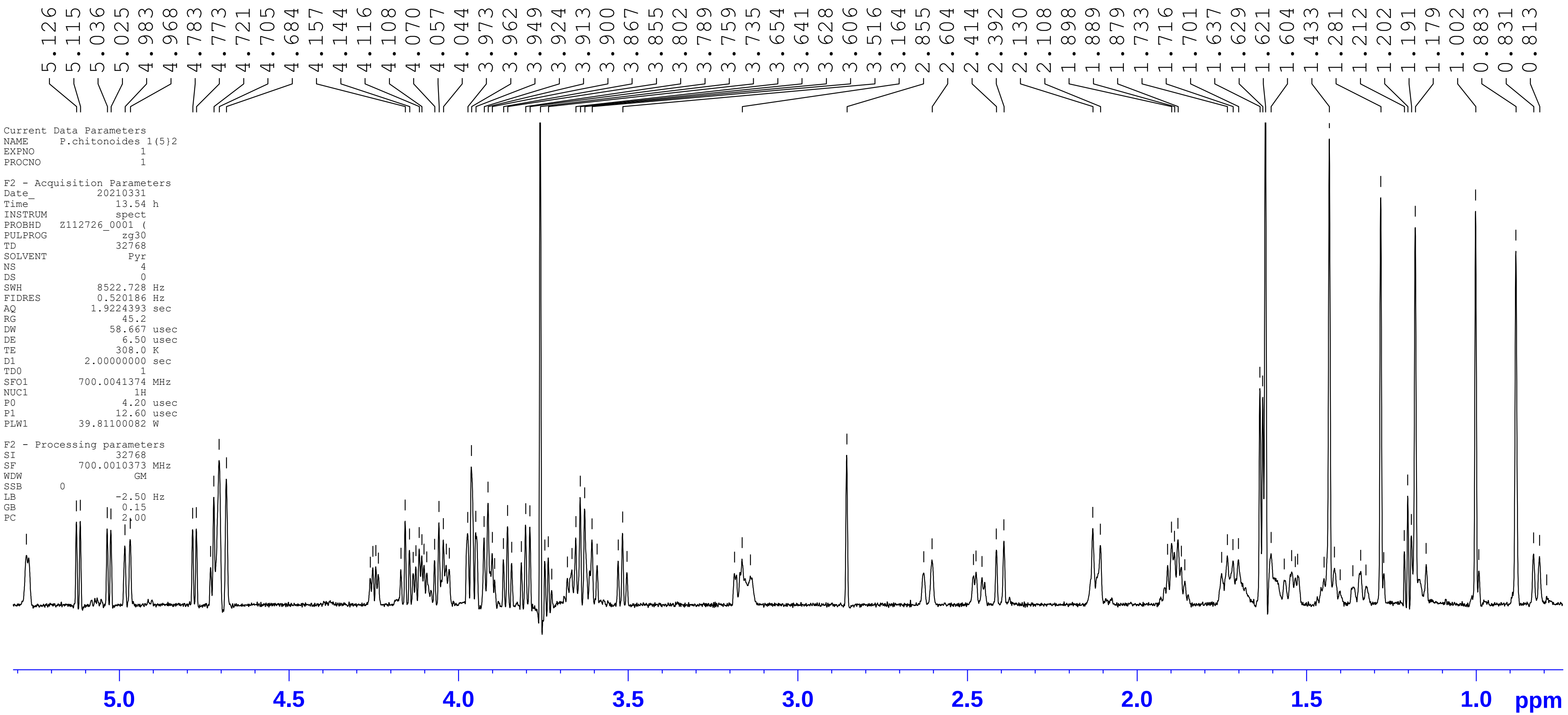

Figure S9. The ^{1}H NMR (700.00 MHz) spectrum of chitonoidoside A$_1$ (**2**) in C_5D_5N/D_2O (4/1)

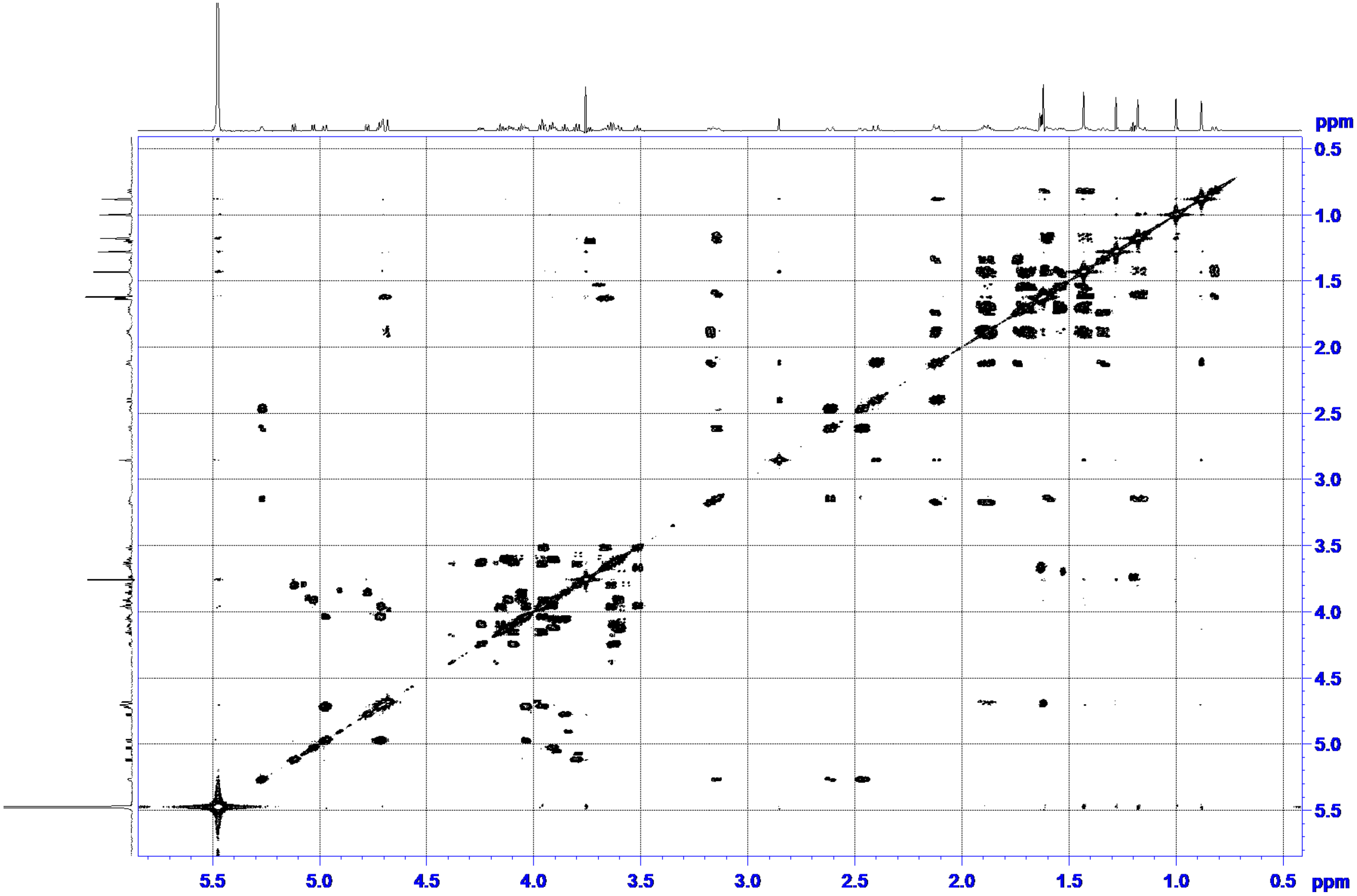

Figure S10. The COSY (700.00 MHz) spectrum of chitonoidoside A1 (**2**) in C_5D_5N/D_2O (4/1)

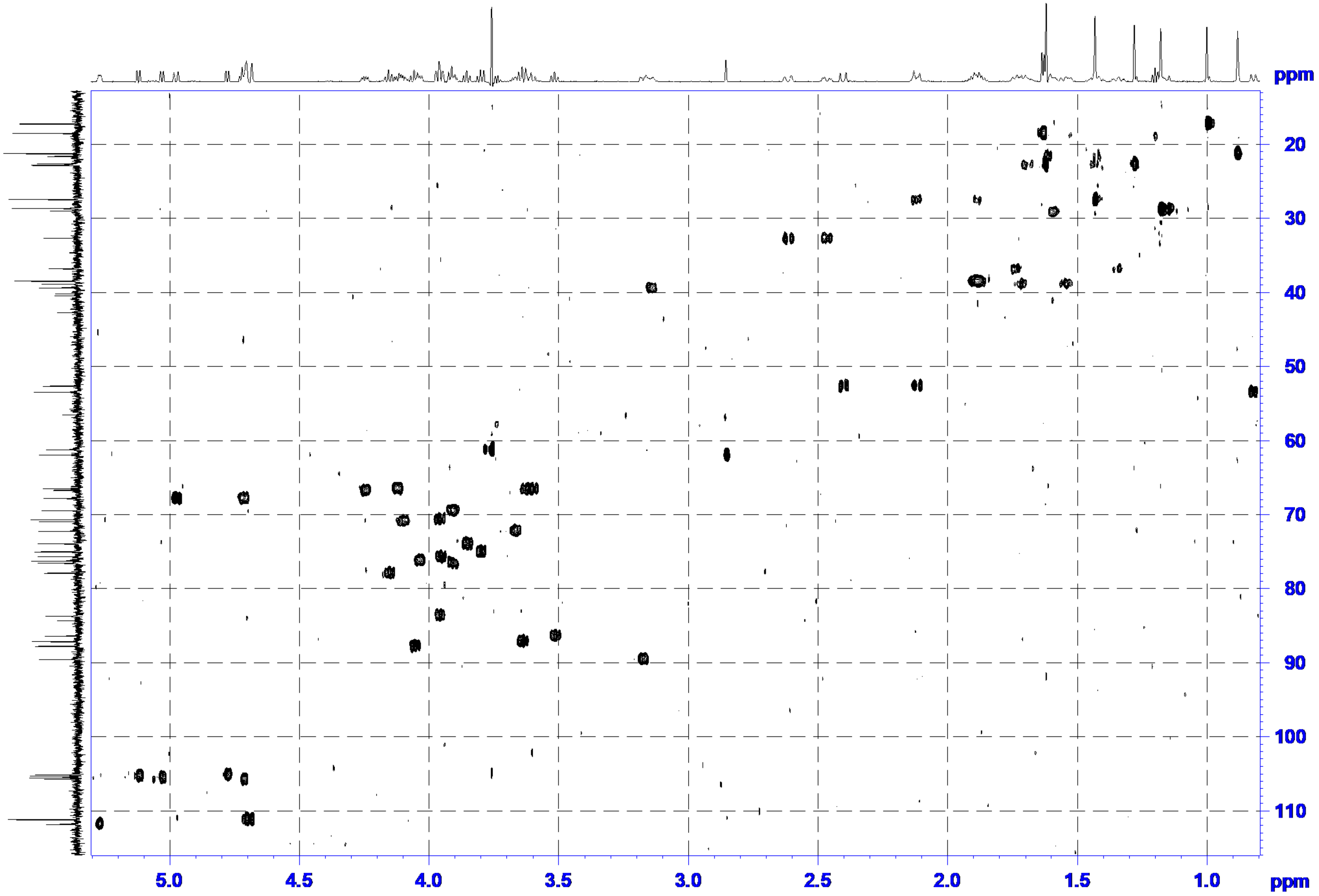

Figure S11. The HSQC (700.00 MHz) spectrum of chitonoidoside A_1 (**2**) in C_5D_5N/D_2O (4/1)

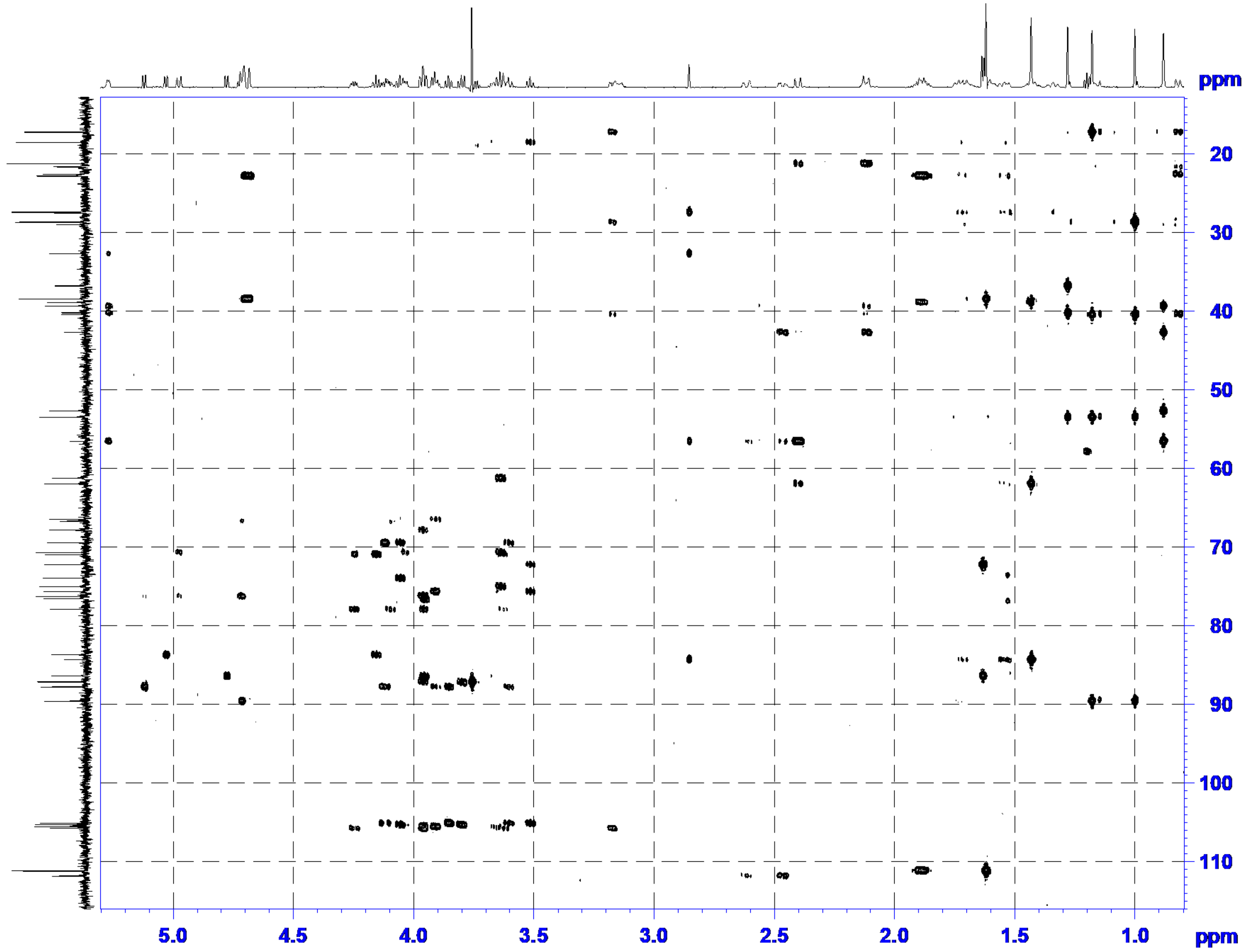

Figure S12. The HMBC (700.00 MHz) spectrum of chitonoidoside A1 (**2**) in C_5D_5N/D_2O (4/1)

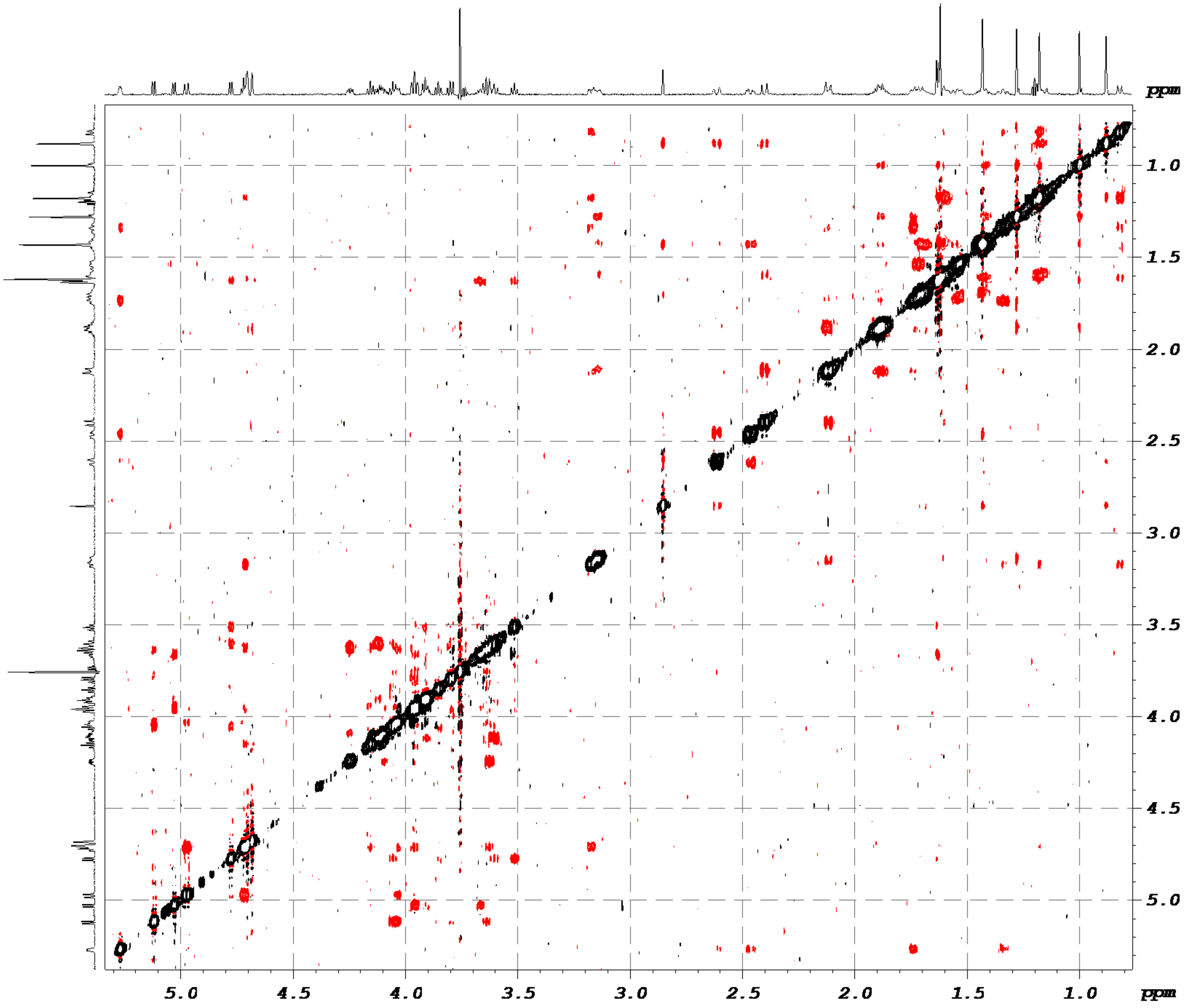

Figure S13. The ROESY (700.00 MHz) spectrum of chitonoidoside A1 (**2**) in C_5D_5N/D_2O (4/1)

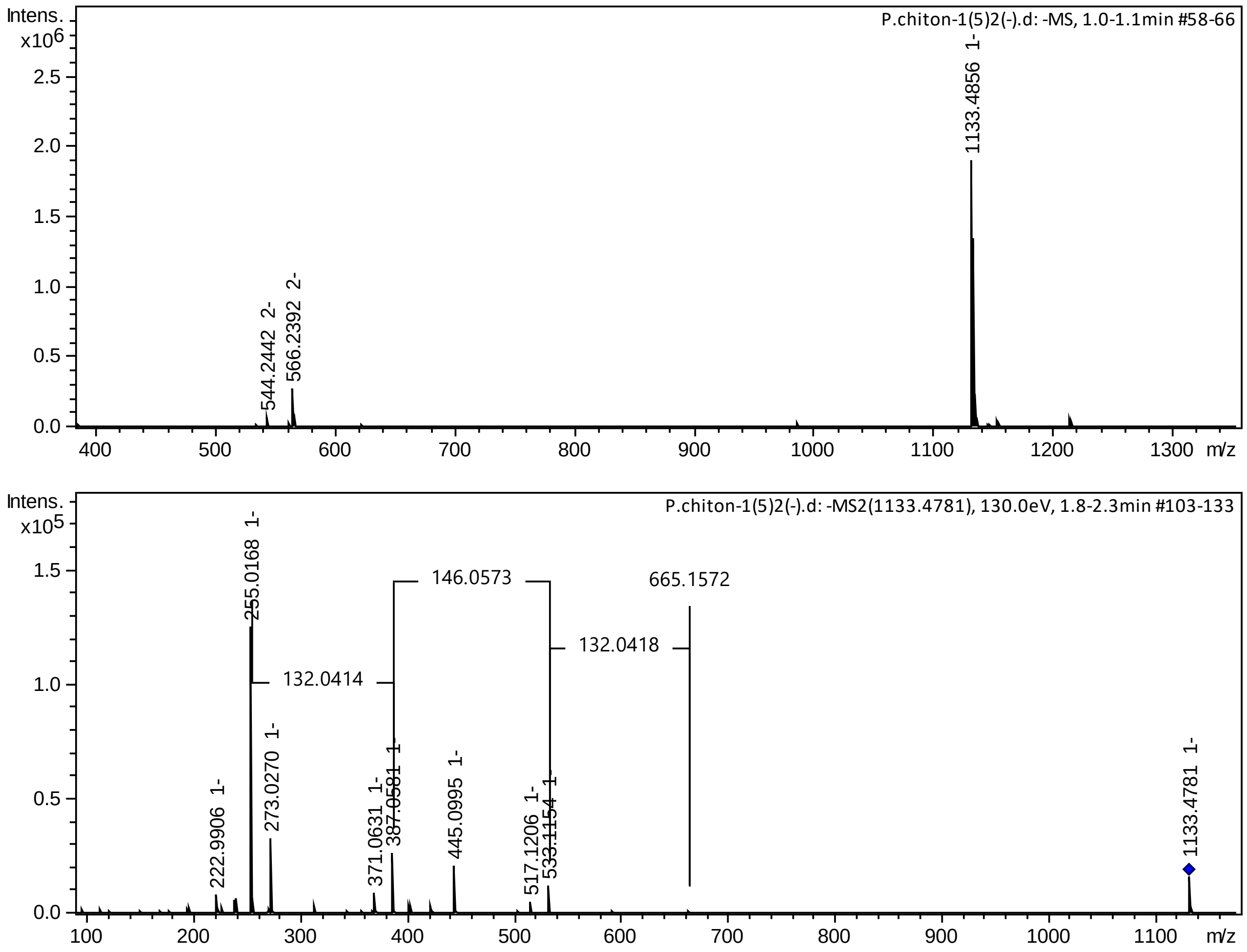

Figure S14. HR-ESI-MS and ESI-MS/MS spectra of chitonoidoside A_1 (**2**)

Table S2. ^{13}C and ^{1}H NMR chemical shifts, HMBC and ROESY correlations of the aglycone moiety of chitonoidoside B (**3**).

Position	δ_C mult. ^{a}	δ_H mult. (*J* in Hz) ^{b}	HMBC	ROESY
1	35.9 CH_2	1.61 m		H-19
		1.30 m		H-3, H-11
2	26.7 CH_2	2.09 m		
3	88.7 CH	3.12 dd (3.5; 11.7)		H-5, H-31, H1-Xyl1
4	39.6 C			
5	52.7 CH	0.76 brd (12.4)	C: 6, 10, 30	H-1, H-3, H-7, H-31
6	20.9 CH_2	1.59 m		H-31
7	28.7 CH_2	1.58 m		
		1.11 m		H-5, H-32
8	40.9 CH	2.33 m	C: 9	H-18, H-19
9	150.9 C			
10	39.4 C			
11	114.7 CH	5.30 d (5.7)	C: 8	H-1
12	33.8 CH_2	2.38 m	C: 13, 18	H-32
		2.26 dd (6.4; 16.4)	C: 11	H-18, H-21
13	56.3 C			
14	40.3 C			
15	50.4 CH_2	2.47 d (15.7)	C: 14, 16, 32	H-18
		2.18 d (15.7)	C: 13, 16	H-7, H-32
16	218.0 C			
17	63.8 CH	2.36 s	C: 16, 18, 20, 21	H-21, H-22, H-32
18	73.8 CH_2	4.03 d (10.0)	C: 20	H-8, H-15
		3.65 d (10.0)	C: 12, 20	H-12, H-19, H-21
19	22.7 CH_3	1.00 s	C: 1, 5, 9, 10	H-1, H-2, H-6, H-8, H-18
20	86.7 C			
21	26.1 CH_3	1.32 s	C: 17, 20, 22	H-12, H-17, H-18, H-22
22	37.8 CH_2	1.70 m		H-21
		1.58 m		H-21
23	22.1 CH_2	1.71 m		
		1.57 m		
24	38.2 CH_2	1.95 m		
25	145.9 C			
26	110.1 CH_2	4.72 brs	C: 24, 27	H-21, H-24
		4.71 brs	C: 24, 27	
27	22.2 CH_3	1.66 s	C: 24, 25, 26	H-26
30	16.4 CH_3	0.99 s	C: 3, 4, 5, 31	H-2, H-6, H-31
31	27.9 CH_3	1.14 s	C: 3, 4, 5, 30	H-3, H-5, H-6, H-30, H-1 Xyl1
32	21.4 CH_3	0.79 s	C: 13, 14, 15	H-7, H-15, H-17

[a]Recorded at 176.03 MHz in C_5D_5N/D_2O (4/1). [b]Recorded at 700.00 MHz in C_5D_5N/D_2O (4/1).

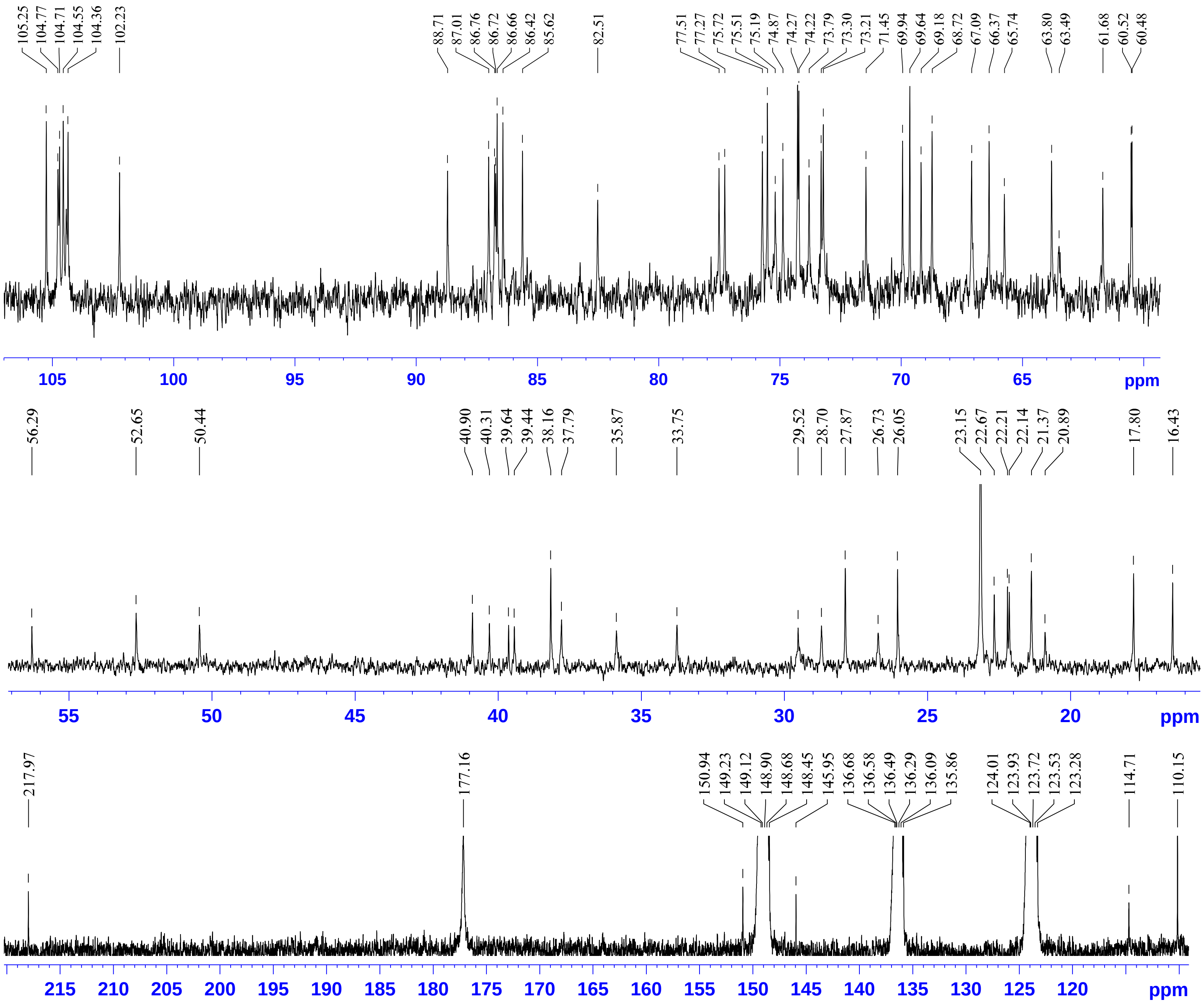

Figure S15. The ^{13}C NMR (176.03 MHz) spectrum of chitonoidoside B (**3**) in C_5D_5N/D_2O (4/1)

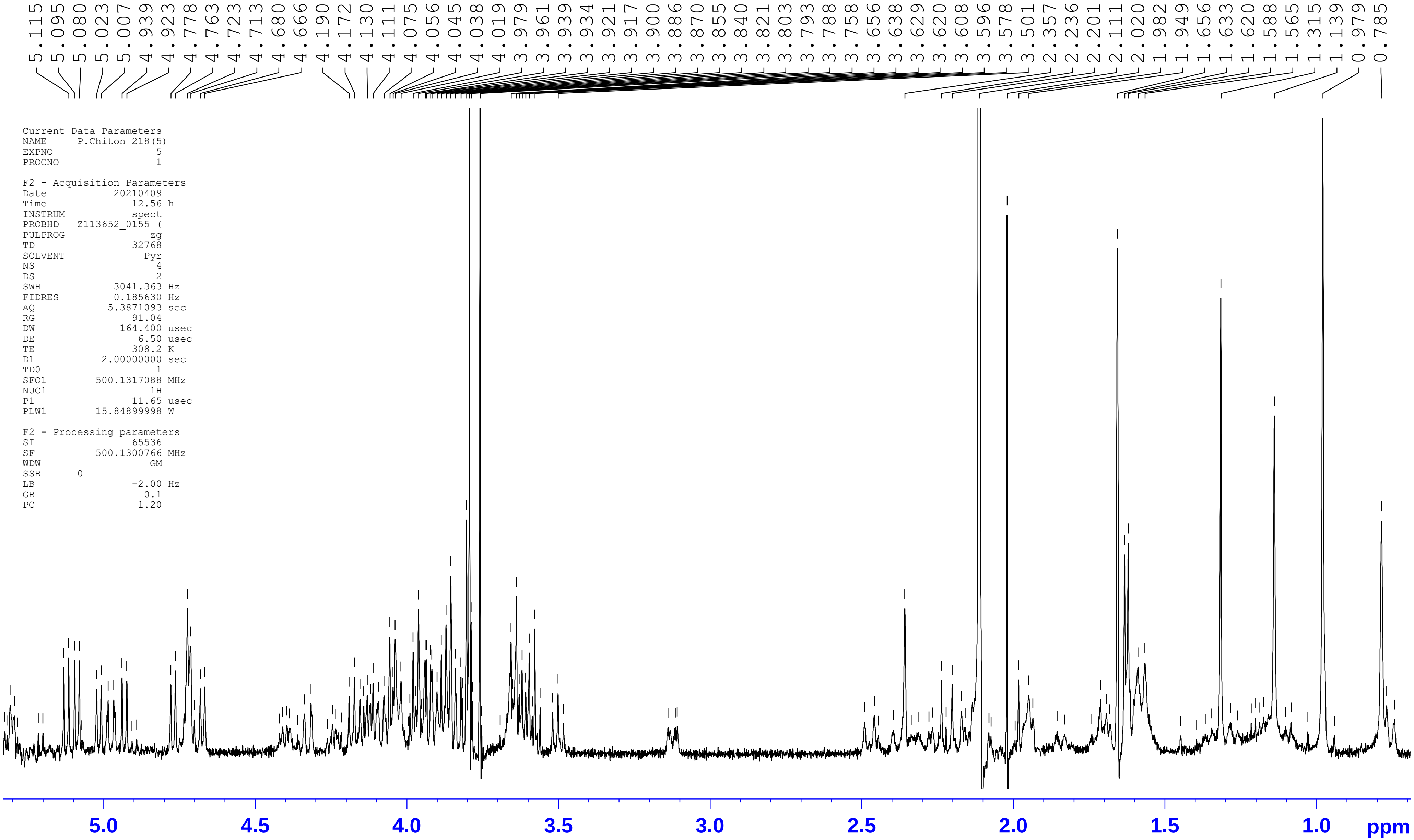

Figure S16. The ^{1}H NMR (700.00 MHz) spectrum of chitonoidoside B (**3**) in C_5D_5N/D_2O (4/1)

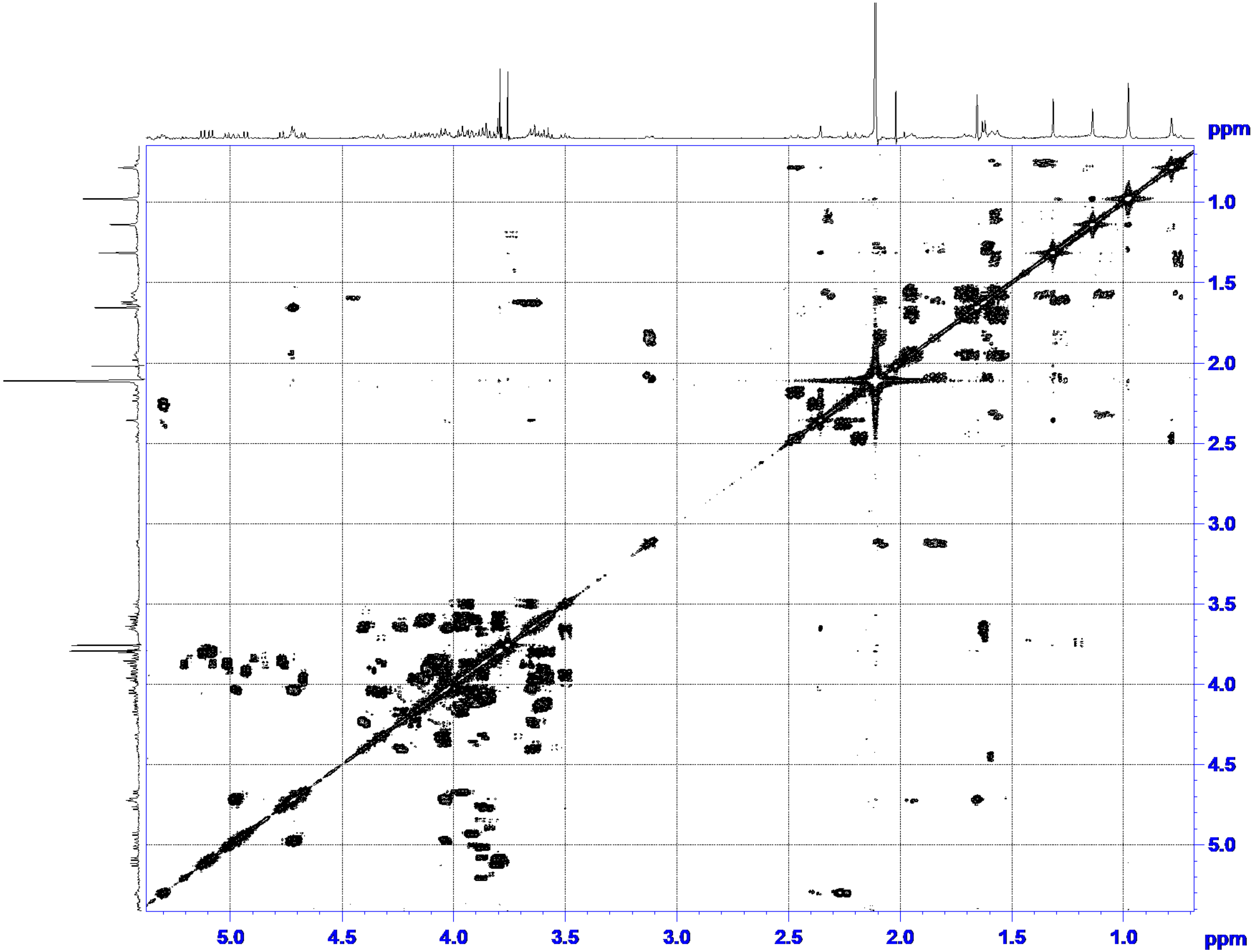

Figure S17. The COSY (700.00 MHz) spectrum of chitonoidoside B (**3**) in C_5D_5N/D_2O (4/1)

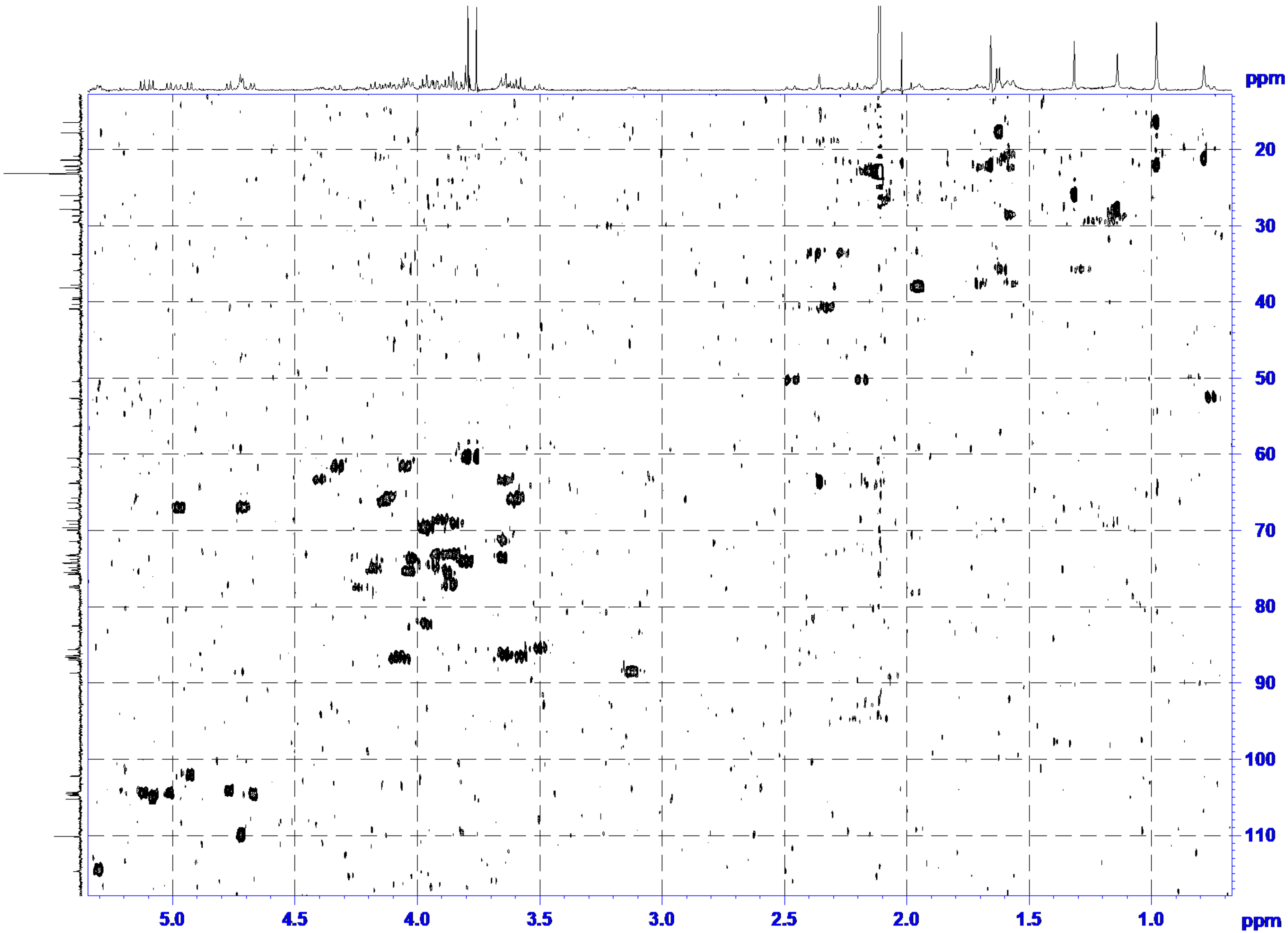

Figure S18. The HSQC (700.00 MHz) spectrum of chitonoidoside B (**3**) in C_5D_5N/D_2O (4/1)

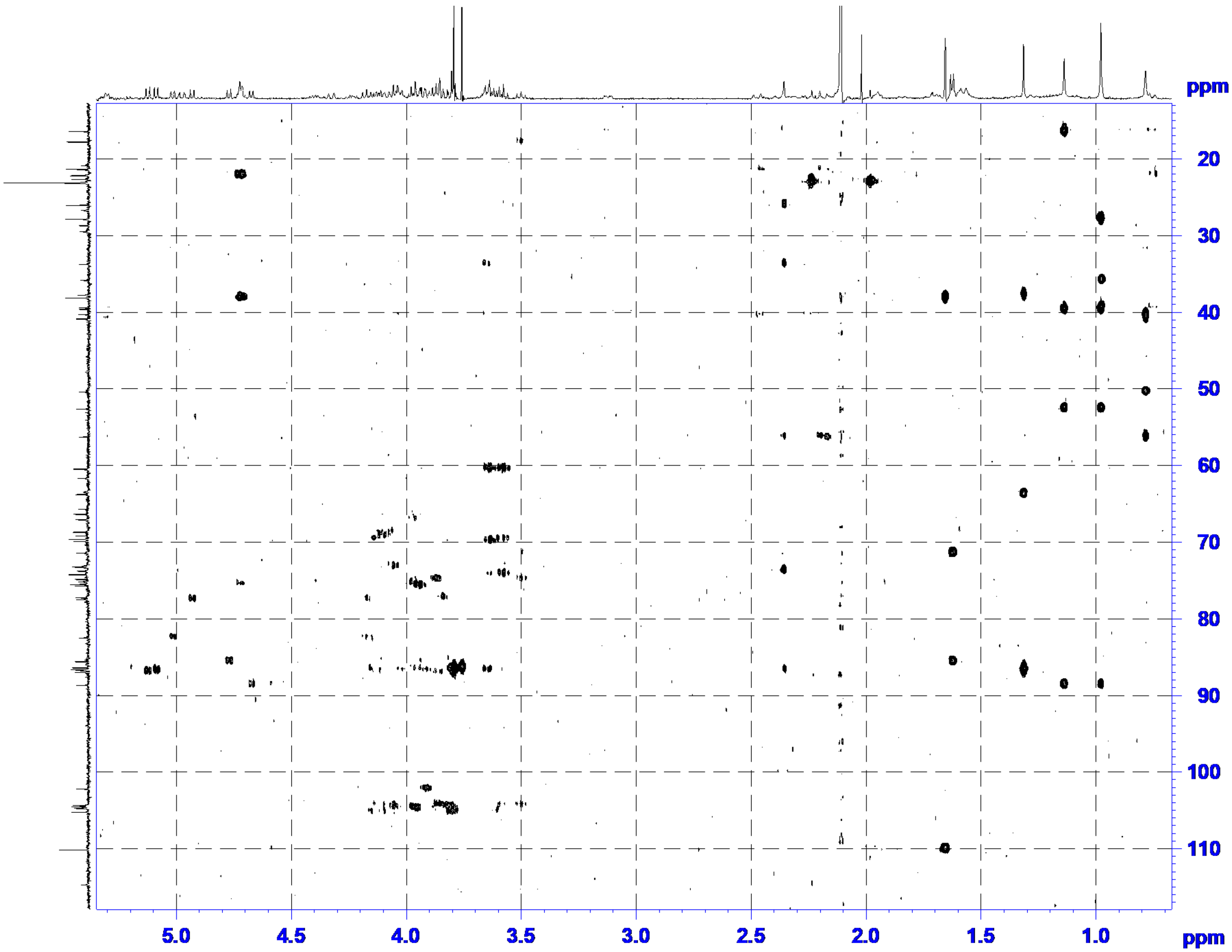

Figure S19. The HMBC (700.00 MHz) spectrum of chitonoidoside B (**3**) in C_5D_5N/D_2O (4/1)

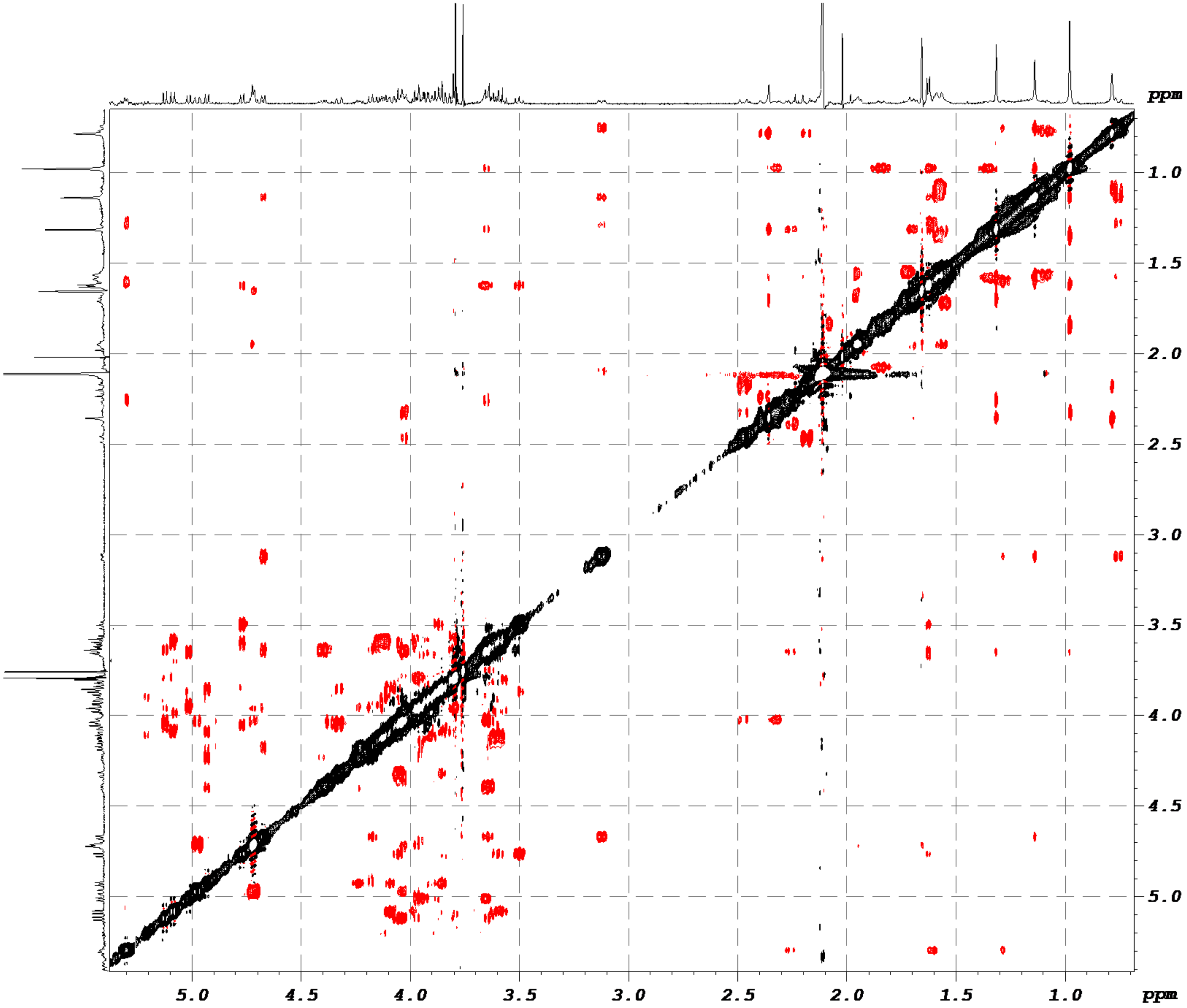

Figure S20. The ROESY (700.00 MHz) spectrum of chitonoidoside B (**3**) in C_5D_5N/D_2O (4/1)

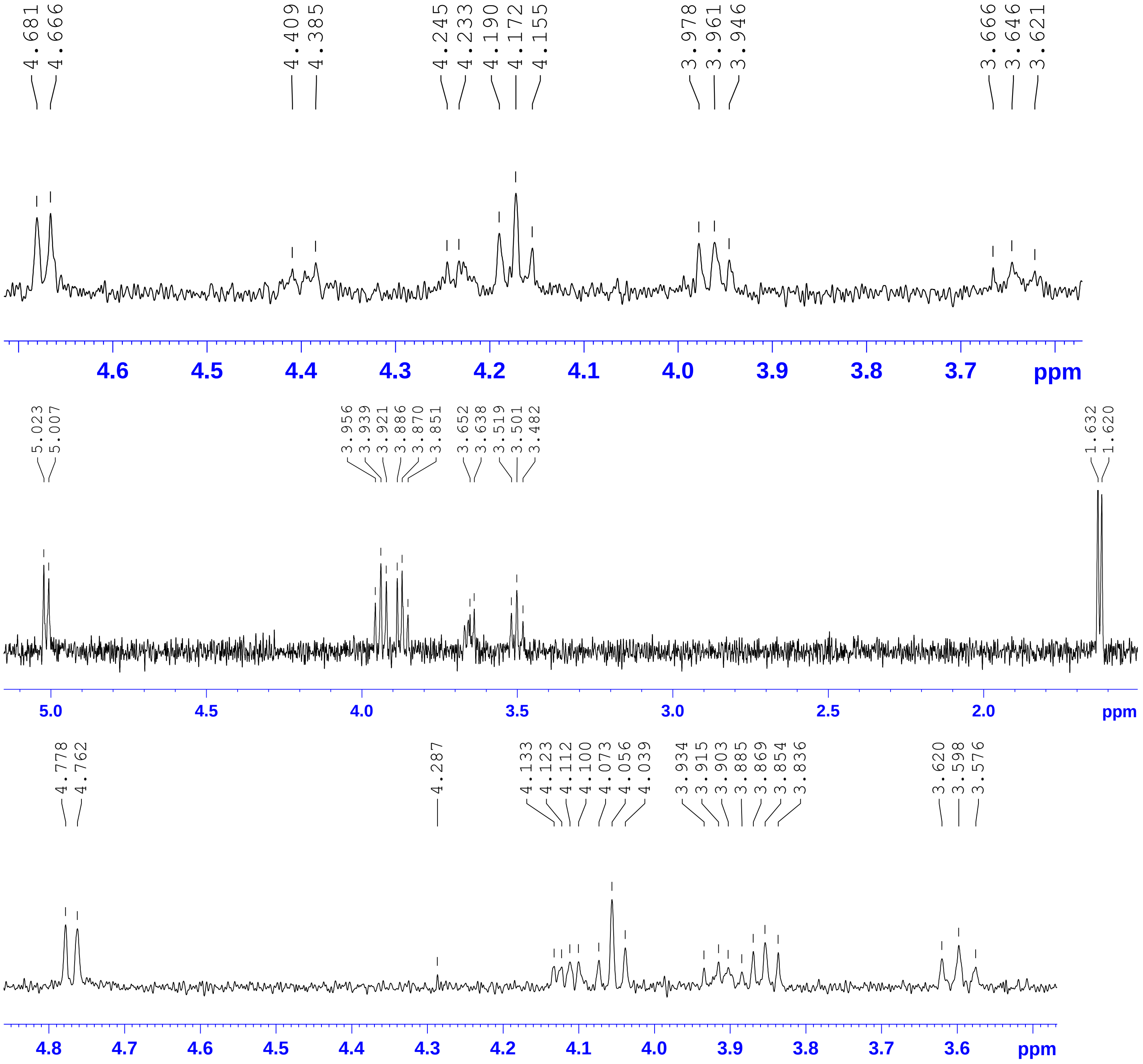

Figure S21. 1 D TOCSY (700.00 MHz) spectra of Xyl1, Qui2 and Xyl3 of chitonoidoside B (**3**) in C_5D_5N/D_2O (4/1)

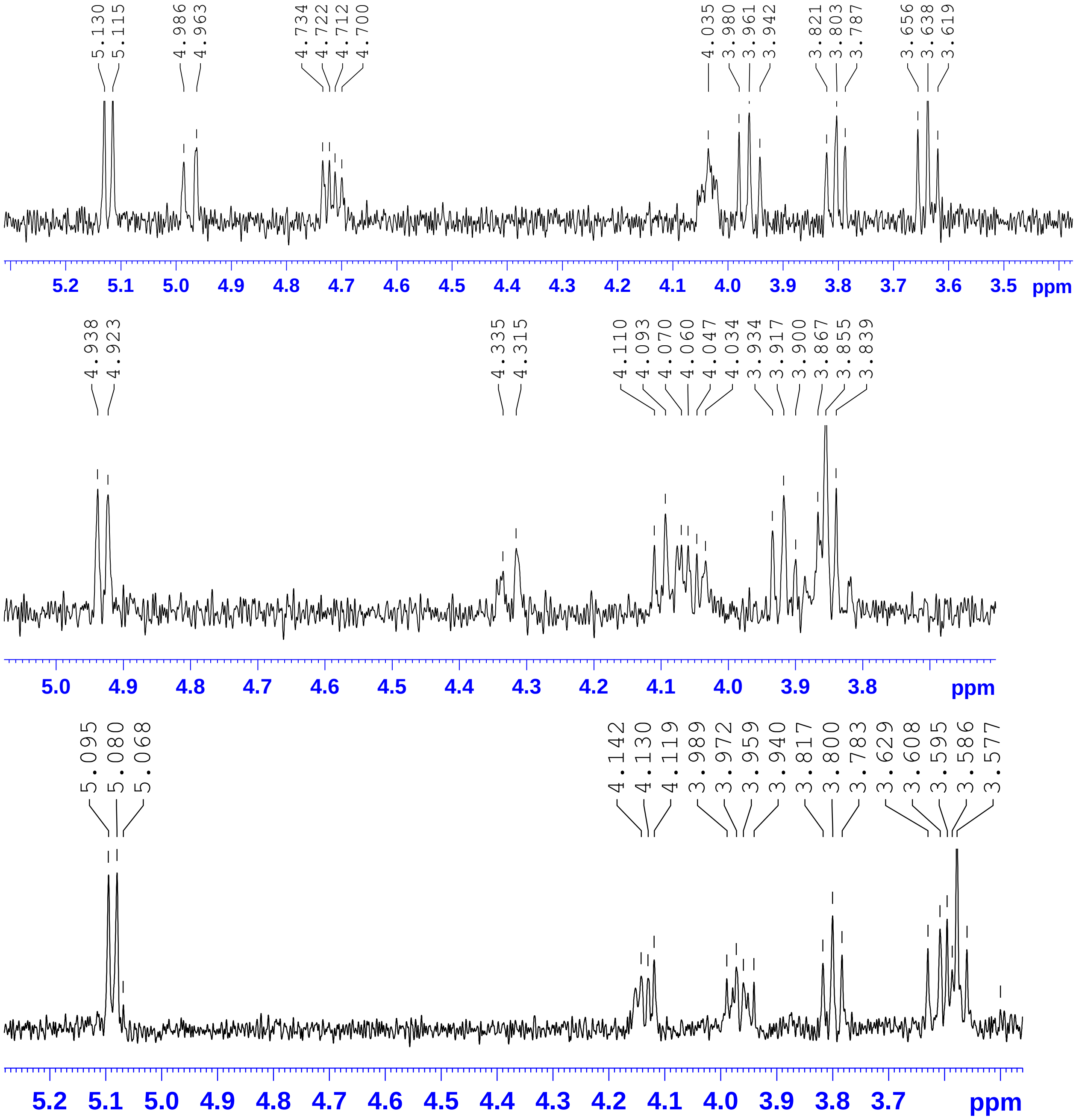

Figure S22. 1 D TOCSY (700.00 MHz) spectra of MeGlc4, Glc5 and MeXyl6 of chitonoidoside B (**3**) in C_5D_5N/D_2O (4/1)

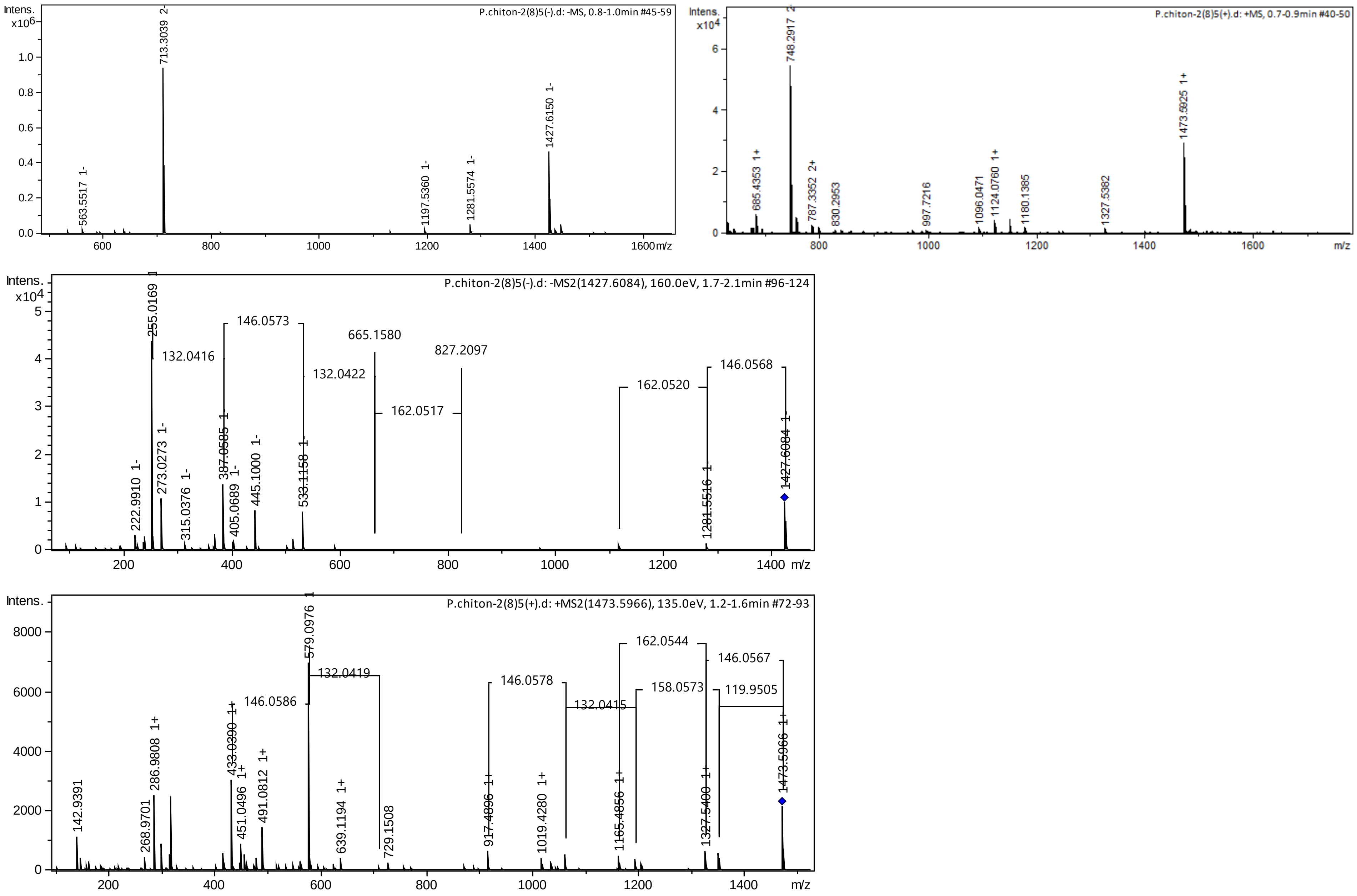

Figure S23. HR-ESI-MS and ESI-MS/MS spectra of chitonoidoside B (**3**)

Table S3. ^{13}C and ^{1}H NMR chemical shifts, HMBC and ROESY correlations of the aglycone moiety of chitonoidoside C (**4**).

Position	δ_C mult.[a]	δ_H mult. (*J* in Hz) [b]	HMBC	ROESY
1	36.0 CH_2	1.73 m		H-11, H-19
		1.32 m		H-11
2	26.7 CH_2	2.08 m		
		1.87 m		H-19, H-30
3	88.7 CH	3.14 dd (4.6; 11.7)		H-1, H-31, H1-Xyl1
4	39.5 C			
5	52.7 CH	0.79 brd (11.5)	C: 10, 19, 30	H-1, H-3, H-31
6	20.9 CH_2	1.58 m		H-31
		1.39 m		H-19, H-30
7	28.3 CH_2	1.57 m		
		1.16 m		H-5, H-32
8	38.6 CH	3.12 m		H-15, H-19
9	150.9 C			
10	39.7 C			
11	111.1 CH	5.28 m	C: 8, 13	H-1
12	31.9 CH_2	2.62 d (16.9)	C: 18	H-17, H-32
		2.47 dd (5.5; 16.9)	C: 14	H-21
13	55.8 C			
14	42.0 C			
15	51.9 CH_2	2.38 d (15.5)	C: 13, 16, 17, 32	H-7, H-32
		2.09 d (15.5)	C: 8, 14, 16, 32	H-8
16	214.5 C			
17	61.2 CH	2.86 s	C: 12, 13, 16, 18, 20, 21	H-12, H-21, H-32
18	176.7 C			
19	21.9 CH_3	1.27 s	C: 1, 5, 9, 10	H-1, H-2, H-8, H-30
20	83.6 C			
21	26.7 CH_3	1.43 s	C: 17, 20, 22	H-12, H-17
22	38.1 CH_2	1.71 m		
		1.55 m		
23	22.1 CH_2	1.70 m		
		1.43 m		
24	37.7 CH_2	1.89 m	C: 22, 25, 26, 27	H-21
25	145.5 C			
26	110.4 CH_2	4.70 brs	C: 24, 27	
		4.68 brs	C: 24, 27	
27	22.0 CH_3	1.62 s	C: 24, 25, 26	
30	16.5 CH_3	0.99 s	C: 3, 4, 5, 31	H-31
31	27.9 CH_3	1.14 s	C: 3, 4, 5, 30	H-3, H-5, H-30
32	20.5 CH_3	0.88 s	C: 8, 13, 14, 15	H-7, H-12, H-17

[a]Recorded at 176.03 MHz in C_5D_5N/D_2O (4/1). [b]Recorded at 700.00 MHz in C_5D_5N/D_2O (4/1).

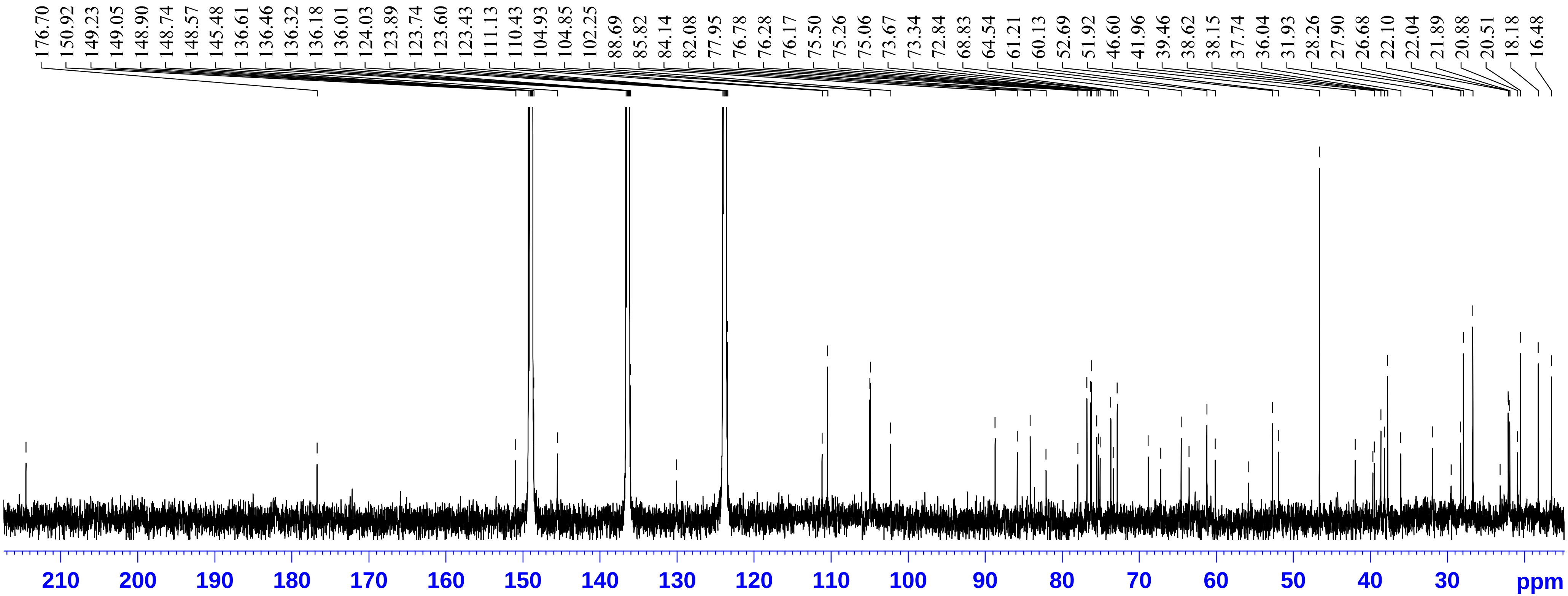

Figure S24. The ^{13}C NMR (176.03 MHz) spectrum of chitonoidoside C (**4**) in C_5D_5N/D_2O (4/1)

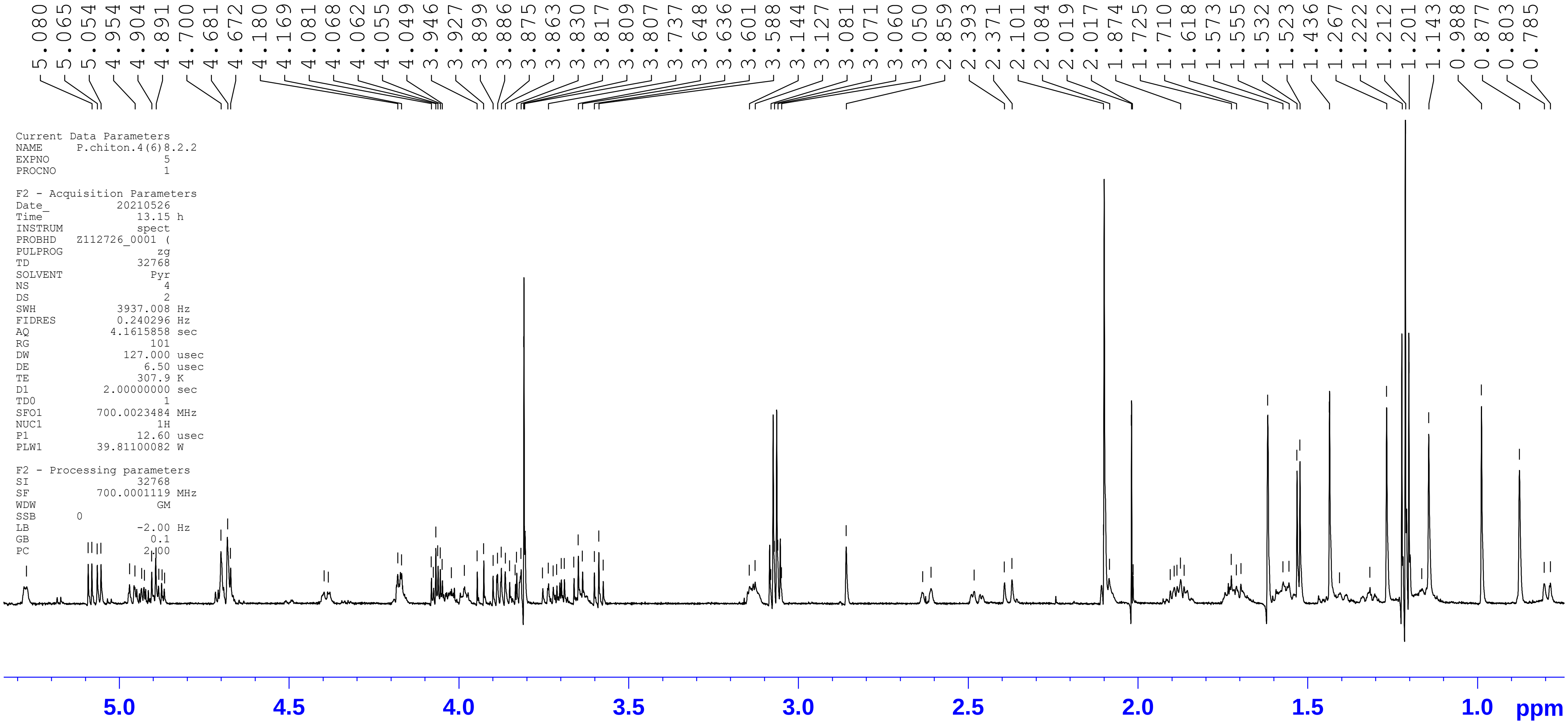

Figure S25. The ^{1}H NMR (700.00 MHz) spectrum of chitonoidoside C (**4**) in C_5D_5N/D_2O (4/1)

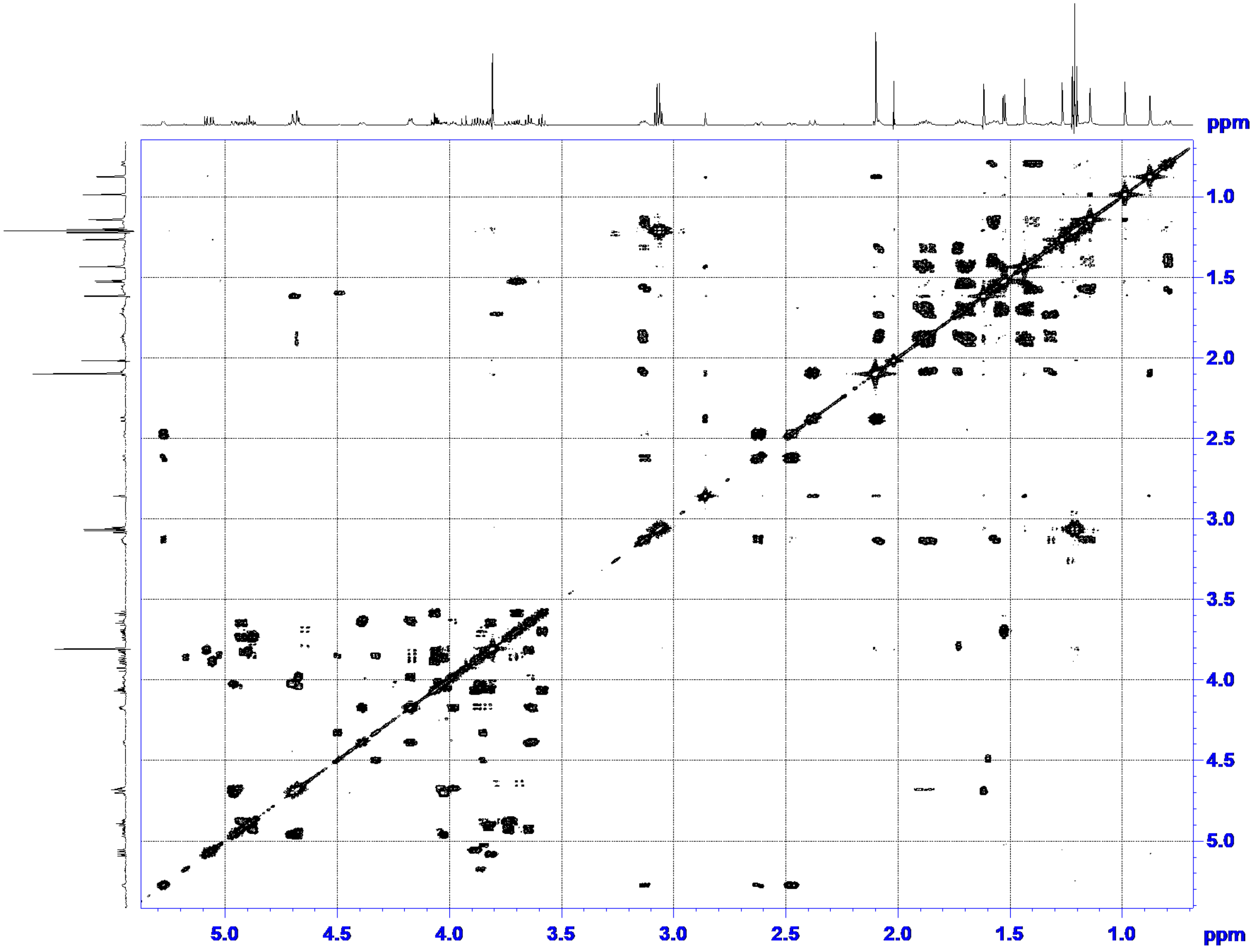

Figure S26. The COSY (700.00 MHz) spectrum of chitonoidoside C (**4**) in C_5D_5N/D_2O (4/1)

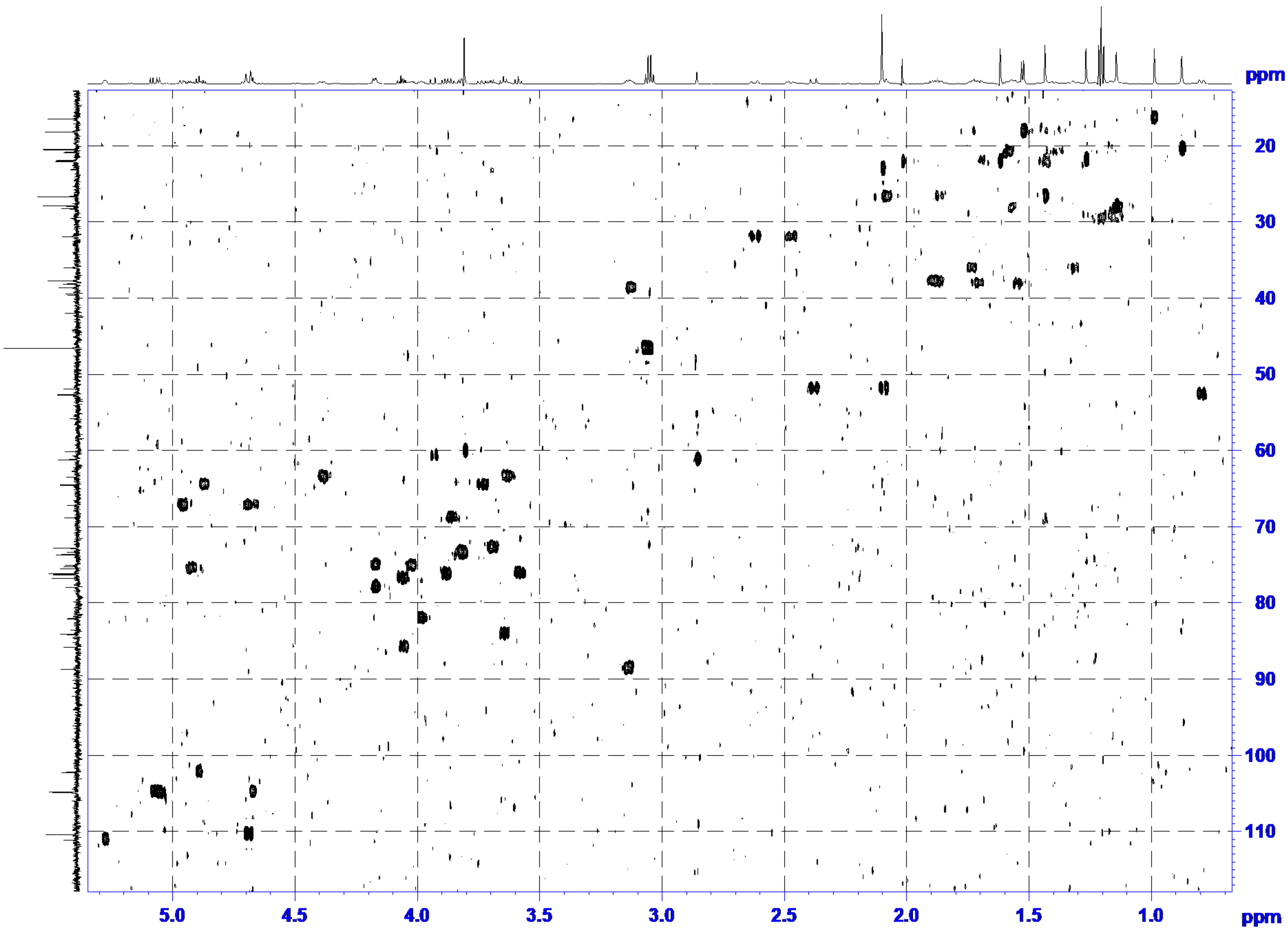

Figure S27. The HSQC (700.00 MHz) spectrum of chitonoidoside C (**4**) in C_5D_5N/D_2O (4/1)

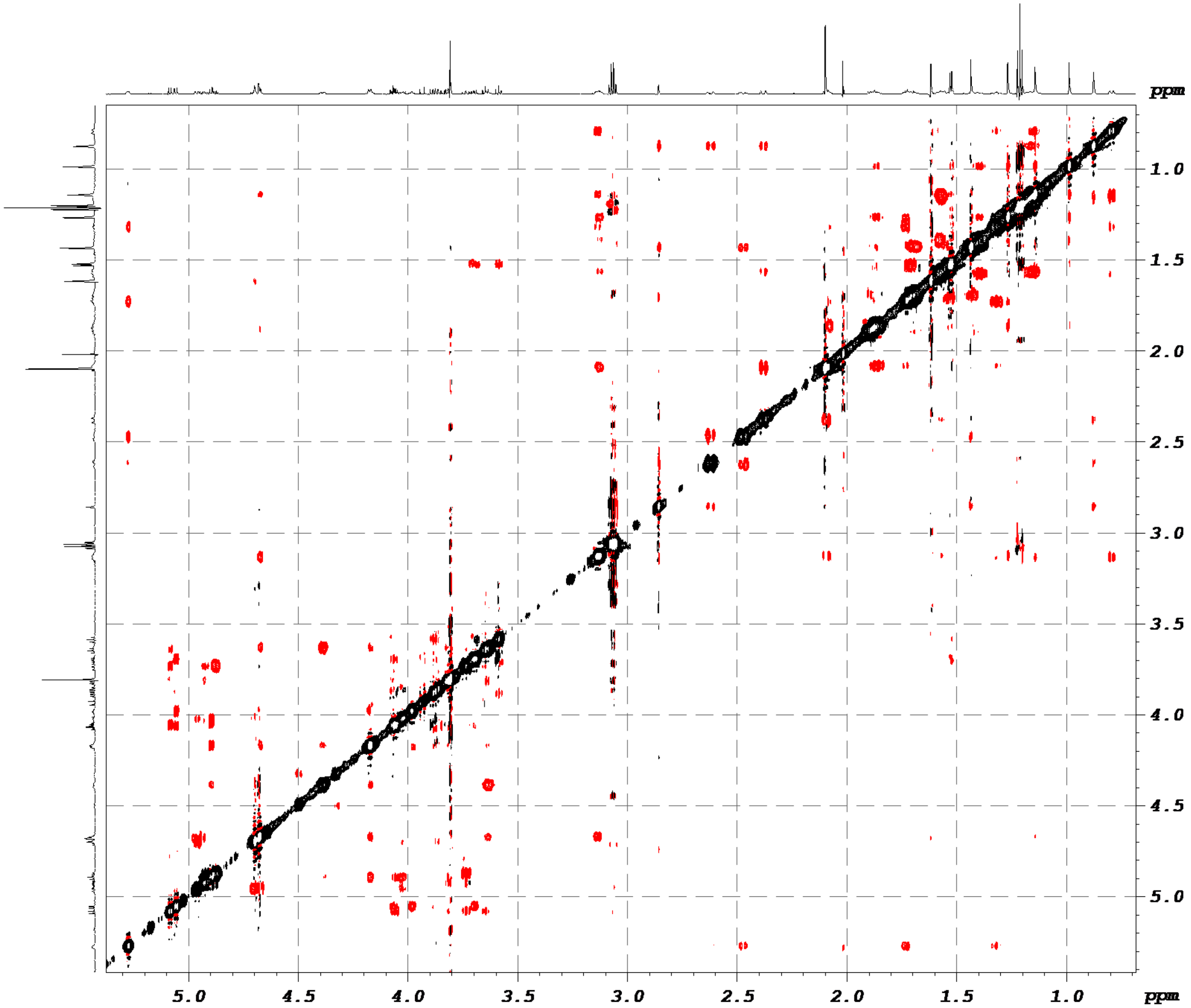

Figure S28. The ROESY (700.00 MHz) spectrum of chitonoidoside C (**4**) in C_5D_5N/D_2O (4/1)

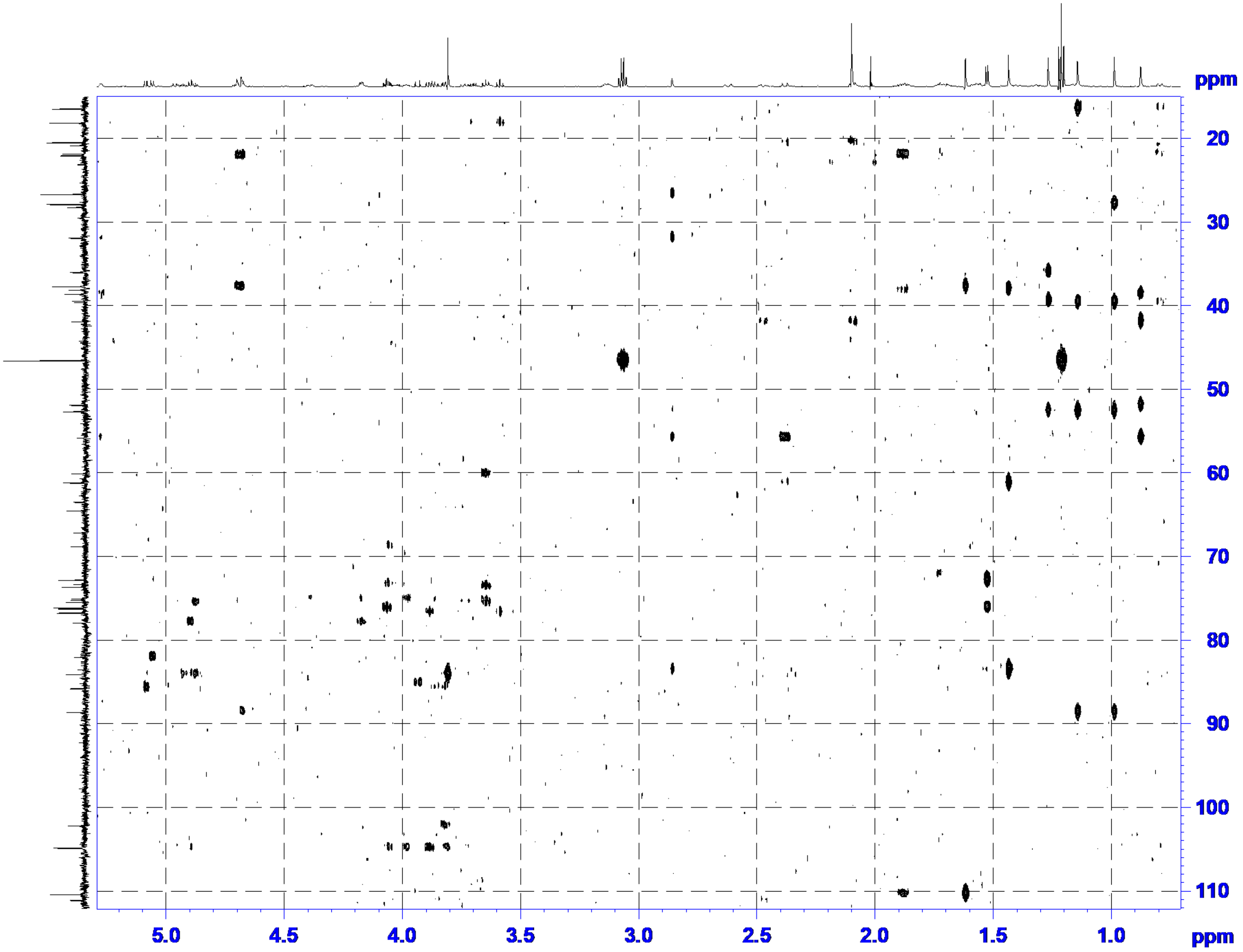

Figure S29. The HMBC (700.00 MHz) spectrum of chitonoidoside C (**4**) in C_5D_5N/D_2O (4/1)

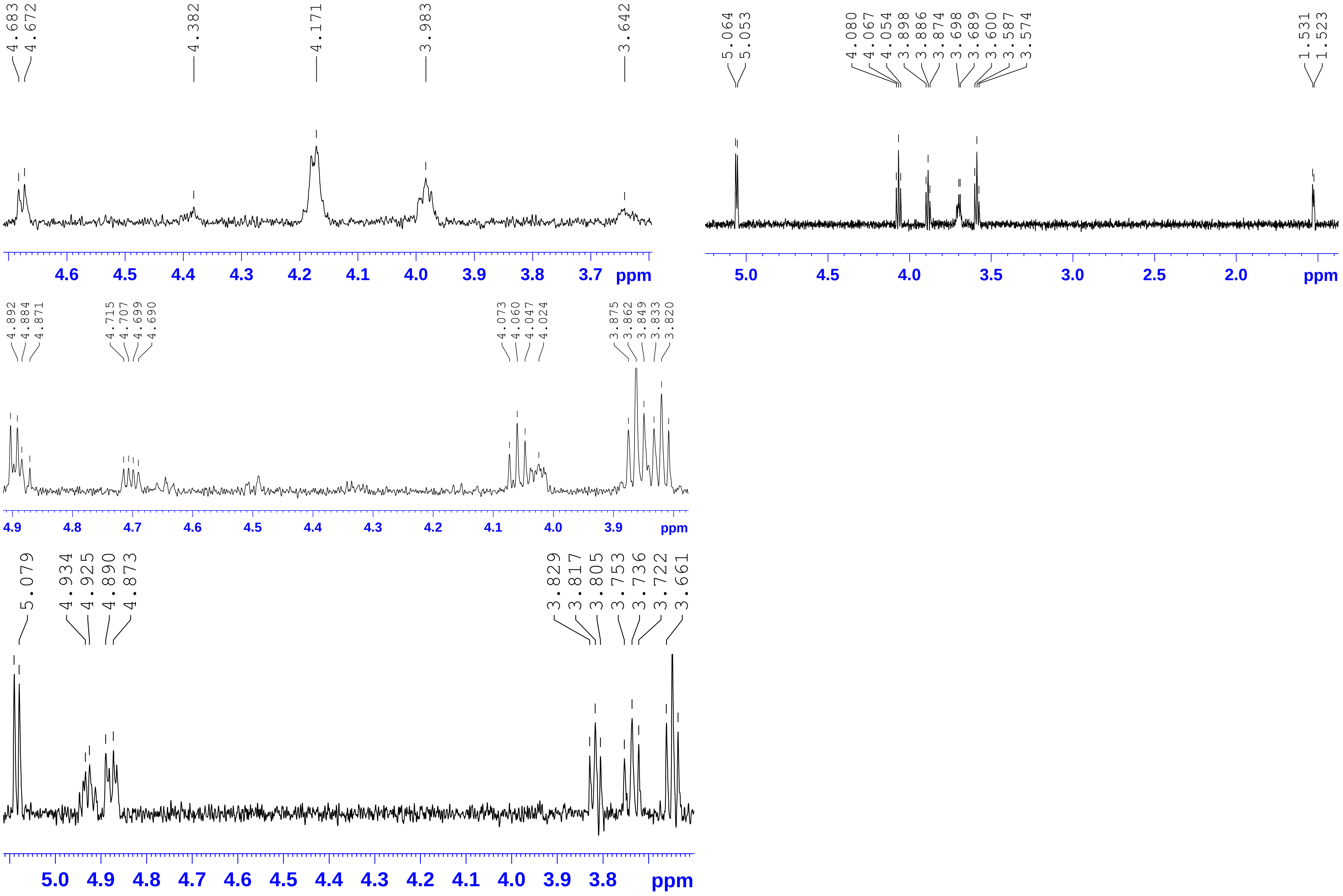

Figure S30. 1 D TOCSY (700.00 MHz) spectra of chitonoidoside C (**4**) in C_5D_5N/D_2O (4/1)

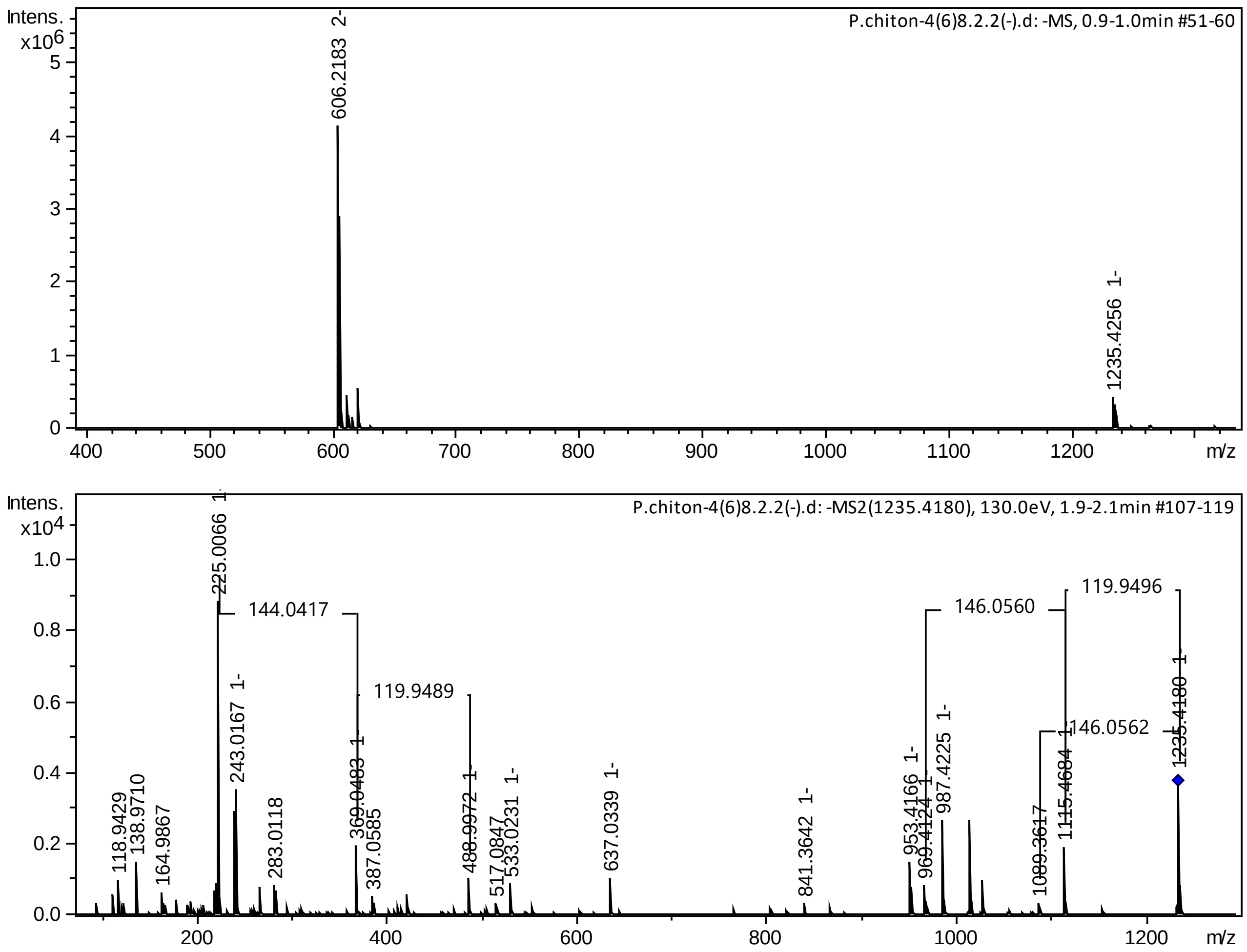

Figure S31. HR-ESI-MS and ESI-MS/MS spectra of chitonoidoside C (**4**)

Table S4. ^{13}C and ^{1}H NMR chemical shifts, HMBC and ROESY correlations of the aglycone moiety of chitonoidoside D (**5**).

Position	δ_C mult.[a]	δ_H mult. (*J* in Hz) [b]	HMBC	ROESY
1	36.1 CH_2	1.73 m		H-11
		1.31 m		H-11
2	26.7 CH_2	2.07 m		
		1.86 m		
3	88.8 CH	3.13 dd (4.6; 11.7)	C: 4, 30, 31, C:1 Xyl1	H-5, H-31, H1-Xyl1
4	39.5 C			
5	52.7 CH	0.79 brd (11.7)	C: 6, 10, 30	H-3, H-31
6	20.9 CH_2	1.58 m		H-31
		1.39 m		
7	28.3 CH_2	1.58 m		
		1.17 m		H-32
8	38.7 CH	3.11 m		H-15, H-19
9	151.0 C			
10	39.7 C			
11	111.2 CH	5.27 d (4.6)	C: 8, 13	H-1
12	32.0 CH_2	2.63 d (17.3)	C: 9, 13, 18	H-17, H-32
		2.47 dd (6.5; 17.3)	C: 9, 11, 13, 14, 18	H-21
13	55.9 C			
14	42.0 C			
15	52.0 CH_2	2.40 d (15.0)	C: 13, 16, 17, 32	H-32
		2.10 d (15.0)	C: 8, 14, 16, 32	H-8
16	214.7 C			
17	61.3 CH	2.86 s	C: 12, 13, 16, 18, 20, 21	H-12, H-21, H-22, H-32
18	176.8 C			
19	21.9 CH_3	1.26 s	C: 1, 5, 9, 10	H-1, H-2, H-8, H-30
20	83.7 C			
21	26.7 CH_3	1.44 s	C: 17, 20, 22	H-12, H-17
22	38.2 CH_2	1.71 m	C: 20, 21, 23	
		1.54 m	C: 20, 21	
23	22.2 CH_2	1.69 m		
		1.44 m		
24	37.8 CH_2	1.88 m	C: 23, 25, 26, 27	
25	145.6 C			
26	110.5 CH_2	4.70 brs	C: 24, 27	
		4.67 brs	C: 24, 27	
27	22.1 CH_3	1.62 s	C: 24, 25, 26	
30	16.6 CH_3	0.98 s	C: 3, 4, 5, 31	H-31
31	28.0 CH_3	1.14 s	C: 3, 4, 5, 30	H-3, H-5, H-30
32	20.6 CH_3	0.88 s	C: 8, 13, 14, 15	H-7, H-12, H-17

[a]Recorded at 176.03 MHz in C_5D_5N/D_2O (4/1). [b]Recorded at 700.00 MHz in C_5D_5N/D_2O (4/1).

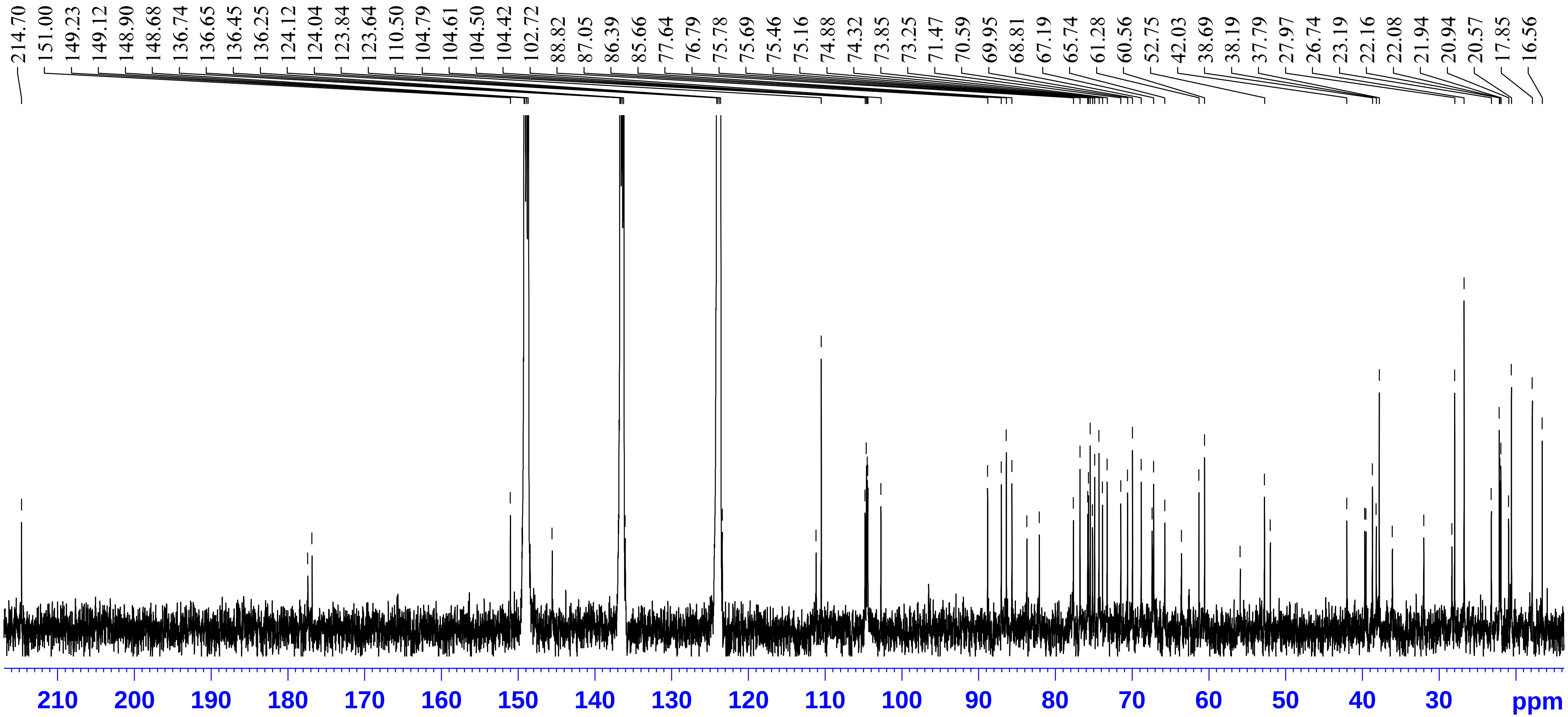

Figure S32. The ^{13}C NMR (176.03 MHz) spectrum of chitonoidoside D (**5**) in C_5D_5N/D_2O (4/1)

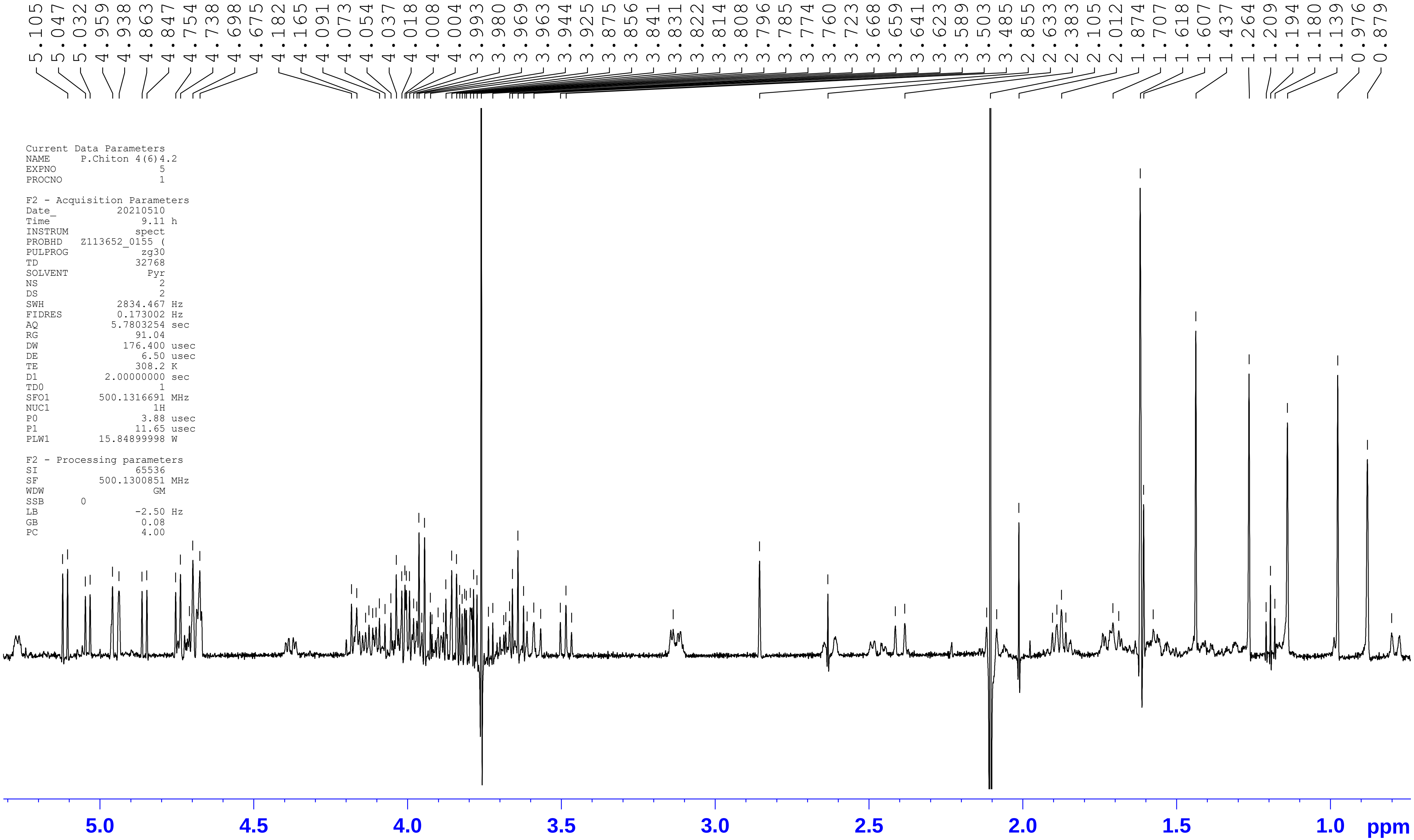

Figure S33. The ¹H NMR (700.00 MHz) spectrum of chitonoidoside D (**5**) in C_5D_5N/D_2O (4/1)

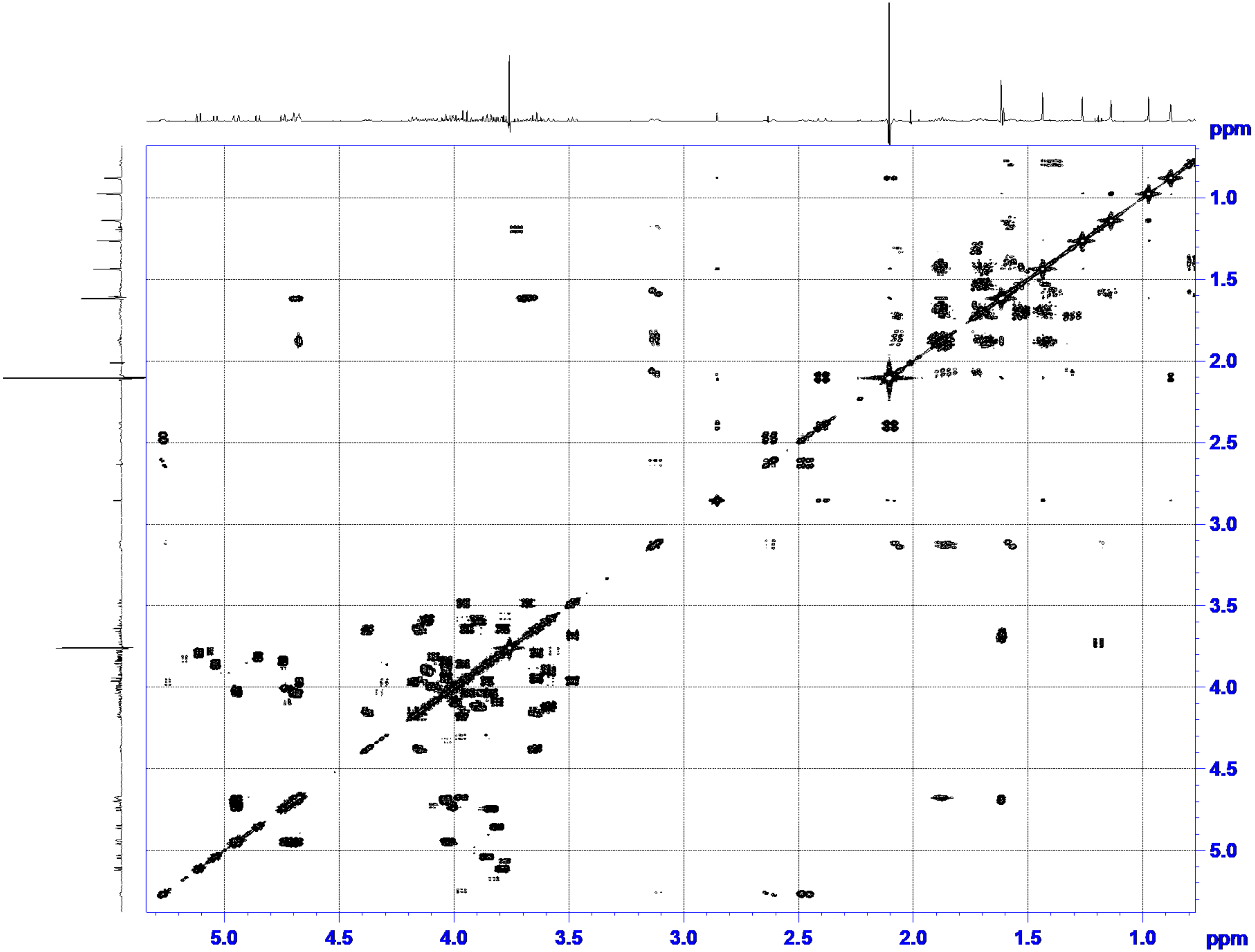

Figure S34. The COSY (700.00 MHz) spectrum of chitonoidoside D (**5**) in C_5D_5N/D_2O (4/1)

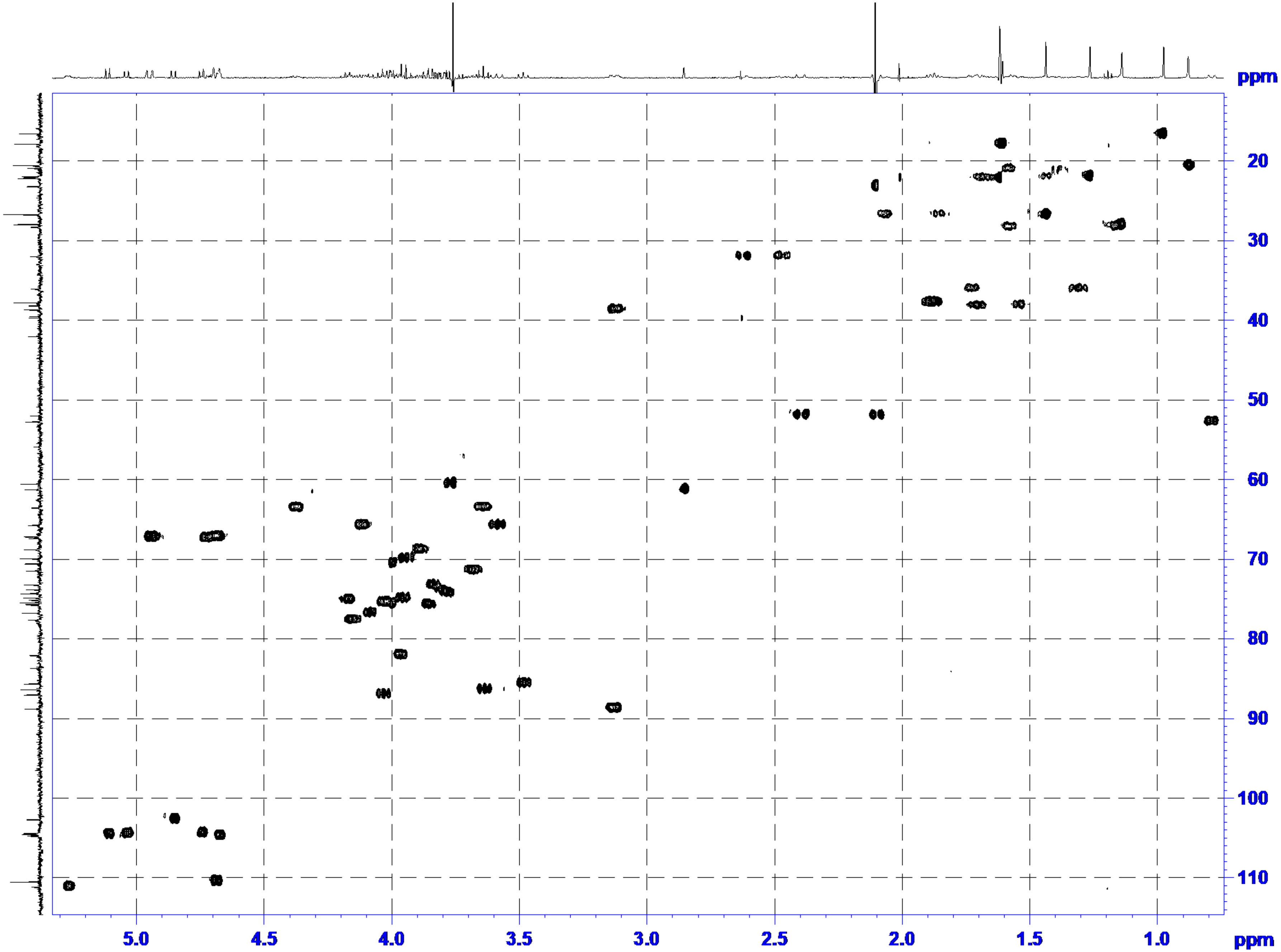

Figure S35. The HSQC (700.00 MHz) spectrum of chitonoidoside D (**5**) in C_5D_5N/D_2O (4/1)

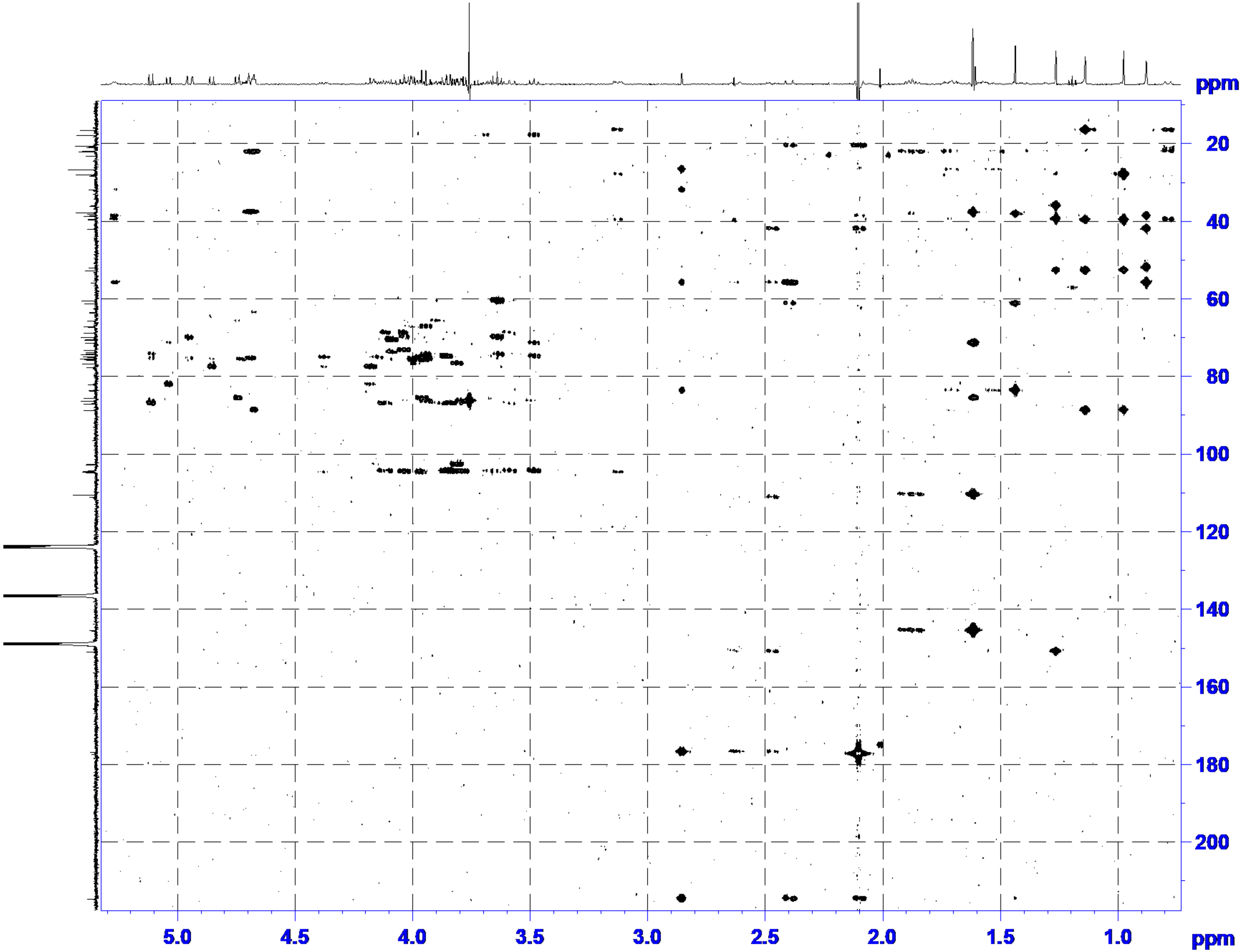

Figure S36. The HMBC (700.00 MHz) spectrum of chitonoidoside D (**5**) in C_5D_5N/D_2O (4/1)

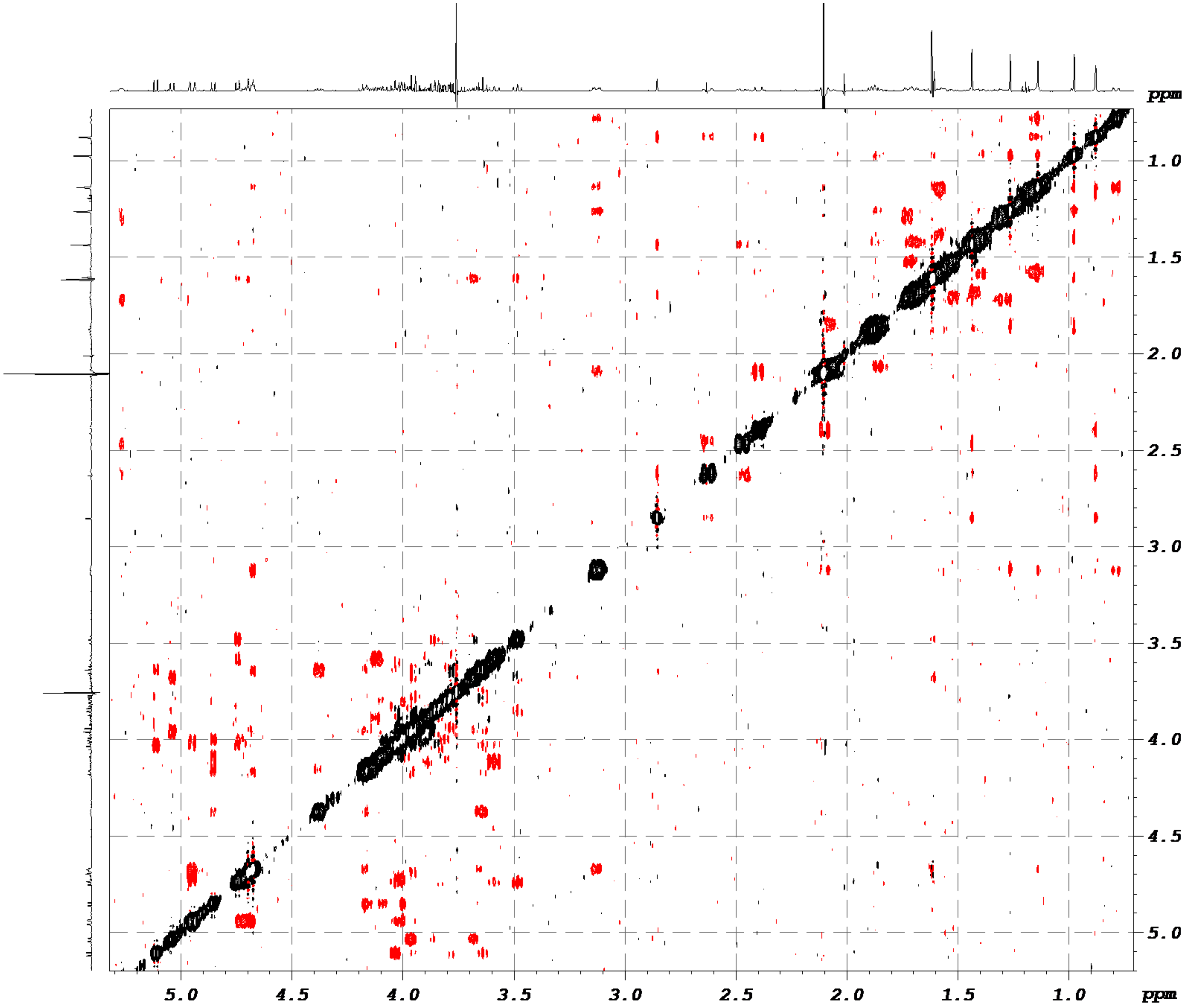

Figure S37. The ROESY (700.00 MHz) spectrum of chitonoidoside D (**5**) in C_5D_5N/D_2O (4/1)

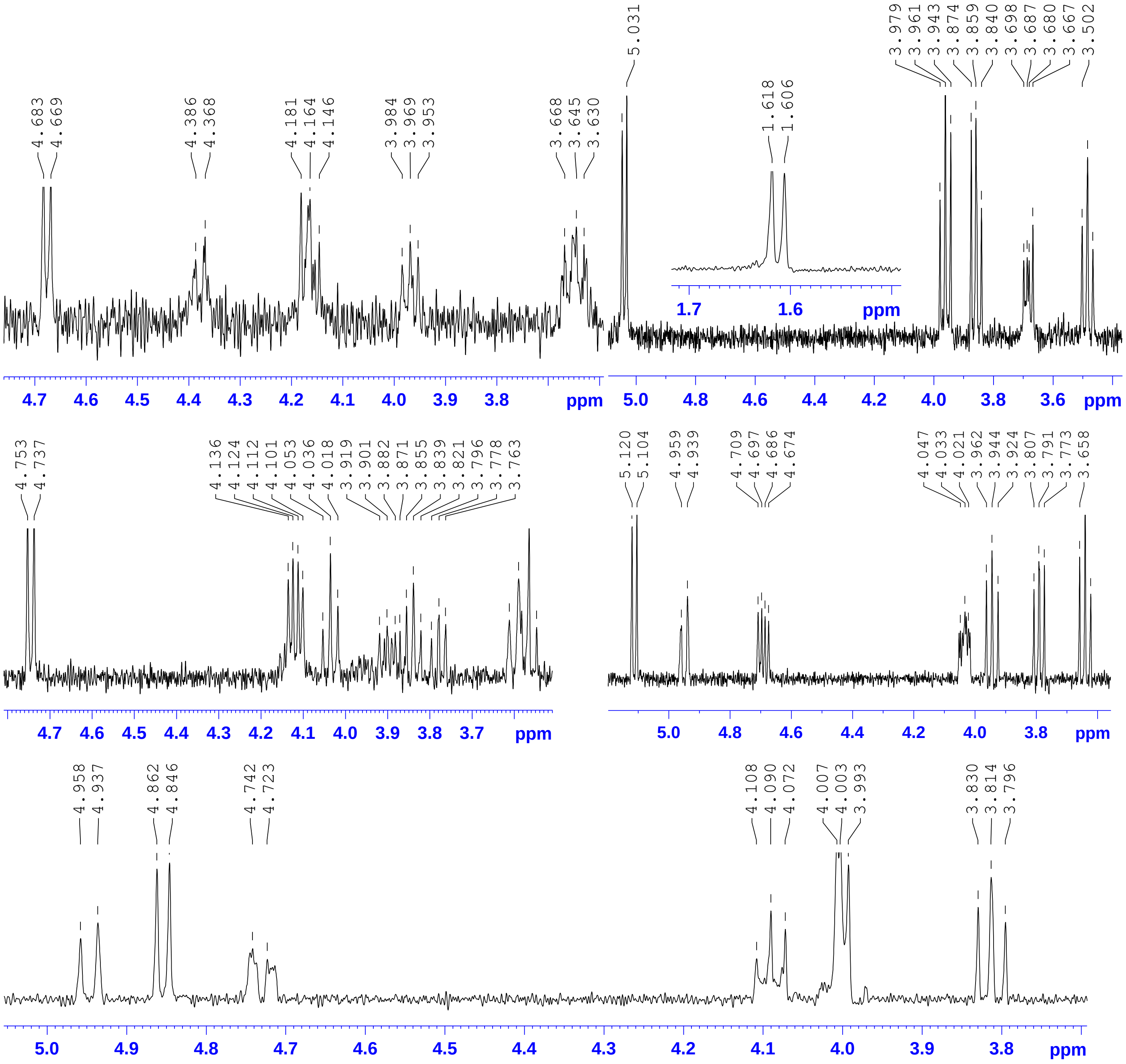

Figure S38. 1D TOCSY (700.00 MHz) spectra of chitonoidoside D (**5**) in C_5D_5N/D_2O (4/1)

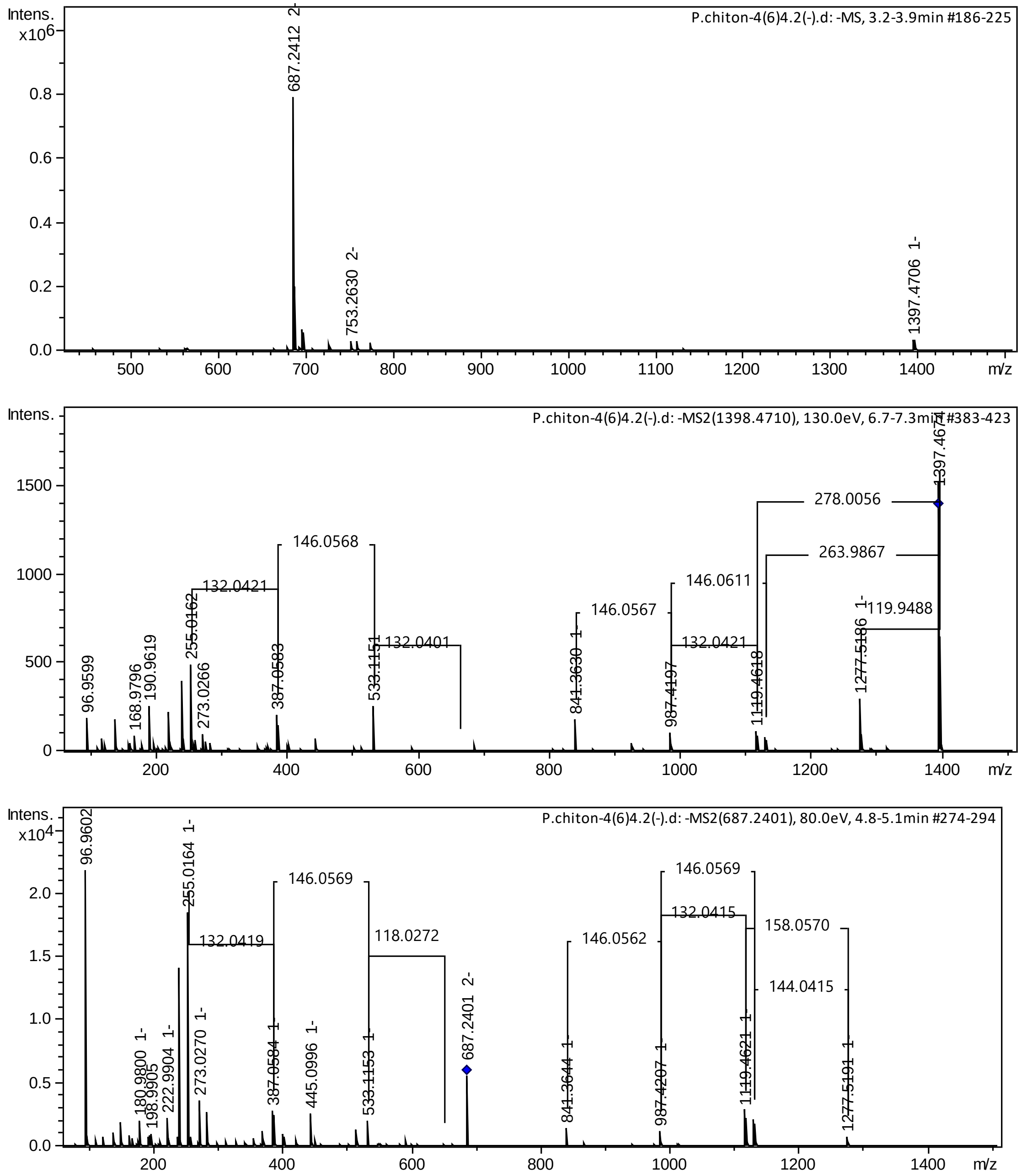

Figure S39. HR-ESI-MS and ESI-MS/MS spectra of chitonoidoside D (**5**)

Table S5. ^{13}C and ^{1}H NMR chemical shifts, HMBC and ROESY correlations of the aglycone moiety of chitonoidoside E (**6**).

Position	δ_C mult. [a]	δ_H mult. (*J* in Hz) [b]	HMBC	ROESY
1	35.9 CH_2	1.60 m		H-11
		1.28 m		
2	26.7 CH_2	2.08 m		
		1.84 m		
3	88.7 CH	3.12 dd (4.2; 11.2)	C: 30, C:1 Xyl1	H-5, H-31, H1-Xyl1
4	39.6 C			
5	52.7 CH	0.75 brd (11.2)	C: 10, 19, 30	H-1, H-3, H-31
6	20.9 CH_2	1.57 m		
		1.34 m		H-19, H-30
7	28.7 CH_2	1.57 m		
		1.08 m		H-5, H-32
8	40.9 CH	2.31 m		H-18, H-19
9	150.9 C			
10	39.5 C			
11	114.7 CH	5.29 d (6.1)	C: 8, 10, 13	H-1
12	33.8 CH_2	2.38 m		H-32
		2.25 m	C: 9, 11, 13, 14	H-21
13	56.3 C			
14	40.3 C			
15	50.5 CH_2	2.46 d (15.5)	C: 14, 16, 32	H-8, H-18
		2.19 d (15.5)	C: 13, 16, 32	H-32
16	218.1 C			
17	63.8 CH	2.35 s	C: 12, 13, 16, 18, 20, 21	H-21, H-22, H-32
18	73.8 CH_2	4.02 m	C: 12, 14, 20	H-8, H-15
		3.65 d (9.4)	C: 12, 14, 20	H-21
19	22.2 CH_3	0.98 s	C: 1, 5, 9, 10	H-1, H-2, H-6, H-8, H-18
20	86.8 C			
21	26.1 CH_3	1.31 s	C: 17, 20, 22	H-12, H-17, H-18, H-22
22	37.8 CH_2	1.70 m		H-21
		1.56 m		H-21
23	22.7 CH_2	1.69 m		
		1.56 m		
24	38.2 CH_2	1.95 m	C: 25	
25	146.0 C			
26	110.2 CH_2	4.72 brs	C: 22, 27	H-27
		4.71 brs	C: 22, 27	H-27
27	22.2 CH_3	1.65 s	C: 24, 25, 26	H-26
30	16.5 CH_3	0.97 s	C: 3, 4, 5, 31	H-2, H-6, H-31
31	27.9 CH_3	1.13 s	C: 3, 4, 5, 30	H-3, H-5, H-6, H-30, H-1 Xyl1
32	21.4 CH_3	0.78 s	C: 8, 13, 14, 15	H-7, H-15, H-17

[a]Recorded at 176.03 MHz in C_5D_5N/D_2O (4/1). [b]Recorded at 700.00 MHz in C_5D_5N/D_2O (4/1).

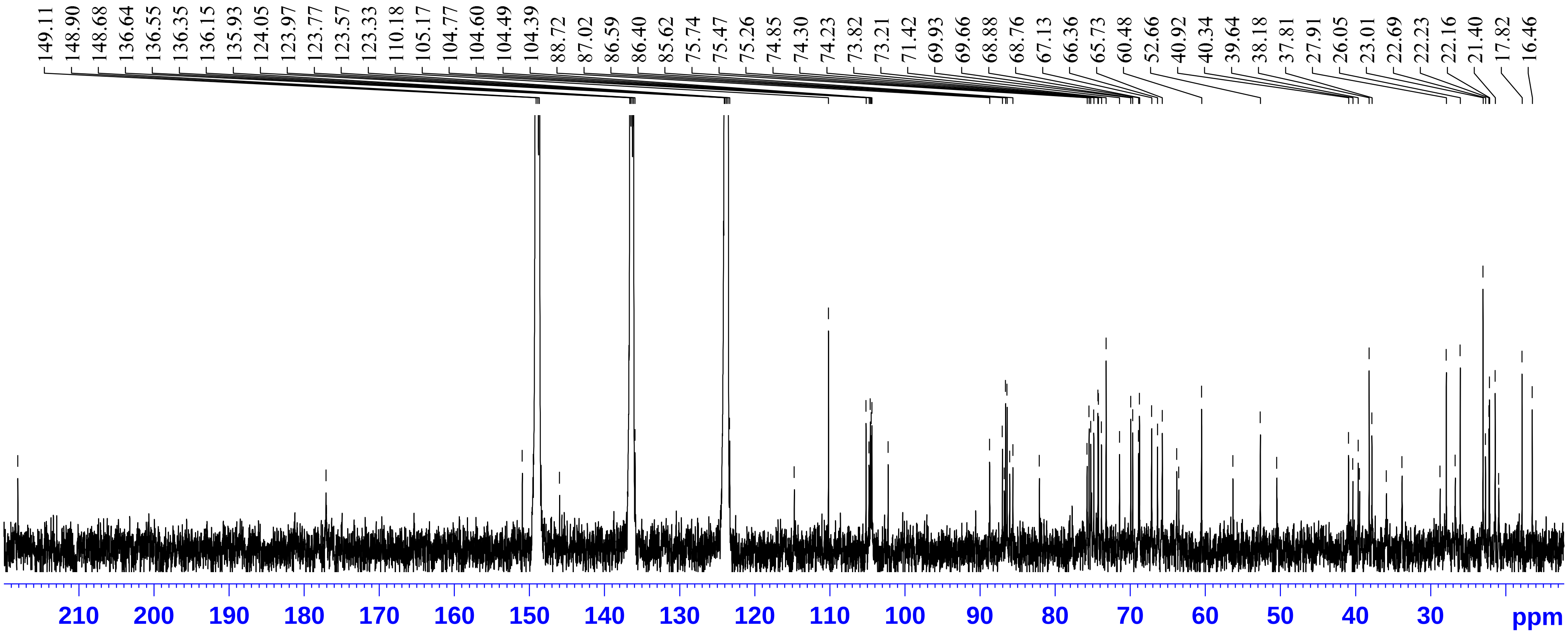

Figure S40. The ^{13}C NMR (176.03 MHz) spectrum of chitonoidoside E (**6**) in C_5D_5N/D_2O (4/1)

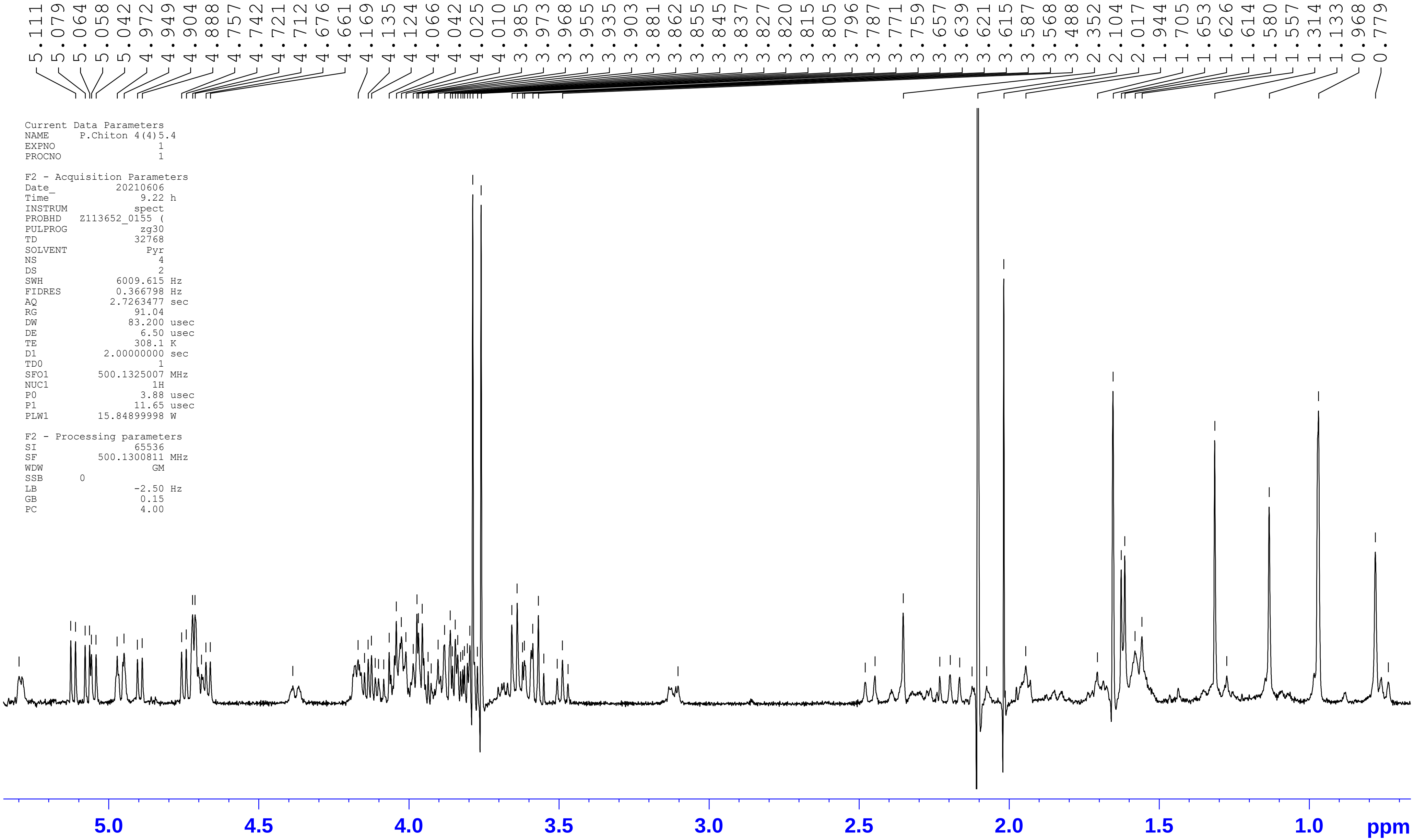

Figure S41. The ^{1}H NMR (700.00 MHz) spectrum of chitonoidoside F (**6**) in C_5D_5N/D_2O (4/1)

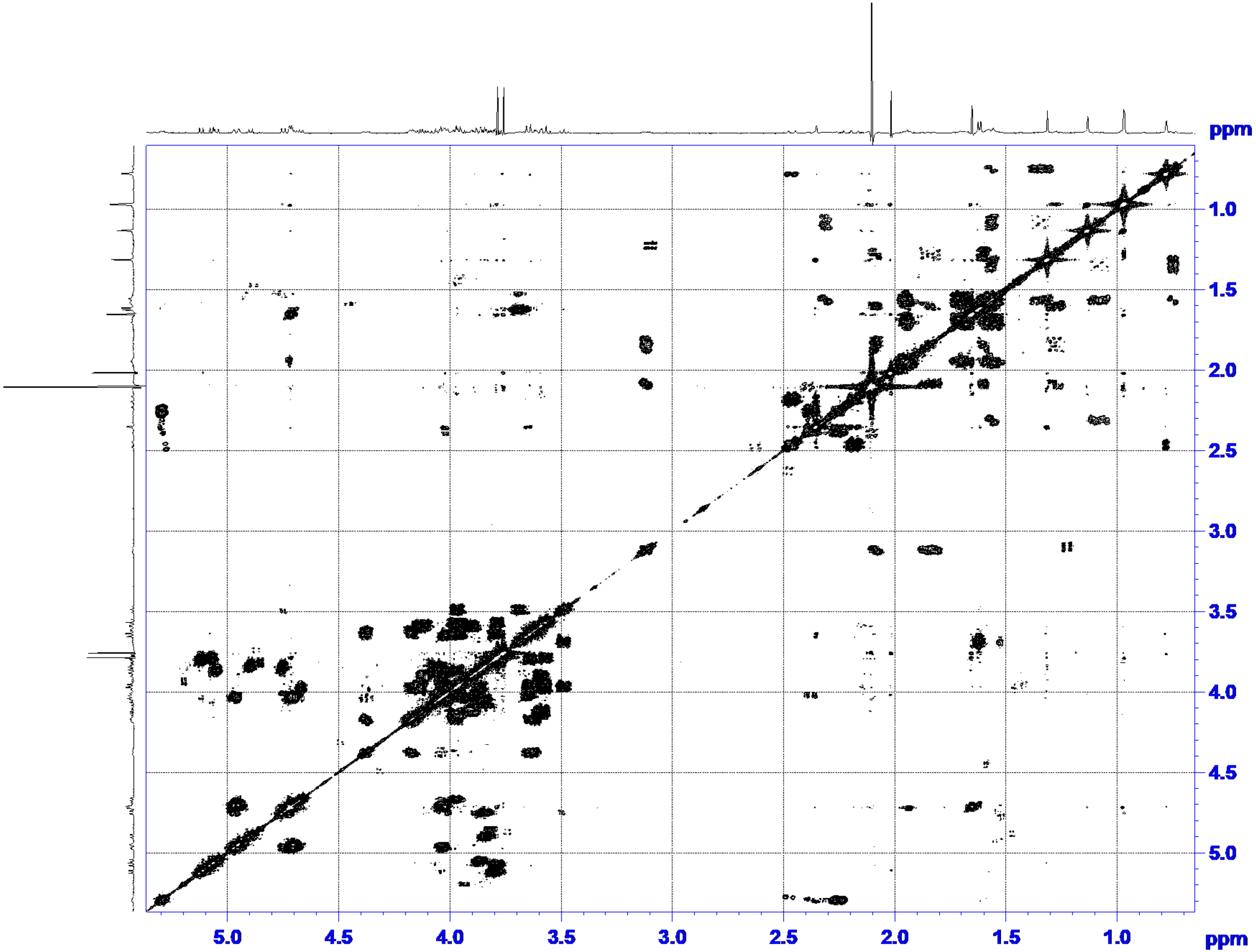

Figure S42. The COSY (700.00 MHz) spectrum of chitonoidoside E (**6**) in C_5D_5N/D_2O (4/1)

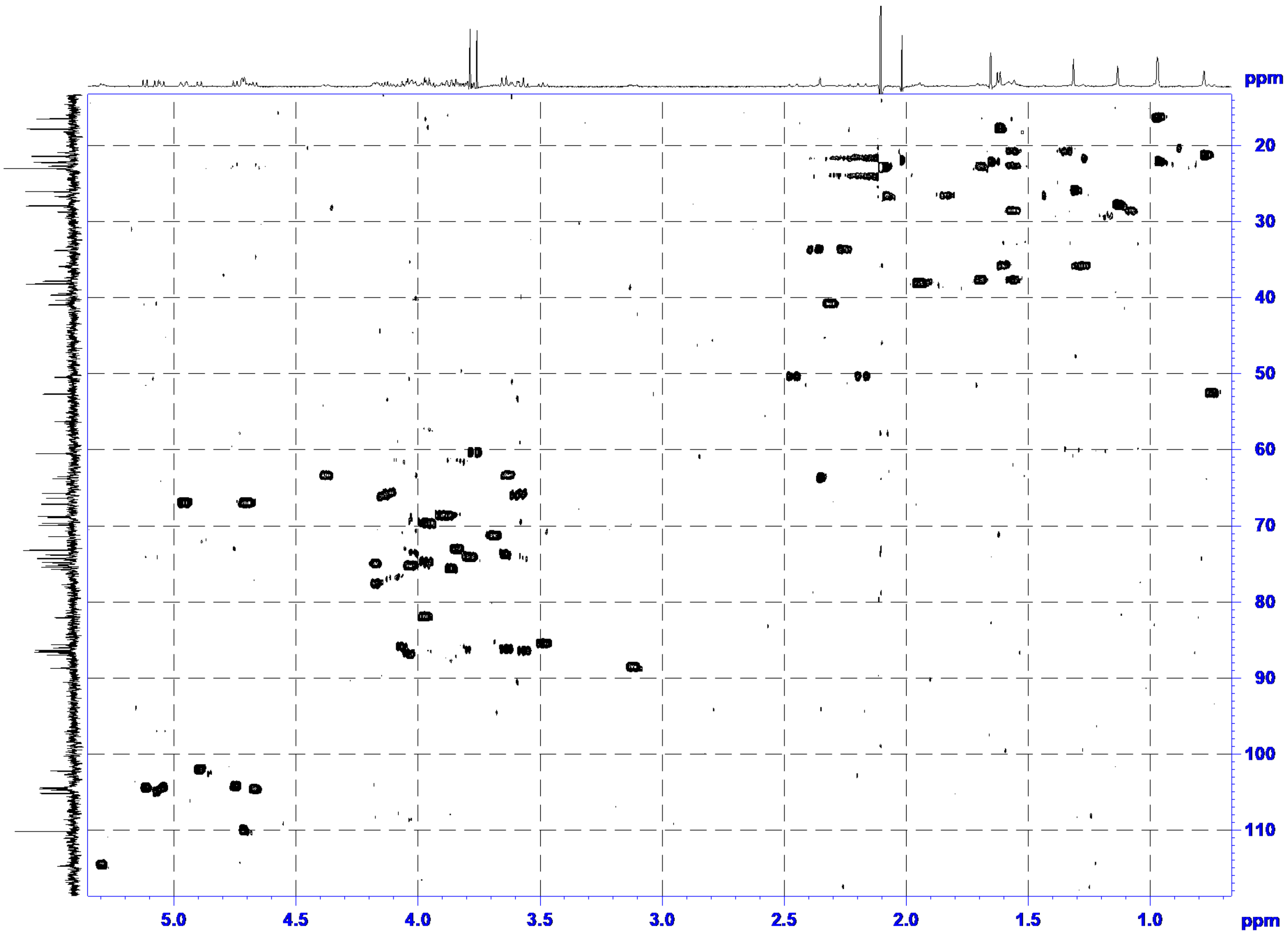

Figure S43. The HSQC (700.00 MHz) spectrum of chitonoidoside E (**6**) in C_5D_5N/D_2O (4/1)

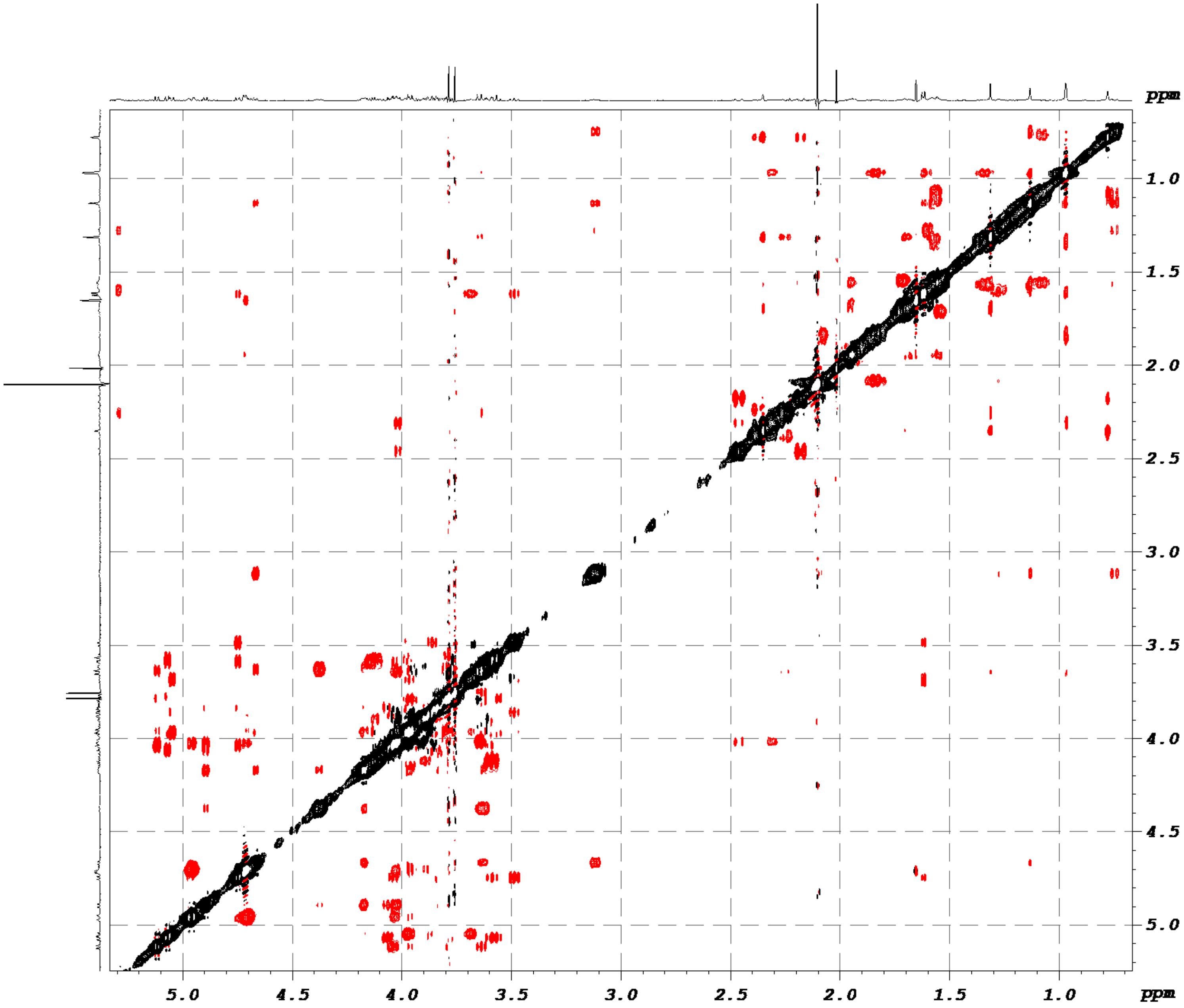

Figure S44. The ROESY (700.00 MHz) spectrum of chitonoidoside E (**6**) in C_5D_5N/D_2O (4/1)

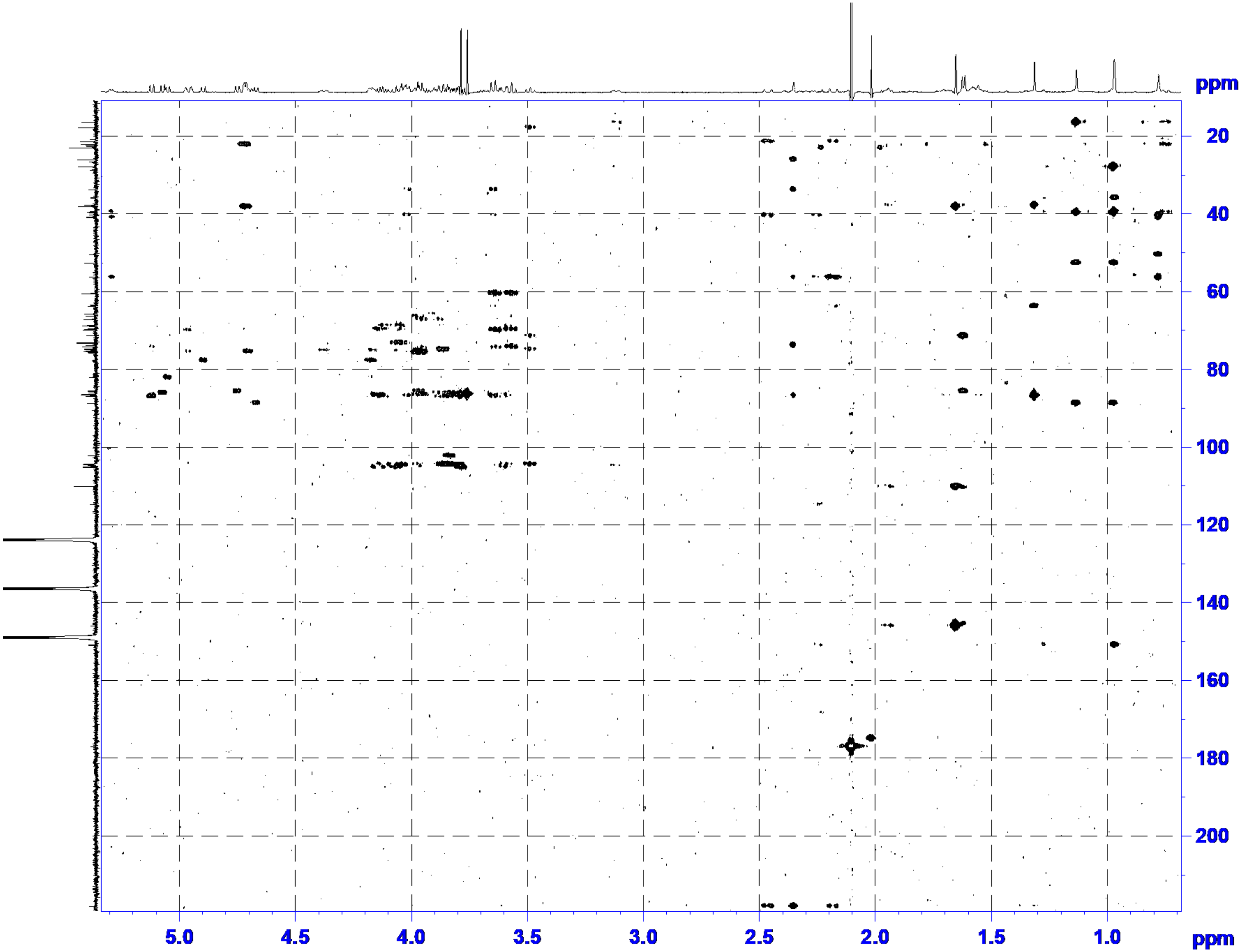

Figure S45. The HMBC (700.00 MHz) spectrum of chitonoidoside E (**6**) in C_5D_5N/D_2O (4/1)

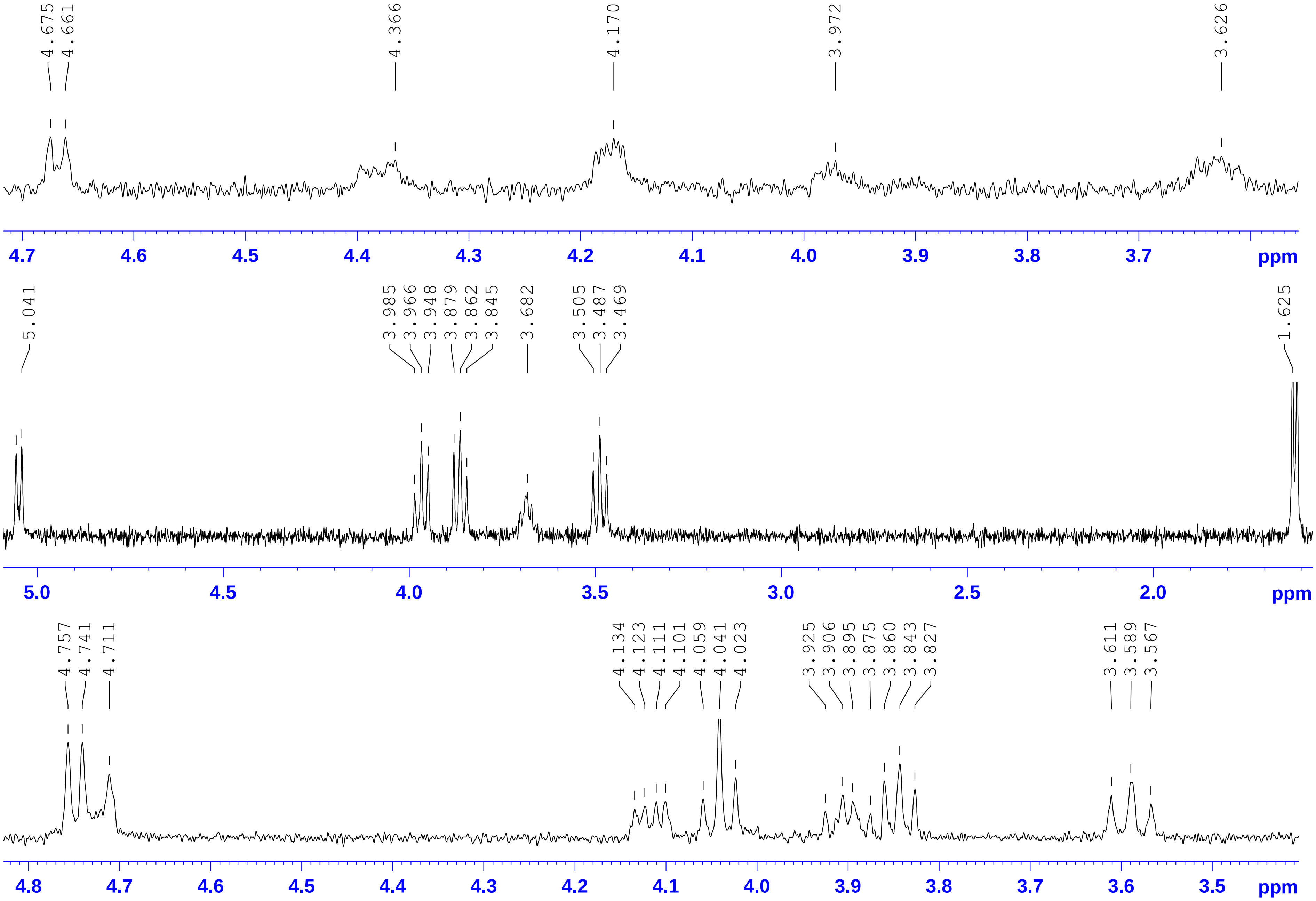

Figure S46. 1D TOCSY (700.00 MHz) spectra of Xyl1, Qui2 and Xyl3 of chitonoidoside E (**6**) in C_5D_5N/D_2O (4/1)

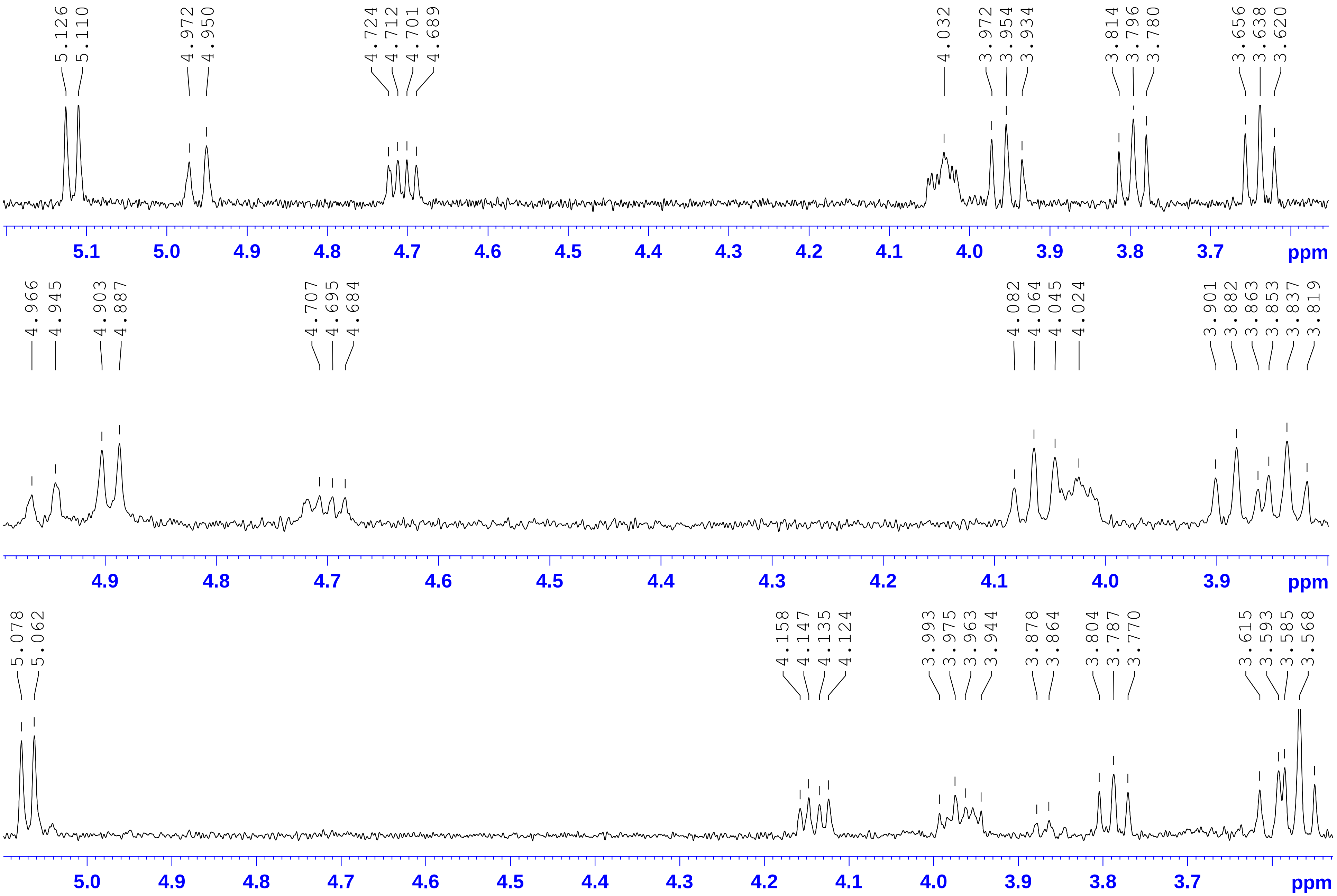

Figure S47. 1D TOCSY (700.00 MHz) spectra of MeGlc4, Glc5 and MeXyl6 of chitonoidoside E (**6**) in C_5D_5N/D_2O (4/1)

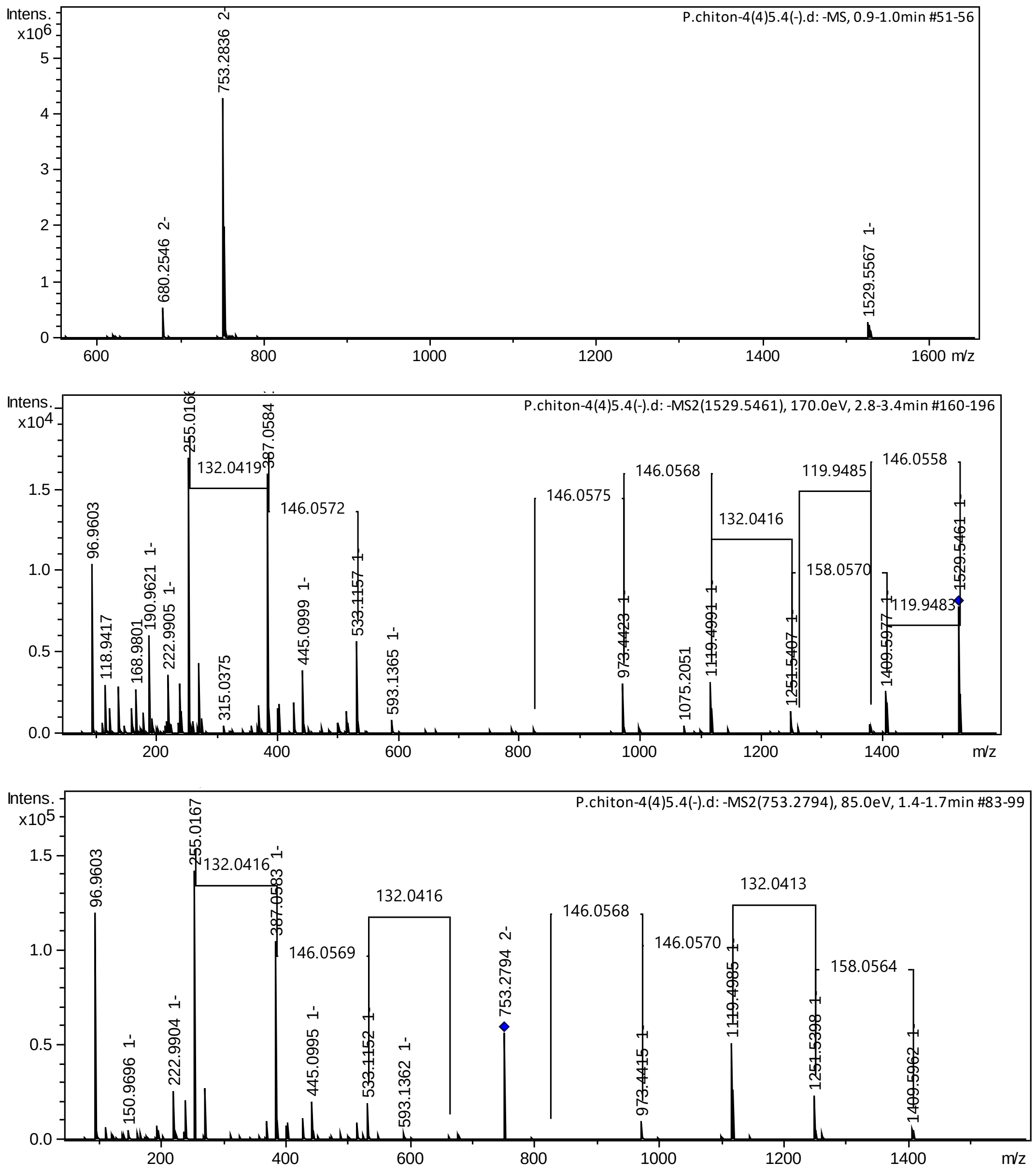

Figure S48. HR-ESI-MS and ESI-MS/MS spectra of chitonoidoside E (**6**)
